# Autoxidation Catalysis for Carbon–Carbon Bond Cleavage in Lignin

**DOI:** 10.1021/acscentsci.3c00813

**Published:** 2023-11-22

**Authors:** Nina X. Gu, Chad T. Palumbo, Alissa C. Bleem, Kevin P. Sullivan, Stefan J. Haugen, Sean P. Woodworth, Kelsey J. Ramirez, Jacob K. Kenny, Lisa D. Stanley, Rui Katahira, Shannon S. Stahl, Gregg T. Beckham

**Affiliations:** † Renewable Resources and Enabling Sciences Center, National Renewable Energy Laboratory, Golden, Colorado 80401, United States; ‡ Department of Chemical and Biological Engineering, University of Colorado Boulder, Boulder, Colorado 80309, United States; § Department of Chemistry, University of Wisconsin-Madison, Madison, Wisconsin 53706, United States

## Abstract

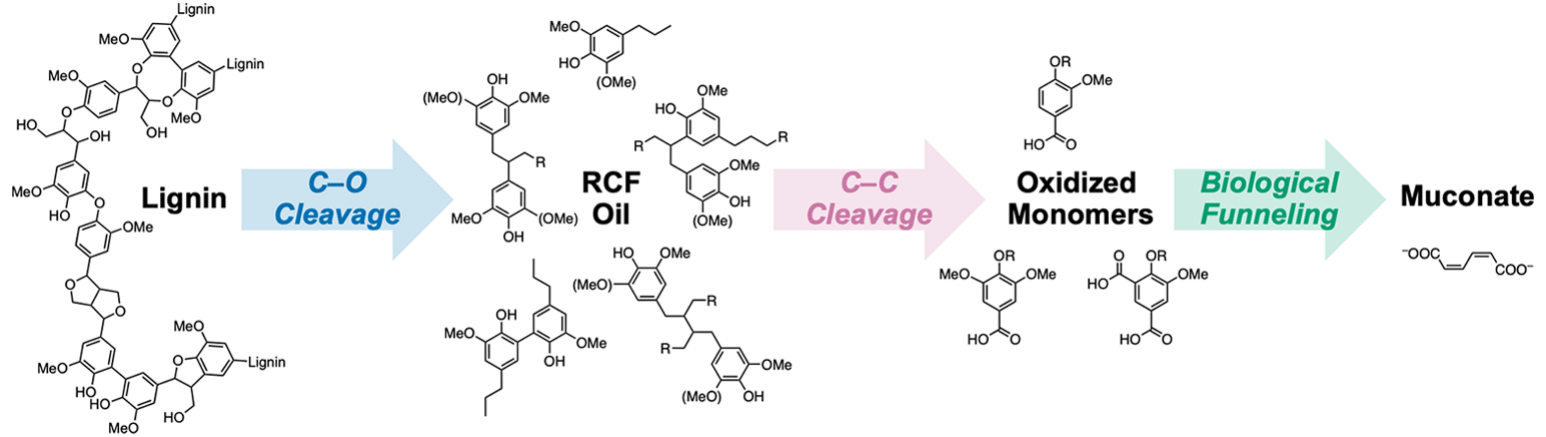

Selective lignin depolymerization is a key step in lignin valorization to value-added products, and there are multiple catalytic methods to cleave labile aryl–ether bonds in lignin. However, the overall aromatic monomer yield is inherently limited by refractory carbon–carbon linkages, which are abundant in lignin and remain intact during most selective lignin deconstruction processes. In this work, we demonstrate that a Co/Mn/Br-based catalytic autoxidation method promotes carbon–carbon bond cleavage in acetylated lignin oligomers produced from reductive catalytic fractionation. The oxidation products include acetyl vanillic acid and acetyl vanillin, which are ideal substrates for bioconversion. Using an engineered strain of *Pseudomonas putida*, we demonstrate the conversion of these aromatic monomers to *cis*,*cis*-muconic acid. Overall, this study demonstrates that autoxidation enables higher yields of bioavailable aromatic monomers, exceeding the limits set by ether-bond cleavage alone.

## Abstract 2

This work demonstrates that C−C cleavage of lignin through autoxidation catalysis enables higher yields of bioavailable aromatic monomers compared to ether-bond cleavage alone.

## Introduction

**Figure 1:**
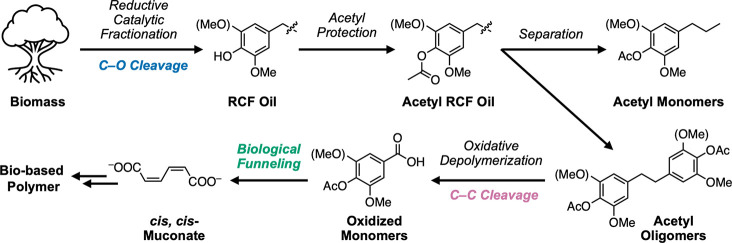
Overall lignin conversion approach presented in this work, featuring an oxidative C–C bond cleavage process to generate oxidized monomers that are suitable for biological funneling to a single product.

Lignin is one of Earth’s most abundant natural polymers, and it is synthesized via oxidative radical coupling reactions of monolignols that give rise to a polymer with aryl–ether bonds and several types of carbon–carbon (C–C) linkages.^1^ Lignin depolymerization to aromatic monomers is one of the most sought-after contemporary approaches to derive value from lignin. Many effective methods have been developed to this end,^2−8^ and near-theoretical monomer yields are now accessible, based on cleavage of β-O-4 aryl-ether linkages.^9−15^ The inability of these methods to cleave refractory C–C bonds in lignin, such as those present in the 5–5, β–1, β–β, and β–5 linkages, severely limits the aromatic monomer yield accessible from lignin (Figure 1). Hardwood lignin often exhibits high β-O-4 content and yields of aromatic monomers between 30 and 40 wt % are often attainable. Considerably lower yields are obtained from lignins with lower aryl–ether bond content, including those from softwoods, grasses, and extracted lignins from biorefinery and pulping processes.^4,16,17^

Reductive catalytic fractionation (RCF) of lignin is among the most effective methods available for conversion of lignin into aromatic monomers.^18^ However, like most lignin depolymerization methods, RCF is largely limited to aryl–ether bond cleavage and generates an oligomeric byproduct that is rich in C–C linkages. Access to higher yields of aromatic monomers from lignin necessitates methods for cleavage of the C–C bonds, such as those in β–1, β–5, β–β, and 5–5 linkages.^1,2^ Recent efforts have explored homogeneous thermal catalysis,^19^ photocatalysis,^20^ and catalytic cracking.^21^ An important advance was reported recently by Samec et al., who demonstrated a tandem RCF/oxidation sequence to increase monomer yields. Specifically, lignin oligomers obtained from RCF treatment of a birch feedstock were treated with a superstoichiometric oxoammonium reagent. This reagent, which operates via a hydride transfer mechanism, led to cleavage of linkages containing C–C bonds and selectively generated 2,6-dimethoxybenzoquinone as a product in 18 wt % yield with respect to the oligomeric feedstock.^18^

Here, we report a complementary strategy to achieve C–C cleavage in lignin that leverages catalytic autoxidation and radical reaction pathways. A cobalt/manganese/bromide cocatalyst system provides the basis for the industrial autoxidation of *p*-xylene to terephthalic acid,^22^ and analogous conditions have been used to support C–C cleavage in both simple hydrocarbons and synthetic plastics.^23−25^ Key mechanistic steps in these reactions include hydrogen atom transfer, radical trapping by O_2_, and β-scission of intermediate alkoxyl radicals. Br and oxygen-centered radicals contribute to hydrogen-atom transfer from the organic substrate in the catalytic oxidation process. The Co and Mn catalysts support the autoxidation through activation of O_2_, oxidation of HBr, and selective conversion of reaction intermediates, such as organic hydroperoxides, into the oxidized products.^26,27^ Lignin has been subjected to related conditions, but monomer yields did not exceed the monomer content of the lignin substrate.^28,29^ We postulated, however, that lignin oligomers derived from RCF treatment of biomass would be more amenable to such treatment and undergo successful conversion into aromatic monomers. The results outlined herein validate this hypothesis, showing that a Co/Mn/Br-based catalyst system converts RCF-derived oligomers from poplar into aromatic monomers, which are then used as substrates for bioconversion to *cis*,*cis*-muconate, a precursor to bioderived polymers (Figure 1).^30−35^ By controlling the catalytic conditions, the yield of aromatic products can be maximized while limiting overoxidation to quinone-based products that are not amenable to biological funneling to *cis*,*cis*-muconate. This pairing of catalytic aerobic oxidation that supports C–C bond cleavage and biological funneling offers a strategy to obtain higher yields of single products from lignin.

## Results and Discussion

### Phenol Acetylation Is Key to Enabling C–C Bond Cleavage via Autoxidation

**Scheme 1:**
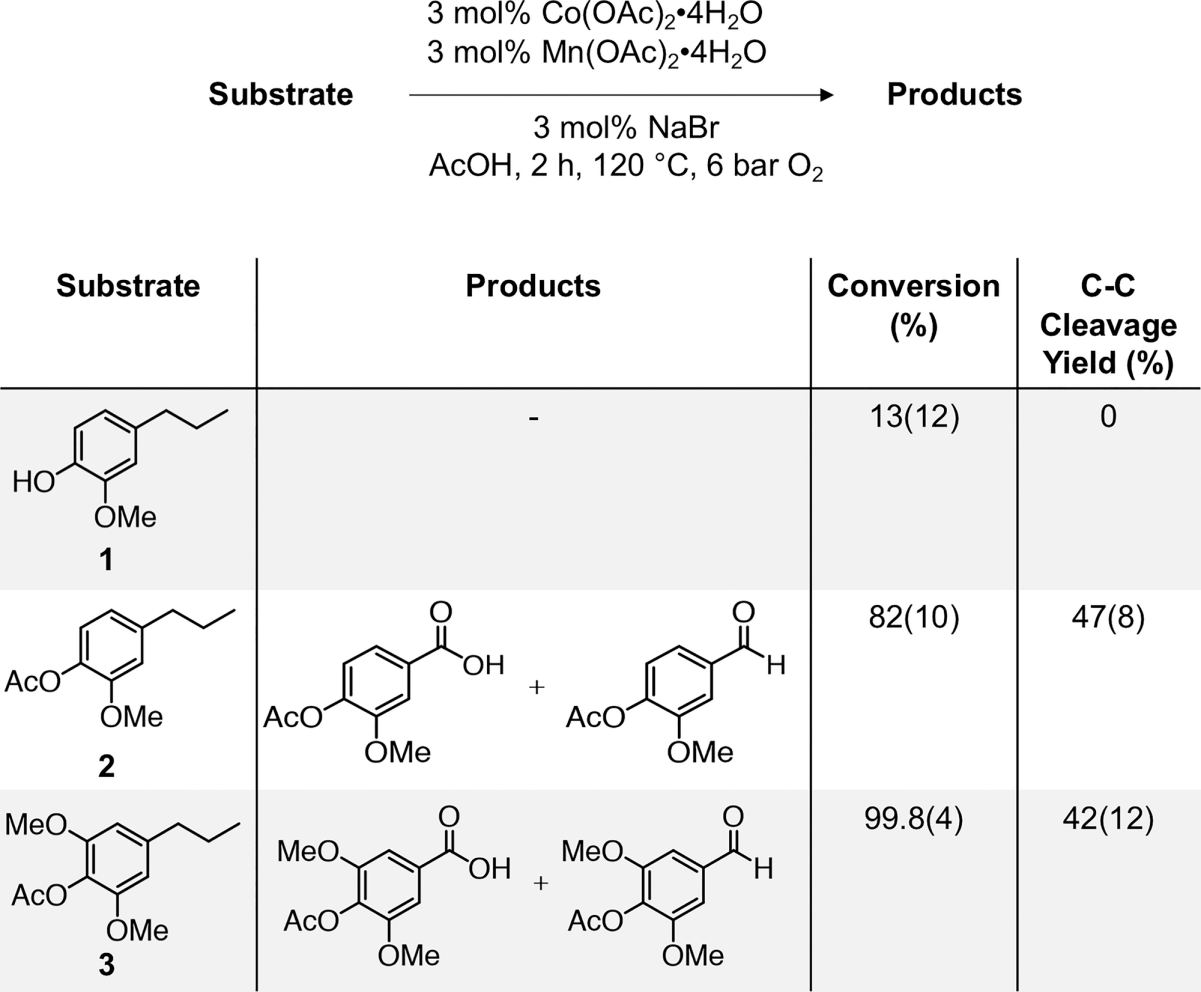
Oxidation of Model Substrates: 4-Propylguaiacol (**1**, *top*), Acetyl 4-Propylguaiacol (**2**, *Middle*), and Acetyl 4-Propylsyringol (**3**, *Bottom*) Mol % yields shown as value(standard deviation) and determined by LC-MS or UHPLC quantification.

As an initial test of the targeted C–C bond cleavage chemistry, we explored the oxidation of model aromatic substrates (Scheme 1, Tables S17 and S18 in the Supporting Information, SI). Upon subjecting 4-propylguaiacol (**1**) to catalytic Co/Mn/Br salt mixtures (3 mol % Co(OAc)_2_·4H_2_O, 3 mol % Mn(OAc)_2_·4H_2_O, 3 mol % NaBr) with heating at 120 °C for 2 h under 6 bar O_2_, we observed no C–C bond cleavage and instead recovered **1** in 87(12)% yield. This result is consistent with previous work describing the antioxidant properties of phenols^36^ and highlights the importance of phenol protection in autoxidation. In contrast, acetyl 4-propylguaiacol (**2**) can be converted to acetyl vanillic acid and acetyl vanillin in 47(8)% yield under the same oxidation conditions. Similarly, the oxidation of acetyl propylsyringol (**3**) gives a total yield of acetyl syringic acid and acetyl syringaldehyde at 42(12)%. These results on **2** and **3** demonstrate the viability of autoxidation conditions for simplified G- and S-type lignin models.

### Application of Autoxidation on Model Lignin Dimers

**Scheme 2:**
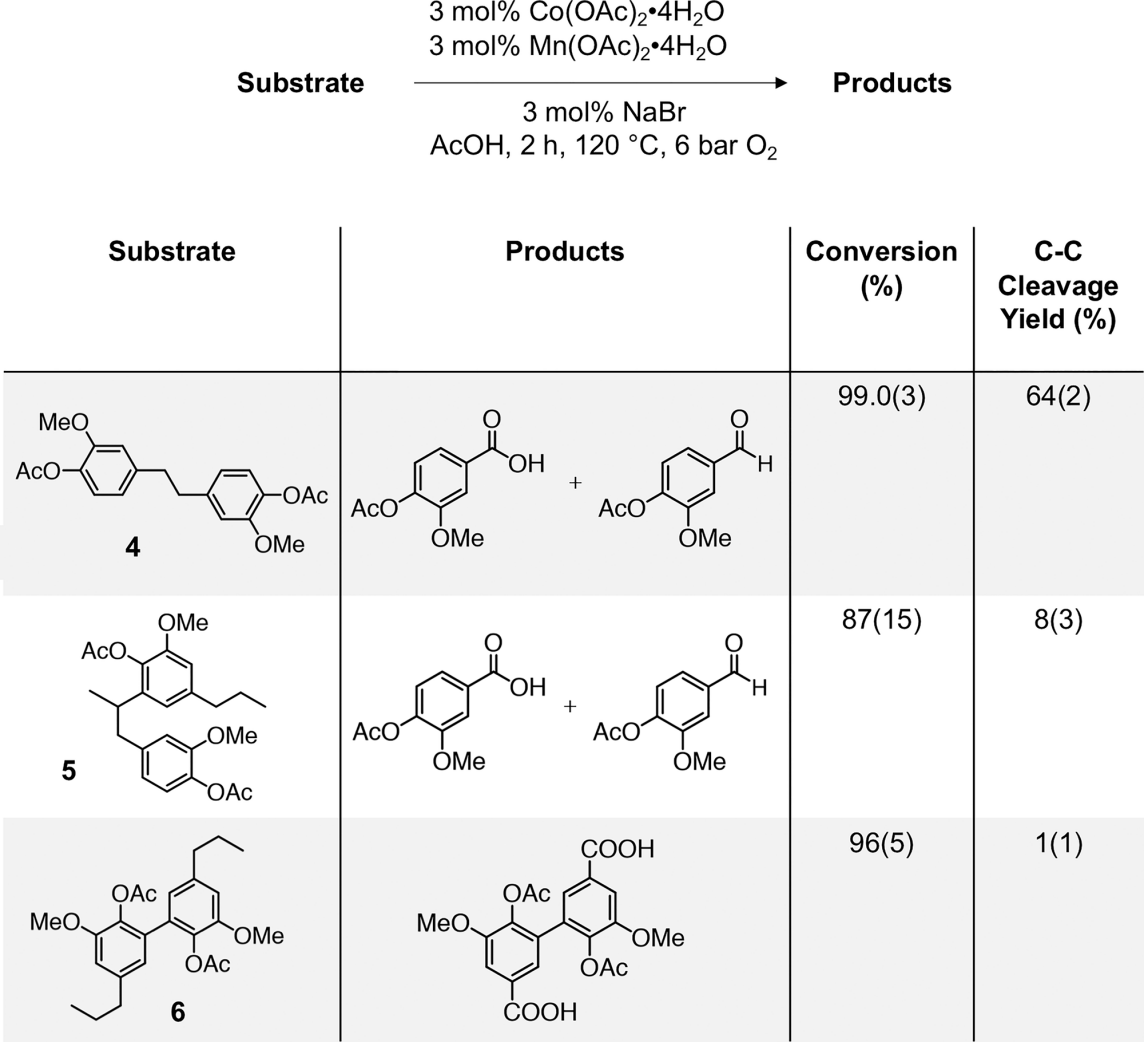
Oxidation of Model Dimer Substrates of β–1 (**4**), β–5 (**5**), and 5–5 (**6**) Linkages Mol % yields shown as value(standard deviation) and determined by LC-MS or UHPLC quantification.

We subsequently subjected various representative dimer models to the same autoxidation conditions and were able to detect monomeric products in most cases (Scheme 2, Tables S19–S21). As a representative β-1 dimer, diacetyl bivanillyl (**4**) was subjected to the same reaction conditions (3 mol % Co(OAc)_2_·4H_2_O, 3 mol % Mn(OAc)_2_·4H_2_O, 3 mol % NaBr, 120 °C for 2 h under 6 bar O_2_), which afforded C–C bond cleavage products in in 64(2)% yield. The acetyl-protected β-5 model (**5**) was oxidized to yield acetyl vanillic acid and the corresponding aldehyde in 8(3)% yield. Overall, the oxidations of compounds **4** and **5** demonstrate that C–C bond cleavage can be accessed on dimer models featuring common C–C linkages found in RCF oil. Specifically, cleavage of the C_benzylic_–C bonds is observed to form the corresponding benzoic acid monomer. For the 5–5 dimer model (**6**), full consumption of **6** was observed, but only trace products were detected.

### Preparation of a Lignin Dimer and Oligomer-Rich Stream for Autoxidation

On the basis of these promising model compound results, we sought to apply this autoxidation approach to a realistic lignin stream. RCF of biomass affords an “RCF oil” that contains monomers, but is also rich in dimers and oligomers that exhibit 5–5, β–1, β–5, and β–β linkages.^37−39^ This RCF oil provides an ideal substrate to investigate the utility of Co/Mn/Br-catalytic autoxidation to supplement the yield of aromatic monomers accessible from lignin.

RCF oil was prepared by subjecting extractives-free poplar biomass to 5 wt % Ru/C under 30 bar H_2_ in methanol for 6 h at 225 °C. RCF monomers were quantified by GC-FID to determine a total monomer content of 1.8 mol monomer/g RCF oil, Tables S1–S3. The aromatic monomer yield on a total lignin oil basis from this experiment was 34 wt %, similar to previous work.^40^ Using this oil, we functionalized free OH groups as phosphites^41^ and conducted quantitative analysis by ^31^P NMR spectroscopy, which yielded a phenolic content of 4.2 mmol/g RCF oil and an aliphatic OH content of 2.4 mmol/g RCF oil, Figure S1. With the goal of protecting the phenolic functionalities, the RCF oil was subsequently derivatized via treatment with excess acetic anhydride and pyridine at 40 °C to yield acetyl-protected RCF oil. GC-FID quantification of acetyl monomers in the acetyl RCF oil demonstrate that 78% of RCF monomers are recovered upon acetylation, Tables S4–S5. GPC traces of the RCF oil and acetyl-derivatized material exhibit very similar profiles (Figures S2–S5), suggesting that acetylation does not alter the distribution of monomer, dimer, and oligomer fractions in the oil. Treatment of the acetylated RCF oil under the phosphite OH functionalization conditions and ^31^P NMR analysis^41^ confirmed the absence of phenolic and aliphatic OH groups, Figure S1.

Subjecting acetyl poplar RCF oil, containing the full distribution of monomers, dimers and higher molecular-weight components, to 4 wt % Co(OAc)_2_·4H_2_O, 4 wt % Mn(OAc)_2_·4H_2_O, and 2 wt % NaBr at 6 bar O_2_ in acetic acid yielded 21(4) wt% of monomers after heating at 120 °C for 2 h. Acetyl vanillic acid and acetyl syringic acid are the major products formed, but the quantity of oxidation monomers following oxidation (0.13 mmol/g) is lower than the initial monomer content of the starting material (1.4 mmol/g), which may be attributable to product degradation during oxidation.

**Scheme 3:**
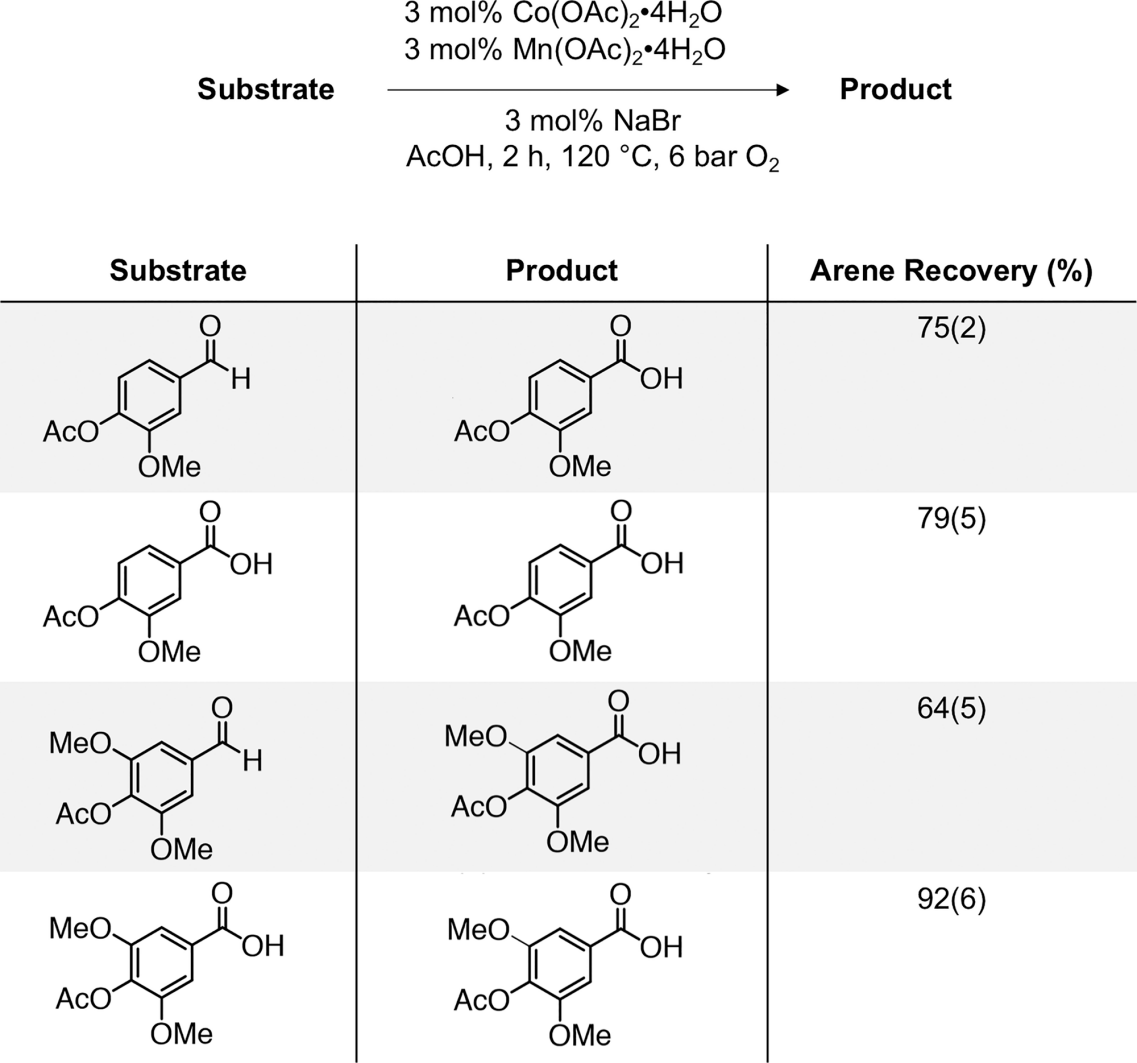
Product Stability Reactions under Autoxidation Conditions with Mol % Yields Shown As Value (Standard Deviation) Yield determined by LC-MS quantification.

To gauge the stability of the oxidation products in our autoxidation conditions, we subjected acetyl vanillin, acetyl vanillic acid, acetyl syringaldehyde and acetyl syringic acid to similar conditions with 3 mol % Co(OAc)_2_·4H_2_O, 3 mol % Mn(OAc)_2_·4H_2_O, and 3 mol % NaBr. Quantification of the acid and aldehyde products by UHPLC determined 75(2)% and 79(5)% of acetyl vanillin and acetyl vanillic acid, respectively, were recovered as acetyl vanillic acid. Similarly, acetyl syringaldehyde and acetyl syringic acid were recovered in 63(5)% and 92(6)% as acetyl syringic acid (Scheme 3; Table S6A). These data are consistent with competing degradation of aromatic species during catalytic conditions. In the oxidation mixtures of each of these four compounds, the corresponding phenolic carboxylic acid (i.e., vanillic acid or syringic acid) is detected as a minor product (<∼20%, Table S6B). Furthermore, trace quantities of methoxymaleic acid (<∼2%) are detected in the oxidation of acetyl vanillin, acetyl syringaldehyde, and acetyl syringic acid, and 2,6-dimethoxybenzoquinone is detected in the oxidation of acetyl syringaldehyde (9%) and acetyl syringic acid (3%). These data are consistent with the degradation of the aromatic rings. One possible mechanism for degradation could involve the initial deprotection of the acetyl-protected substrate to generate the phenolic congener, which can be further oxidized to form the corresponding benzoquinone derivative. Deprotection of the acetyl groups likely occurs through hydrolysis, as water is a byproduct under Mid-Century (MC) oxidation conditions.^22^ Benzoquinone species are known to undergo oxidative ring-opening to form maleic acid derivatives,^42^ which may potentially undergo further oxidation under the studied conditions.

**Figure 2:**
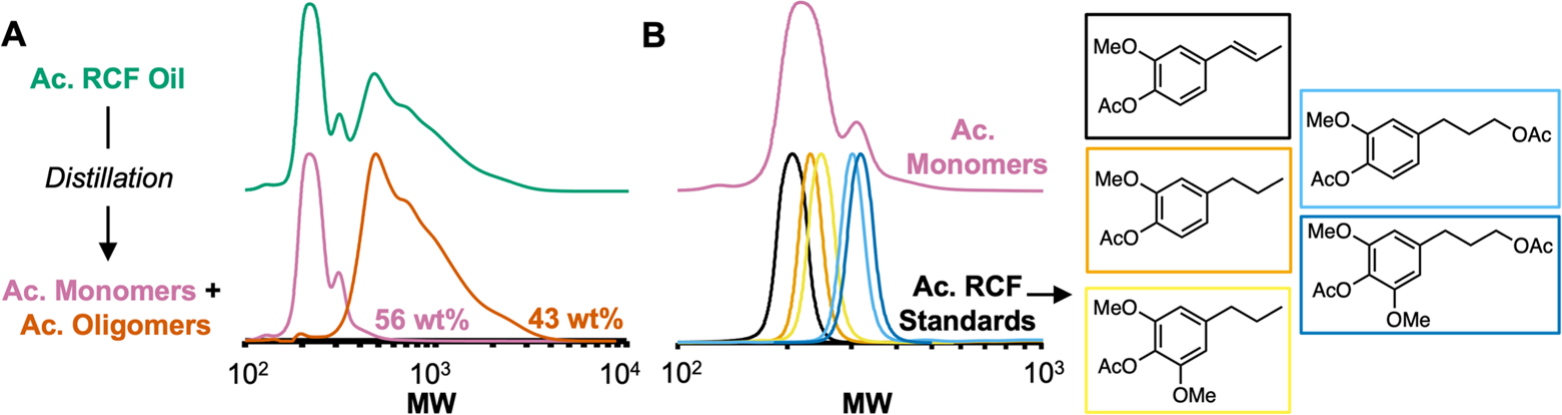
(A) GPC traces of acetyl RCF oil, acetyl monomer fraction, and acetyl oligomer fraction. Wt % values expressed as the weight of acetyl monomer or oligomer fraction/weight of acetyl RCF oil. (B) GPC trace of acetyl monomer fraction (*top*) and acetyl RCF monomer standards (*bottom*).

To circumvent the problem of monomer degradation, we separated the acetyl RCF oils monomers by vacuum distillation, as previously done by Samec et al.,^18^ and subsequently explored the oxidation of the acetyl-protected oligomers. Distillation of the acetyl RCF oil (ca. 50 mbar, 250 °C) afforded the acetyl monomer distillate as a pale-yellow oil (56 wt %), and the acetyl oligomers (43 wt %) remained in the boiling flask as a brown solid. GC-FID analysis revealed that 95% of acetyl RCF monomers were recovered in the distillate, and only trace amounts of diacetyl 4-propanolguaiacol and diacetyl 4-propanolsyringol remained the acetyl oligomer fraction (Table S5 and Figure S6). GPC analysis of the distillate (acetyl monomer fraction) confirmed the presence of only lower molecular weight species, while analysis of the acetyl oligomer fraction contains higher molecular weight compounds (Figure 2A). Furthermore, the GPC data of the acetyl monomer fraction exhibits intensity at a very similar MW range as that of authentic standards of the main monomeric components in acetyl RCF oil, consistent with the distillate being primarily composed of acetyl RCF monomers (Figure 2B). Phosphite functionalization and ^31^P NMR analysis of the acetyl monomer and acetyl dimer fractions revealed the absence of free phenolic or aliphatic OH groups, suggesting that the acetyl groups remained intact in both fractions during distillation, Figure S1. Previous studies have revealed the identity of dimers and oligomers present in the RCF-derived substrate, which mostly comprise compounds that exhibit 5–5, β–1, β–β, and β–5 linkages.^38,39^

### Autoxidation of Dimers and Oligomers in RCF Lignin Oil

**Figure 3:**
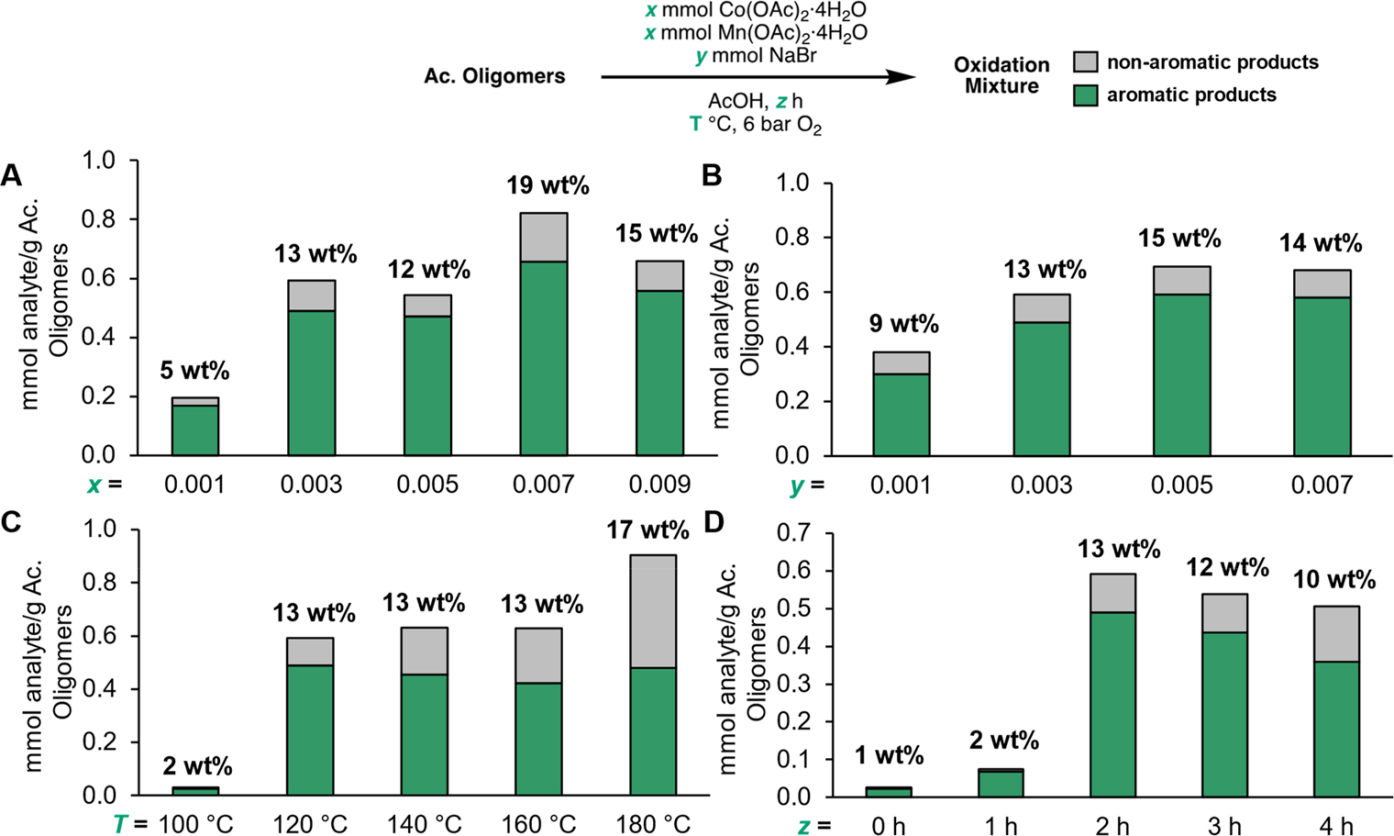
Reaction optimizations showing total yields for the autoxidation of the acetyl oligomer substrate at variable (A) Co and Mn loadings ([Co]=[Mn]), (B) NaBr loadings, (C) temperatures, and (D) reaction times. Standard conditions for optimization utilize *x* = 0.003 mmol, *y* = 0.003 mmol, *z* = 2 h, *T* = 120 °C for 20 mg of acetyl oligomer substrate. For the standard conditions, oxidation yields are an average of four runs. Values from all other conditions are from single runs. Numerical data for this figure and individual compound yields for each bar are provided in Tables S7–S10 (products quantified by GC-FID and LC-MS).

We next sought to study the effects of different reaction parameters on the oxidation of the acetyl oligomer substrate (Figure 3, Tables S7–S10). Reactions were conducted by treating the acetyl oligomer substrate with mixtures of Co(OAc)_2_·4H_2_O, Mn(OAc)_2_·4H_2_O, and NaBr, and heating the acetic acid solutions under 6 bar O_2_ gave mixtures of monomers comprising aromatic and non-aromatic structures as shown in Figure 3. The quantification data of the individual molecules are presented in the SI.

A study of Co and Mn catalyst loadings showed that increasing their loadings in a 1:1 molar ratio from 0.001 mmol to 0.003 increases the total yield of oxidation products from 5 wt % to 13 wt %. Between 0.003 and 0.009 mmol loadings, the yields range from 12 to 19 wt % (Figure 3A). An increase in monomer yield was observed upon increasing NaBr loadings from 0.001 mmol (9 wt % products) to 0.003 mmol (13 wt % products), above which the monomer yields modestly varied between 13 and 15 wt % (Figure 3B). Regarding the effect of reaction temperature, a large increase in yield was observed when the reaction was run at 120 °C (13 wt % products) compared to 100 °C (2 wt % products, Figure 3C). More carboxylic acid products (acetyl syringic acid and acetyl vanillic acid) are formed compared to the corresponding aldehyde products (acetyl syringaldehyde and acetyl vanillin) when the temperature is increased from 120 to 140 °C, but the overall yields are comparable (13 wt % products). Further increase in temperature up to 180 °C resulted in comparable overall yield of aromatic products but increased nonaromatic products, such as 2,6-dimethoxybenzoquinone. Thus, while higher temperatures enable a moderate increase in overall monomer yield, the quantity of aromatic aldehyde and carboxylic acid products compounds suitable for downstream biological funneling does not significantly increase. This may be due, at least in part, to more favorable ring-opening oxidation pathways at higher temperatures. Regarding the effect of reaction time, while product yields increase from 0–2 h reaction time, yields decrease at longer times (Figure 3D), likely also due to oxidative product degradation.

**Figure 4:**
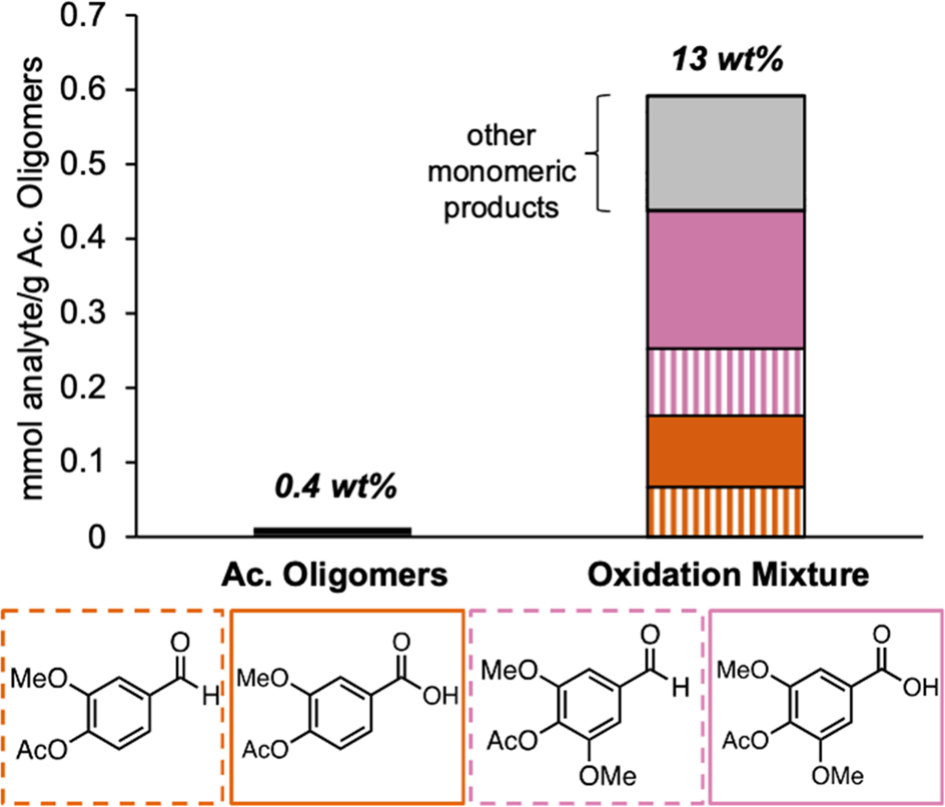
Monomer content in the starting acetyl oligomer material and resulting oxidation mixture (products quantified by UHPLC and LC-MS). Oxidation yields are an average of four runs. Wt % values expressed as the weight of total oxidation products/weight of acetyl oligomer substrate. Numerical data for this figure and the quantities of the additional monomeric products in the gray bar are provided in Table S11.

On the basis of these studies, we used 4 wt % Co(OAc)_2_·4H_2_O, 4 wt % Mn(OAc)_2_·4H_2_O, 2 wt % NaBr in acetic acid, at 120 °C for 2 h for subsequent reactions. These conditions yielded a total of 0.59(7) mmol/g of acetylated oligomers (13 wt %) of monomeric products with acetyl vanillic acid and acetyl syringic acid being the main components identified by UHPLC and LC-MS (Figure 4, Table S11). As only very small quantities of RCF monomers are present in the starting acetyl oligomer materials (0.4 wt %), the monomers generated by oxidation demonstrates the ability of these conditions to achieve C–C bond cleavage of dimers and higher molecular weight species. Overall, the average yield of additional oxidation products produced through oxidative C–C bond cleavage is 0.24 mmol/g of acetylated RCF oil. This quantity reflects a 17% increase in the acetylated monomer yield, relative to the 1.4 mmol monomers/g from the original acetylated RCF oil (eqs 1 and 2).
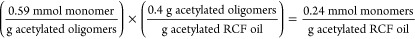
1
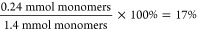
2

It is also important to note that the catalytic conditions used here with acetic acid, 2 h residence time, and 120 °C with Co/Mn/Br are directly inspired by those of the MC process to manufacture terephthalic acid from *p*-xylene at ∼80 MMT per year scale. Previous research on MC oxidation conditions indicate that acetic acid can undergo degradation to CO or CO_2_, but the losses are minimal even when run up to 175–225 °C, which, as we show in our work, is higher than the temperature needed for C–C bond cleavage in lignin.^43^ It is also known that the Co/Mn/Br oxidation catalyst can be reused for many years with little loss in activity.^22^ We are optimistic that the same beneficial features will be true for the present system.

### Biological Funneling of Oxidation Products from Acetyl RCF Oligomers

The monomers produced from the oxidation of poplar RCF oligomers resemble ideal substrates for biological funneling, wherein heterogeneous mixtures of aromatic monomers are catabolized to a single product.^30,31,33,44−46^ Previous metabolic engineering of the aromatic catabolic bacterium *Pseudomonas putida* KT2440 has demonstrated the utilization of S-type monomers (syringate and syringaldehyde) as a source of carbon and energy^47^ and the conversion of G-type monomers (vanillate and vanillin) to the value-added chemical *cis*,*cis*-muconic acid.^48^ To leverage this catabolic capability, oxidation products from poplar acetyl RCF oligomers (Figure S7; Table S12) were treated with aqueous base (NaOH) to precipitate the metal catalysts and hydrolyze acetyl groups to promote bioavailability. The resulting mixture consisted of phenolic monomers and acetate (Table S13), both of which can be catabolized by *P. putida*.^49,50^ It is important to note that *P. putida* can also grow with acetylated aromatic compounds (acetyl benzoate and acetyl vanillate) as the sole source of carbon and energy (Figure S8) and convert them to biomass or products (Figure S9), but these were not present in the base-treated substrate.

Engineered strains of *P. putida* (Table S14) were used to consume and convert the four major oxidation products in base-treated oxidation mixtures, namely syringate, syringaldehyde, vanillate, and vanillin. First, shake flask experiments with *P. putida* strain CJ486^47^ demonstrated the ability of the strain to rapidly consume all four of these as model compounds (Figure S10; Figure S11A). The same strain was then cultivated in minimal medium with 10% v/v base-treated, acetyl RCF oxidation mixtures, and all aromatic monomers were once again consumed within 24 h (Figures S11B–D).

**Figure 5:**
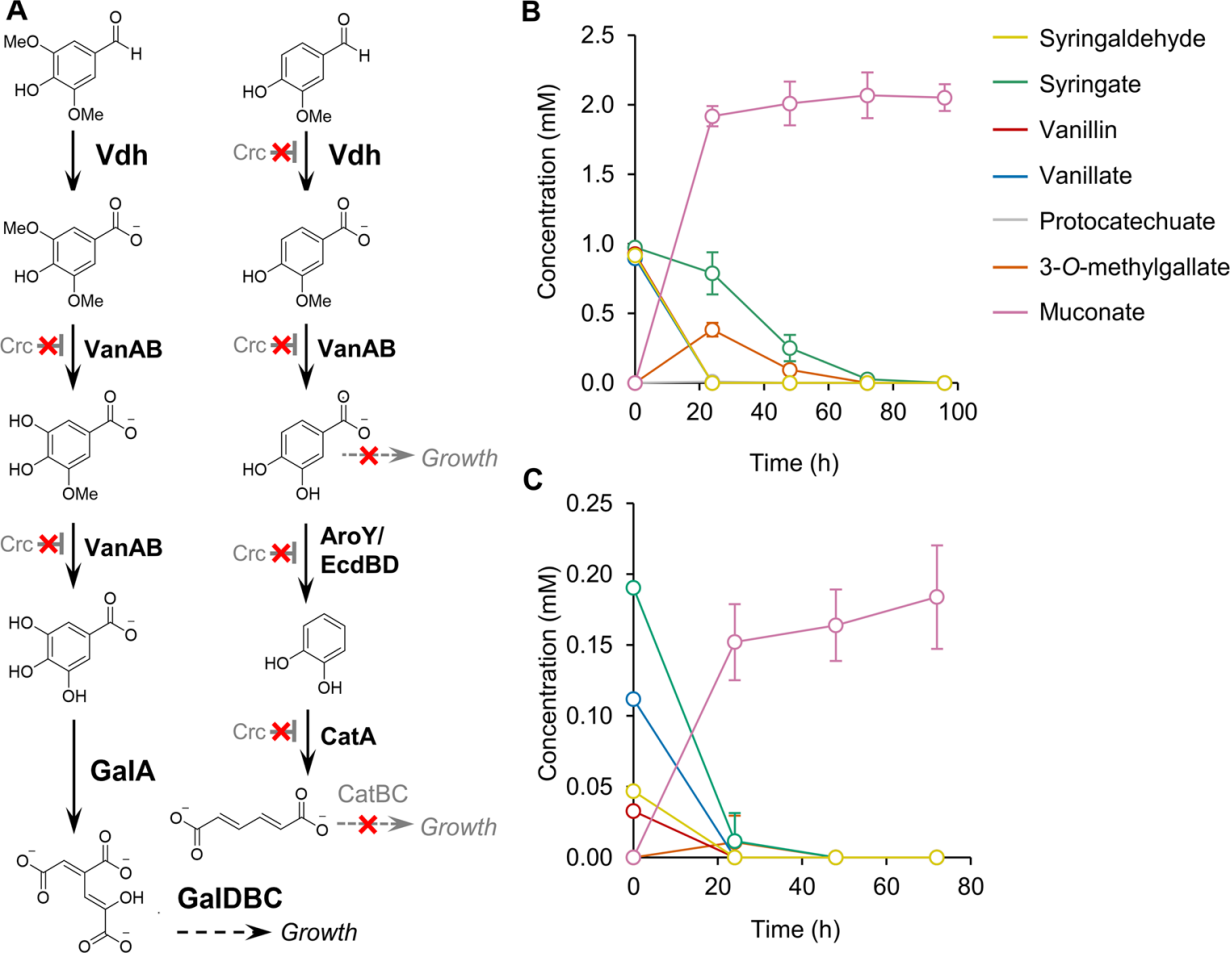
(A) Metabolic pathway for oxidation products from acetyl RCF oil oligomers in *P. putida* strain CJ781. Base treatment of the oxidation products hydrolyzed acetyl groups from aromatic monomers, yielding a mixture of syringaldehyde, syringate, vanillin, and vanillate. Syringaldehyde and syringate are converted to biomass (growth) via 3*-O*-methylgallate, gallate, and 4-oxalomesaconate. Vanillin and vanillate are converted to muconate via protocatechuate and catechol. (B) Strain CJ781 cultivated in M9 minimal medium with 1 mM/each of the model compounds syringaldehyde, syringate, vanillin, vanillate. (C) Strain CJ781 cultivated in M9 minimal medium with 10% v/v oxidation products from acetyl RCF oil. Gallate and catechol were not detected at significant concentrations in any of the experiments, and 4-oxalomesaconate was not measured. All cultures contained 5 mM glucose at time zero, and glucose was fed to a concentration of 5 mM every 24 h to support growth. Error bars represent the standard deviation from the mean of three biological replicates. Numerical data are provided in Table S15, and additional reaction replicates for the experiment in (C) are shown in Figure S12.

Next, *P. putida* strain CJ781^48^ was employed to convert these mixtures to muconate (Figure 5A). As expected, the model compounds syringate and syringaldehyde were consumed as sources of carbon and energy via 3-*O*-methylgallate (3MGA), while vanillate and vanillin were converted to muconate at 100% molar yield (Figure 5B, Figure S12), demonstrating viability of the engineered pathway. Similar outcomes were observed when strain CJ781 was cultivated in minimal medium with 10% v/v base-treated, oxidized acetyl RCF oil. Syringate and syringaldehyde were consumed with little to no accumulation of 3MGA, and vanillate and vanillin were converted to muconate at 100% molar yield for all replicates (Figure 5C; Figure S12). Furthermore, evaporation and abiotic conversion of aromatic compounds in the RCF-derived substrates was negligible, as evidenced by a lack of compositional changes in cell-free media incubated under the same conditions (Figure S13).

## Conclusions

This work demonstrates that catalytic autoxidation may be used to generate aromatic monomers from C–C linked dimers and oligomers derived from lignin. This concept is demonstrated here by acetylating phenol-rich RCF oil and then conducting aerobic oxidation of the oligomeric fraction of poplar RCF oil with a Co/Mn/Br catalyst mixture in acetic acid. This reaction yields a collection of oxygenated aromatic monomers that represent a 17% increase in monomer yield compared to the RCF process alone. Additionally, *P. putida* strains can utilize these oxidation mixtures to generate muconic acid in quantitative yield, which is a bioprivileged molecule that can be converted into performance-advantaged biopolymers and direct replacement biobased chemicals, such as adipic acid and terephthalic acid.^32,35^ While these data demonstrate the viability of catalytic autoxidation to increase monomer yields from lignin, a limitation in our current experimental setup is manifested in the product degradation chemistry that is likely occurring in parallel to the productive C–C bond cleavage. One possible pathway for arene degradation may proceed via a phenolic intermediate, as related ring-opened products have been reported in various oxidative degradation reactions of phenols,^51−55^ and the detected dimethoxybenzoquinone and methoxymaleic acid products may result from such a reaction. To overcome this limitation, flow chemistry could allow for improved control over the reaction conditions and residence time, thereby enabling improved selectivities and yields to aromatic products.

## Methods

See SI for full details of the methods and experimental ^1^H and ^13^C NMR spectra (Figures S14–66). Poplar RCF oil was prepared by hydrogenolysis in methanol at 225 °C over a Ru/C catalyst. Acetylation of poplar RCF oil was accomplished by treating the poplar RCF oil with acetic anhydride and pyridine at 40 °C. Vacuum distillation at 250 °C afforded an acetylated monomer distillate fraction and the acetylated oligomer fraction which remained in the distilling flask. Oxidations of the acetylated oligomer fraction were performed in 75 mL stainless steel vessels from the Parr Instrument Company. Analysis of products were conducted using GC-FID (RCF substrates, oxidation products for oligomer oxidation optimization screening in Figure 3), UHPLC (products of model compound oxidation and the optimized oligomer oxidations in Figure 4), and LC-MS (model compound and oligomers oxidations).

## Data Availability

The data sets in this article are provided in full in the SI.

## Supporting Information Available

Detailed methods for lignin oxidation catalysis, microbial conversion of oxidation mixtures, and product quantification, and the synthesis and characterization of lignin model compounds, analytical standards, and acetyl RCF substrates (PDF)
Transparent Peer Review report available (PDF)
