# Supplementary material for: Autoxidation Catalysis for Carbon–Carbon Bond Cleavage in Lignin
Source: ACS Cent Sci. 2023 Nov 22;9(12):2277–85. doi: 10.1021/acscentsci.3c00813 (PMC10755848; doi:10.1021/acscentsci.3c00813)
Supplement: Supplementary file 1 — oc3c00813_si_001.pdf [file oc3c00813_si_001.pdf]

Supporting Information for:

Autoxidation catalysis for carbon–carbon bond cleavage in lignin

Nina X. Gu,[a] Chad T. Palumbo,[a] Alissa Bleem,[a] Kevin P. Sullivan,[a] Stefan J. Haugen,[a] Sean P. Woodworth,[a] Kelsey J. Ramirez,[a] Jacob K. Kenny,[a,b] Lisa D. Stanley,[a] Rui Katahira,[a] Shannon S. Stahl,[b,]* Gregg T. Beckham[a,]*

[a] Renewable Resources and Enabling Sciences Center, National Renewable Energy Laboratory, Golden, CO, USA

[b] Department of Chemical and Biological Engineering, University of Colorado Boulder, Boulder, CO, USA

[c] Department of Chemistry, University of Wisconsin-Madison, Madison, WI, USA

* Email: gregg.beckham@nrel.gov, stahl@chem.wisc.edu

Table of Contents

General Experimental Methods S2
Synthetic Methods for Standards and Model Compounds S4
Preparation of RCF Substrates S8
RCF Oil Oxidation Quantification Data S14
Biological Conversion S20
NMR Spectra. S27
References S46

General Experimental Methods

General considerations: 4-propenylsyringol,[1] methoxymaleic acid,[2] and the β-5 dimer model (precursor to **6**)[3] were prepared from published procedures. All other reagents were purchased from commercial vendors and used without further purification unless otherwise stated. Poplar chips used to prepare the RCF oil utilized in this study has been previously described.[1] 75 mL reactors used for the oxidation reactions were purchased from Parr and treated with a Dursan® inert coating by Silicotek.

GC-FID quantification of RCF monomers: The GC-FID method for monomer quantification has been previously described,[1] and the following analytes were quantified in this study by preparing calibration curves from authentic standards: 4-ethyl guaiacol, 4-propyl guaiacol, isoeugenol, 4-propyl syringol, 4-propanol guaiacol, isoelemicin, and 4-propanol syringol.

GC-FID quantification of acetyl RCF monomers: A 1 µL injection was used with a split ratio of 10:1. A 3 0m x 250 µm x 0.25 µm Agilent Technologies HP-5 column was used. The inlet temperature was set to 260 °C, and the oven was ramped as follows: initial temperature of 100 °C (hold time = 0 min), ramp at 50 °C/min to 120 °C (hold time = 7 min), ramp at 10 °C/min to 150 °C (hold time = 1 min), ramp at 50 °C/min to 200 °C (hold time = 2 min), ramp at 50 °C/min to 300 °C (hold time = 1 min). A flame ionization detector was used to quantify the products, and calibration curves were generated for the analytes based on peak area ratios between products and a biphenyl internal standard at 500 ppm. The following analytes were quantified from authentic standards: acetyl methylguaiacol, acetyl ethylguaiacol, acetyl propylguaiacol, acetyl isoeugenol, acetyl propylsyringol, acetyl propenylsyringol, diacetyl propanolguaiacol, diacetyl propanolsyringol.

GC-MS analysis: Samples were analyzed on an Agilent GC-MS instrument (GC model 7890A, MS model 5975C) equipped with a Polyarc system. An Agilent HP-5ms Ultra Inert column was used, and the same temperature ramp was used as the corresponding GC-FID analysis.

Analysis of autoxidation products by ultra-high performance liquid chromatography – tandem mass spectrometry. Calibration standards and samples were analyzed with an Agilent 1290 series UHPLC system paired with an Agilent 6470A triple quadrupole mass spectrometer. Ionization was performed by Agilent's jet stream electrospray ionization technology in both positive and negative modes. Each compound was optimized for multiple reaction monitoring (MRM) quantifier and qualifier transitions prior to analysis. These transitions as well as respective collision energies and fragmentor voltages are presented in table (**Table S16**). A Phenomenex Kinetex 1.7 µm, 2.1 × 100 mm C18 column held at 55 °C was used as the stationary phase for injections of all standards and samples at an injection volume of 0.5 µL. Separation was enabled by mobile phases consisting of (A) 0.1% formic acid in water and (B) 0.1% formic acid in methanol held at a constant flow rate of 0.5 mL/min and at an initial condition of 97.5% : 2.5% (A:B). The following gradient was utilized for chromatography: 0-0.5 min were diverted to waste under initial conditions, 0.5-3 min held at initial conditions before ramping to 100% B at 15 min. At 15.01 min, initial conditions were restored and held to final run time of 17 min. Source parameters were configured as follows for both negative and positive polarity: capillary voltage 3.5 kV, nozzle voltage 0.5 kV, drying gas temperature 300 °C, drying gas flow 7 L/min, sheath gas temperature 350 °C, sheath gas flow 11 L/min, and nebulizer gas pressure set to 35 psi. All analytes were quantified using an external curve with an r^2 coefficient of ≥ 0.995 and quadratic fit. A calibration verification standard was run every twenty samples to monitor instrument drift and ensure it did not deviate by more than +/-15% from the prepared concentration over the course of the run. Analysis of chromatography and data was accomplished using Agilent MassHunter Quantitave Analysis (QQQ) version B.08.00 software.

UHPLC analysis of acetylated monomers products and bioconversion substrates and products. Acetylated monomer products from autoxidation experiments and the substrates and products for bioconversion experiments were quantified by ultra-high performance liquid chromatography (UHPLC) coupled with a diode array detection. Samples and standards were analyzed using an Agilent 1290 series UHPLC system equipped with a G7117A diode array detector (DAD). Analysis was achieved using an injection volume of 0.5 µL utilizing a Phenomenex Kinetex C18 1.7 µm, 2.1 × 100 mm column held at 35 °C and mobile phases comprised of (A) water with 0.16% formic acid and (B) acetonitrile. Chromatographic separation was carried out with a flowrate held constant at 0.8 mL/min by the following gradient program: initial-0.5 min 97.5% (A), 2.5% (B); 0.5-2.17 min 90% (A), 10% (B); 2.17-3.5 75% (A), 25% (B); 3.5-5 a ramp to 73% (A), 27 % (B) and held for 0.5 min to 5.5 min and finally a return to initial conditions at 5.51 min to 97.5% (A), 2.5% (B) that was then held for a total runtime of 7 min. Detection of analytes was achieved by DAD with wavelengths 265 nm and 280 nm monitored. Standards were acquired from commercial vendors for all

compounds except for *cis, trans*-muconic acid which was prepared according to the procedures detailed,[4] and calibration curves were run for each analyte. Gallic acid, protocatechuic acid, vanillic acid, acetyl syringic acid, acetyl vanillin and *cis, cis*-muconic and *cis, trans*-muconic were monitored and quantitated using 265 nm while catechol, syringic acid, vanillin, syringaldehyde, acetyl vanillin, acetyl syringaldehyde and 3-*O*-methylgallic acid were analyzed using the wavelength 280 nm. All calibration curves were linear with r^2 coefficients ≥ 0.995 with a quantitation range of 1-500 μg/mL for all analytes except for the acetyl compounds. Acetyl syringic acid and acetyl vanillic acid had a linear range of 1-250 μg/mL and acetyl vanillin and acetyl syringaldehyde with a range of 1-100 μg/mL. A calibration verification standard was run every 10-20 samples to monitor instrument drift to ensure accurate quantitation.

NMR spectroscopy: ^{1}H and ^{13}C NMR spectra were acquired using either a Bruker Avance NeoNanobay 300 MHz spectrometer (7.05 T) equipped with a room temperature BBO (broad band optimized) 5 mm probe head, or a Bruker Avance III HD Nanobay 400 MHz instrument. Unless otherwise stated, all ^{13}C spectra were measured at 25 °C using a 90° pulse angle, inverse-gated decoupling, 1,024 scans, and a recycle delay of 2 seconds, and all ^{1}H spectra were measured at 25 °C using a 90° pulse angle, 16 scans, and a recycle delay of 5 seconds.

Methyl-derivatization of samples for GC-FID quantification of optimization of oxidation conditions: A reaction mixture aliquot of known mass (4 mL of reaction mixture, *ca.* 5 mg with respect to substrate) was concentrated to dryness. To the resulting material, K_2CO_3 (0.05 g), MeI (0.05 g), and MeCN (0.95 mL) were added, and the reaction mixture was stirred vigorously at 25 °C for 3 h. A MeCN stock of biphenyl (10,000 ppm, 50 μL) was added to mixture as the internal standard (500 ppm in sample), and the mixture was subsequently filtered into an amber GC vial and analyzed.

^{31}P NMR analysis of OH content: Analysis of aliphatic, phenolic, and carboxylic hydroxyl groups was performed using previously reported methods.[5] An internal standard solution of $CDCl_3$, triphenylphosphine oxide, and $Cr(acac)_3$ was prepared. ~20-30 mg of the lignin sample was added to a 4 mL vial. Approximately 0.6 mL of the internal standard solution was then added to the sample, followed by 0.9 mL of anhydrous pyridine, forming a homogeneous solution. Next, approximately 0.2 mL of the derivatizing reagent, 2-chloro-4,4,5,5-tetramethyl-1,3,2-dioxaphospholane (TMDP) was added to each sample. The samples were capped, and shaken briefly to fully mix, and inspected to verify no precipitate had formed, and subsequently transferred into NMR tubes. ^{31}P NMR spectra were acquired on a Bruker Avance III HD Nanobay 400 MHz instrument equipped with a nitrogen-cooled Prodigy cryoprobe using an inverse gated decoupling pulse sequence, 25 second pulse delay, and 128 scans at 25 °C with a LB of 5.0 Hz.

Gel permeation chromatography: Samples (*ca.* 5-20 mg) were acetylated using 0.5 mL pyridine (Sigma-Aldrich, anhydrous 99.8%) and 0.5 mL of acetic anhydride (Sigma-Aldrich, reagent plus ≥ 99%) sealed and heated to 40 °C for 24 hours while stirring. Subsequently, 1 mL aliquots of methanol were then added to each sample and dried under N_2. This was repeated five times. Samples were then dried under vacuum at 40 °C overnight. Samples were then diluted in THF and stirred for 30 min. The THF solution is filtered through a 0.2 μm syringe filter into an HPLC vial. 20 μL of sample is injected on an HPLC fitted with three PLgel 7.5 x 300 mm columns in series: 10 μm x 50 Å, 10 μm x 103 Å, 10 μm x 104 Å (Agilent Technologies, Stockport, UK) at ambient temperature with an isocratic 1 mL min^{-1} 100% tetrahydrofuran (Sigma-Aldrich, inhibitor-free ≥ 99.9%) for 45 min. Analytes were monitored at 210 nm, 260 nm, and 270 nm on the DAD. MW was calibrated against polystyrene standards.

<u>Oxidation catalysis:</u>

Standard oxidation of the model compounds: An acetic acid solution of substrate (0.1 mmol) was transferred to a Parr reactor containing a glass liner and teflon stir bar, and acetic acid solutions of $Co(OAc)_2 \cdot 4H_2O$ (0.7 mg, 0.003 mmol), $Mn(OAc)_2 \cdot 4H_2O$ (0.7 mg, 0.003 mmol), and NaBr (0.3 mg, 0.003 mmol) were subsequently added. An additional portion of acetic acid was added such that the total reaction volume was 15 mL. The Parr reactor was then sealed and pressurized with 29 bar air and 31 bar He at room temperature. The reaction vessels were subsequently heated at 120 °C for 2 h (reaction time not including an initial heating period of *ca.* 30 min), with stirring at 700 rpm. At the end of the reaction, the reactors were cooled in an ice bath and then depressurized. For product quantification, the combined weight of the Parr reactor, glass liner and stir bar was measured prior to the reaction, and the combined weight of the final reaction mixture, Parr reactor, glass liner and stir bar was measured at the end of the reaction. The final reaction mixtures were homogeneous solutions with no apparent precipitate formation. Aliquots of the reaction mixture of known mass were then analyzed by GC or LC-MS.

Standard oxidation of the acetyl oligomer fraction: An acetic acid solution of acetyl oligomer substrate (0.02 g) was transferred to a Parr reactor containing a glass liner and stir bar, and acetic acid solutions of $Co(OAc)_2 \cdot 4H_2O$ (0.7 mg, 0.003 mmol), $Mn(OAc)_2 \cdot 4H_2O$ (0.7 mg, 0.003 mmol), and NaBr (0.3 mg, 0.003 mmol) were subsequently added. An additional portion of acetic acid was added such that the total reaction volume was 15 mL. The Parr reactor was then sealed and pressurized with 29 bar air and 31 bar He at room temperature. The reaction vessels were subsequently heated to 120 °C for 2 h (reaction time not including an initial heating period of *ca*. 30 min), with stirring at rpm 700. At the end of the reaction, the reactors were cooled in an ice bath and then depressurized. For product quantification, the combined weight of the Parr reactor, glass liner and stir bar was measured prior to the reaction, and the combined weight of the final reaction mixture, Parr reactor, glass liner, and stir bar was measured at the end of the reaction. The final reaction mixtures were homogeneous solutions with no apparent precipitate formation. Aliquots of the reaction mixture of known mass were then analyzed by GC or UHPLC.

Preparation of bacteria culture media: Oxidation reactions were performed in triplicate at 40 mg scale in scintillation vials. A portion of each sample was removed for analysis, leaving ~30 mg of starting material per sample, the compositions of which are presented in **Figure S7**. Samples were dried with a nitrogen stream and stored at -20 °C until use. To prepare the substrates for bacterial cultivation, 3 mL of water and 1.2 mL of aqueous 4 M NaOH was added to each lignin sample, and mixtures were solubilized with a stir bar for 1 h at room temperature. Metal catalyst visibly precipitated and was removed by centrifuging the samples at 3000 *g* for 5 min. Supernatants were transferred to 25 mL flasks with stir bars and neutralized by dropwise addition of 38% HCl. Each preparation was then sterilized by passing through a 0.2 μm syringe filter. Culture media were prepared by combining 4.1 mL of sterile RCF oxidation substrate with 36.9 mL of sterile 1.1X M9 medium to achieve a final concentration of 10% v/v RCF oxidation substrate in 1X M9 medium 6.78 g/L Na_2HPO_4, 3 g/L KH_2PO_4, 0.5 g/L NaCl, 1 g/L NH_4Cl, 2 mM $MgSO_4$, 100 μM $CaCl_2$, and 18 μM $FeSO_4$). For control media, 40 mM stock solutions of vanillate, syringate, vanillin, and syringaldehyde were prepared by mixing each aromatic compound in water and titrating with 4 M aqueous NaOH to neutralize the solution and dissolve the compound. Aromatic stocks were then combined with 1.1X M9 medium to reach a final concentration of 1 mM each. Vanillic acid, syringaldehyde, and vanillin were obtained from Sigma-Aldrich, and syringic acid was obtained from AK Scientific.

Shake flask cultivations: *Pseudomonas putida* strains CJ781 and CJ486 (**Table S14**) were streaked from glycerol stocks onto LB agar (Sigma-Aldrich). The next day, biological triplicate cultures of CJ781 were prepared in 5 mL of LB medium (Sigma-Aldrich) in 15 mL glass culture tubes, using a single colony to inoculate each tube. A single culture of CJ486 was prepared in the same manner. Cultures were incubated overnight (~16 h) at 225 rpm and 30 °C. Overnight cultures were then centrifuged at 2500 *g* for 5 min and resuspended in 2 mL each of 1X M9 medium. For each sterile medium described above, 8 mL of medium was added to each of five 50 mL culture flasks (25 flasks in total). Sterile glucose solution was added to a concentration of 5 mM. Flasks were then inoculated with relevant overnight culture suspensions to an initial OD_{600nm} of 0.1. For each condition, three biological replicate flasks were prepared with CJ781, one flask was prepared with CJ486, and one flask remained uninoculated as an abiotic control. All flasks were sealed with Breathe-Easy film (Diversified Biotech) and foam caps to limit evaporation. Flasks were incubated at 225 rpm and 30 °C for 3-4 days. Every 24 h, 400 μL of culture (or abiotic control) was withdrawn from each flask. 50 μL of culture was used for OD_{600nm} measurement (**Figure S8**), and the remaining volume was centrifuged at 6000 *g* for 1 min and supernatants were passed through 0.2 μm filters for metabolite analysis. An additional 5 mM glucose was added to each biotic flask every 24 h to support growth and promote metabolite turnover.

<u>**Synthetic Methods for Standards and Model Compounds**</u>

Representative acetylation procedure of phenolic compounds: Pyridine (10 mL/g phenol) and acetic anhydride (10 mL/g phenol) were added to the phenolic starting material. The mixture was stirred at 40 °C overnight, the volatiles were subsequently removed *in vacuo.* The resulting mixture was washed with saturated aq. $NaHCO_3$ and extracted with EtOAc. The organic fraction was washed with H_2O, dried over Na_2SO_4, and then passed through a silica plug. The filtrate was concentrated to dryness to yield the acetyl-derivatized product. The products were used as-is unless otherwise specified.

Acetyl propenylsyringol

4-propenylsyringol[1] (51.5 mg, 0.27 mmol) was dissolved in acetic anhydride (0.7 mL) and pyridine (0.7 mL). After stirring for 12 hours, ethanol (20 mL) was added to quench the reaction. Acetyl reagents in the reaction mixture were removed by azeotrope with ethanol to obtain an oil. The crude material was purified by preparative TLC (EtOAc:hexane 1:6) to yield the title compound as a colorless solid (34.4 mg, 55 % yield). ^{1}H NMR (400 MHz,

$CDCl_3$): δ 6.57 (s, 2H), 6.34 (dd, *J* = 15.7, 1.6 Hz, 1H), 6.19 (dq, *J* = 15.6, 6.6 Hz, 1H), 3.82 (s, 6H), 2.33 (s, 3H), 1.88 (dd, *J* = 6.6, 1.6 Hz, 3H). ^{13}C NMR (75 MHz, $CDCl_3$): δ 168.89, 152.07, 136.46, 130.86, 127.63, 126.21, 102.49, 56.06, 20.49, 18.39. GC-EIMS (m/z): Calcd. 236.1 ($[M]^+$); Found. 236.1

Acetyl β-5 dimer

The phenolic analogue (45.6 mg, 0.14 mmol)[3] was acetylated in the same manner as acetyl propenylsyringol, and the crude material was purified by preparative TLC (EtOAc:hexane 1:4) to yield the title compound as a colorless oil (44.0 mg, 76.9 % yield). ^1H NMR (400 MHz, $CDCl_3$): δ 6.90 (d, *J* = 8.0 Hz, 1H), 6.69 (dd, *J* = 8.0, 1.8 Hz, 1H), 6.65 (d, *J* = 1.7 Hz, 1H), 6.63 (d, *J* = 1.8 Hz, 1H), 6.62 (d, *J* = 1.8 Hz, 1H), 3.79 (s, 3H), 3.74 (s, 3H), 3.15-3.06 (m, 1H), 2.90 (dd, *J* = 13.5, 5.6 Hz, 1H), 2.66 (dd, *J* = 13.5, 8.7 Hz, 1H), 2.54 (t, *J* = 7.5 Hz, 2H), 2.32 (s, 3H), 2.29 (s, 3H), 1.62 (sex, *J* = 7.4 Hz, 2H), 1.18 (d, *J* = 6.9 Hz, 3H), 0.94 (t, *J* = 7.4 Hz, 3H). ^{13}C NMR (75 MHz, $CDCl_3$): δ 169.18, 169.11, 150.64, 150.53, 141.05, 139.59, 139.07, 137.84, 135.40, 122.21, 121.20, 118.67, 113.31, 110.04, 55.88, 55.69, 43.36, 38.31, 34.84, 24.62, 20.69, 20.58, 19.99, 13.91.

Acetyl propiovanillone

Acetylation of propiovanillone (0.20 g, 1.1 mmol) afforded the title compound as a white solid (0.22 g, 90% yield). ^1H NMR (300 MHz, $CDCl_3$) δ 7.60 (d, *J* = 1.9 Hz, 1H), 7.55 (dd, *J* = 8.2, 1.9 Hz, 1H), 7.10 (d, *J* = 8.1 Hz, 1H), 3.88 (s, 3H), 2.98 (q, *J* = 7.2 Hz, 2H), 2.32 (s, 3H), 1.21 (t, *J* = 7.2 Hz, 3H). ^{13}C NMR (75 MHz, $CDCl_3$) δ 199.71, 168.63, 151.48, 143.71, 135.84, 122.85, 121.39, 111.55, 56.12, 31.81, 20.75, 8.42. GC-EIMS (m/z): Calcd. 222.1 ($[M]^+$); Found. 222.1.

Acetyl propiosyringone

Acetylation of propiosyringone (40 mg, 0.19 mmol) afforded the title compound as an off-white solid (33 mg, 68% yield). ^1H NMR (300 MHz, $CDCl_3$) δ 7.23 (s, 2H), 3.88 (s, 6H), 2.98 (q, *J* = 7.2 Hz, 2H), 2.35 (s, 3H), 1.23 (t, *J* = 7.2 Hz, 3H). ^{13}C NMR (75 MHz, $CDCl_3$) δ 199.63, 168.37, 152.37, 135.11, 132.80, 104.93, 56.45, 31.84, 20.58, 8.45. GC-EIMS (m/z): Calcd. 252.1 ($[M]^+$); Found. 252.1.

Diacetyl α-guaiacylpropanol

Acetylation of α-guaiacylpropanol (0.10 g, 0.55 mmol) afforded the title compound as a pale yellow oil (0.11 g, 77% yield). ^1H NMR (300 MHz, $CDCl_3$) δ 7.03 – 6.97 (m, 1H), 6.93 – 6.88 (m, 2H), 5.68 – 5.61 (m, 1H), 3.84 (s, 3H), 2.30 (s, 3H), 2.08 (s, 3H), 2.00 – 1.71 (m, 2H), 0.89 (t, *J* = 7.4 Hz, 3H). ^{13}C NMR (75 MHz, $CDCl_3$) δ 170.58, 169.24, 151.19, 139.66, 139.49, 122.85, 119.13, 111.12, 77.19, 56.13, 29.54, 21.50, 20.93, 10.25. GC-EIMS (m/z): Calcd. 266.1 ($[M]^+$); Found. 266.1.

Diacetyl α-hydroxypropiovanillone

Acetylation of α-hydroxypropiovanillone (0.10 g, 0.51 mmol) afforded the title compound as an off-white solid (0.10 g, 70% yield). ^1H NMR (300 MHz, $CDCl_3$) δ 7.58 (d, *J* = 1.9 Hz, 1H), 7.54 (dd, *J* = 8.2, 1.9 Hz, 1H), 7.13 (d, *J* = 8.2 Hz, 1H), 5.93 (q, *J* = 7.0 Hz, 1H), 3.88 (s, 3H), 2.33 (s, 3H), 2.14 (s, 3H), 1.53 (d, *J* = 7.0 Hz, 3H). ^{13}C NMR (75 MHz, $CDCl_3$) δ 195.82, 170.54, 168.52, 151.80, 144.35, 133.17, 123.11, 121.70, 112.24, 71.35, 56.20, 20.86, 20.77, 17.35. GC-EIMS (m/z): Calcd. 280.1 ($[M]^+$); Found. 280.1

Diacetyl pinoresinol

Acetylation of pinoresinol (0.18 g, 0.50 mmol) was carried out, and the concentrated crude reaction mixture washed with saturated aq. $NaHCO_3$ (10 mL) and extracted with THF (10 mL). The organic fraction was washed with H_2O (10 mL), dried over Na_2SO_4, and then passed through a silica plug. The solution was concentrated to dryness, and the resulting solid was washed with hexanes (10 mL x 2) and Et_2O (10 mL x 2) to afford the title compound as a white solid. (0.14 g, 63% yield). ^1H NMR (300 MHz, $CDCl_3$) δ 7.01 (d, *J* = 8.1 Hz, 2H), 6.99 (d, *J* = 1.8 Hz, 2H), 6.89 (ddd, *J* = 8.1, 1.9, 0.7 Hz, 2H), 4.80 (d, *J* = 4.2 Hz, 2H), 4.28 (dd, *J* = 9.2, 6.8 Hz, 2H), 3.94 (dd, *J* = 9.3, 3.6 Hz, 2H), 3.85 (s, 6H), 3.15 – 3.06 (m, 2H), 2.31 (s, 6H). ^{13}C NMR (75 MHz, $CDCl_3$) δ 169.23, 151.36, 140.21, 139.24, 122.89, 118.08, 109.99, 85.65, 72.09, 56.06, 54.48, 20.80.

Acetyl ethylsyringol

Acetylation of ethylsyringol (0.50 g, 2.7 mmol) afforded the title compound as a beige solid (0.50 g, 81% yield). ^1H NMR (300 MHz, $CDCl_3$) δ 6.45 (s, 2H), 3.81 (s, 6H), 2.62 (q, *J* = 7.6 Hz, 2H), 2.33 (s, 3H), 1.25 (t, *J* = 7.6 Hz, 3H). ^{13}C NMR (75 MHz, $CDCl_3$) δ 169.11, 151.95, 142.85, 126.66, 104.53, 56.16, 29.54, 20.59, 15.59. GC-EIMS (m/z): Calcd. 224.1 ($[M]^+$); Found. 224.1.

Acetyl methyl vanillate

Acetylation of methyl vanillate (1.0 g, 5.5 mmol) afforded the title compound as a colorless solid (0.80 g, 65% yield). ^{1}H NMR (300 MHz, $CDCl_3$) δ 7.70 – 7.62 (m, 2H), 7.08 (dd, *J* = 7.4, 0.9 Hz, 1H), 3.90 (s, 6H), 3.87 (s, 3H), 2.31 (s, 3H). ^{13}C NMR (75 MHz, $CDCl_3$) δ 168.54, 166.42, 151.13, 143.69, 128.90, 122.86, 122.67, 113.48, 56.13, 52.35, 20.71. GC-EIMS (m/z): Calcd. 224.1 ($[M]^+$); Found. 224.1.

Acetyl methyl syringate

Acetylation of methyl syringate (0.50 g, 2.4 mmol) afforded the title compound as a white solid (0.46 g, 77% yield). ^{1}H NMR (300 MHz, $CDCl_3$) δ 7.32 (s, 2H), 3.91 (s, 3H), 3.86 (s, 6H), 2.34 (s, 3H). ^{13}C NMR (75 MHz, $CDCl_3$) δ 168.29, 166.49, 152.17, 132.66, 128.18, 106.39, 56.40, 52.46, 20.52. GC-EIMS (m/z): Calcd. 254.1 ($[M]^+$); Found. 254.1.

Acetyl carboxyvanillic acid

Acetic anhydride (5 mL) was added to 5-carboxyvanillic acid (0.50 g, 2.4 mmol), and the mixture was stirred at 50 °C overnight. The volatiles were then removed under vacuum, and H_2O (1 mL) and EtOAc (5 mL) were added to the resulting colorless solid. The reaction mixture was stirred at 25 °C for 1 d, and the resulting homogeneous solution was concentrated to dryness. The resulting colorless solid was dissolved in EtOAc (20 mL), passed through a silica plug, and then concentrated. The resulting white solid was washed with minimal H_2O (0.3 mL) and Et_2O (5 mL) and then concentrated to dryness to yield the title compound as a colorless solid (0.13 g, 21% yield). ^{1}H NMR (300 MHz, DMSO) δ 13.36 (bs, 2H), 8.04 (d, *J* = 1.9 Hz, 1H), 7.78 (d, *J* = 2.0 Hz, 1H), 3.88 (s, 3H), 2.27 (s, 3H). ^{13}C NMR (75 MHz, DMSO) δ 168.16, 166.08, 164.96, 151.99, 142.75, 128.88, 125.27, 123.37, 116.25, 56.40, 20.53. LC-ESI (negative mode, m/z): Calcd. 253 ($[M]^+$−H); Found. 253. See **Table S16** below.

Dimethyl carboxyvanillate

A mixture of H_2SO_4 (50 µL, 1.0 µmol) and 5-carboxyvanillic acid (0.50 g, 2.4 mmol) in MeOH (5 mL) was stirred at 65 °C for 1 d, yielding a colorless, homogeneous solution. The reaction mixture was concentrated to dryness, and the resulting solids were washed with a saturated aq. $NaHCO_3$ solution (20 mL) and extracted with EtOAc (10 mL x 3). The organic fraction was dried over Na_2SO_4, passed through a silica plug, and then concentrated to dryness to yield the title compound as a colorless solid (0.33 g, 58% yield). ^{1}H NMR (300 MHz, $CDCl_3$) δ 11.44 (s, 1H), 8.17 (d, *J* = 2.0 Hz, 1H), 7.65 (d, *J* = 1.9 Hz, 1H), 3.97 (s, 3H), 3.94 (s, 3H), 3.90 (s, 3H). ^{13}C NMR (75 MHz, $CDCl_3$) δ 170.54, 166.24, 155.90, 148.60, 123.89, 120.85, 116.41, 112.15, 56.43, 52.81, 52.31. GC-EIMS (m/z): Calcd. 240.1 ($[M]^+$); Found. 240.1.

Acetyl dimethyl 5-carboxyvanillate

Acetylation of dimethyl 5-carboxyvanillate (0.15 g, 0.6 mmol) afforded the title compound as an off-white solid (0.15 g, 86% yield). ^{1}H NMR (300 MHz, $CDCl_3$) δ 8.23 (d, *J* = 1.9 Hz, 1H), 7.78 (d, *J* = 1.9 Hz, 1H), 3.93 (s, 3H), 3.90 (s, 3H), 3.88 (s, 3H), 2.37 (s, 3H). ^{13}C NMR (75 MHz, $CDCl_3$) δ 168.58, 165.77, 164.27, 152.41, 143.95, 128.27, 124.52, 124.38, 116.66, 56.59, 52.62, 52.54, 20.73. GC-EIMS (m/z): Calcd. 282.1 ($[M]^+$); Found. 282.1.

Diacetyl dehydrodivanillin

Acetylation of dehydrodivanillin (0.50 g, 1.7 mmol) afforded crude product. The material was subsequently washed with hexanes (5 mL x 2) and dried under vacuum to afford the title compound as an off-white solid (0.26 g, 41% yield). ^{1}H and ^{13}C NMR data match previously reported data on this compound.[6]

Diacetyl dehydrodivanillic acid (acetyl vanillic acid dimer)

Pyridine (2 mL) and acetic anhydride (2 mL) were added to dehydrodivanillic acid (0.50 g, 1.5 mmol) and the mixture was stirred at 40 °C overnight. The resulting brown mixture was concentrated under reduced pressure, and H_2O (5 mL) and Et_2O (5 mL) were added the resulting brown oil. The mixture was stirred at 40 °C for 1 d, which resulted in the formation of ample off-white precipitate. The solids were collected by vacuum filtration and washed with H_2O (10 mL x 2) and Et_2O (10 mL x 2) to afford the title compound as an off-white solid (0.26 g, 41% yield). ^{1}H NMR (300 MHz, DMSO-d_6) δ 13.21 (bs, 2H), 7.67 (d, *J* = 1.9 Hz, 2H), 7.40 (d, *J* = 1.8 Hz, 2H), 3.88 (s, 6H), 2.06 (s, 6H). ^{13}C NMR (75 MHz, DMSO-d_6) δ 167.70, 166.36, 151.25, 140.54, 130.13, 129.14, 123.13, 112.95, 56.25, 20.03. LC-ESI (negative mode, m/z): Calcd. 417 ($[M]^+$−H); Found. 417. See **Table S16** below.

Acetyl acetovanillone

Acetylation of acetovanillone (0.5 g, 3.0 mmol) afforded the title compound as a yellow oil (0.5 g, 81% yield). ^{1}H NMR (300 MHz, $CDCl_3$) δ 7.59 (d, *J* = 1.9 Hz, 1H), 7.54 (dd, *J* = 8.1, 1.9 Hz, 1H), 7.11 (d, *J* = 8.1 Hz, 1H), 3.88 (s, 3H), 2.58 (s, 3H), 2.33 (s, 3H). ^{13}C NMR (75 MHz, $CDCl_3$) δ 197.01, 168.60, 151.50, 143.93, 136.05, 122.90, 122.06, 111.55, 56.14, 26.64, 20.75. GC-EIMS (m/z): Calcd. 208.1 ($[M]^+$); Found. 208.1.

Acetyl acetosyringone

Acetylation of acetosyringone (0.3 g, 1.5 mmol) afforded the title compound as a colorless solid (0.31 g, 85% yield). ^{1}H NMR (300 MHz, $CDCl_3$) δ 7.21 (s, 2H), 3.87 (s, 6H), 2.58 (s, 3H), 2.34 (s, 3H). ^{13}C NMR (75 MHz, $CDCl_3$) δ 196.89, 168.29, 152.33, 135.24, 132.94, 105.21, 56.41, 26.62, 20.53. GC-EIMS (m/z): Calcd. 238.1 ($[M]^+$); Found. 238.1.

Diacetyl dimethoxyhydroquinone

Acetylation of 2,6-dimethoxyhydroquinone (0.5 g, 2.9 mmol) afforded crude product. The solid mixture was washed with hexanes (3 mL) and then recrystallized from saturated Et_2O solution at 25 °C to afford the title compound as a tan solid (0.18 g, 24% yield). ^{1}H and ^{13}C NMR data match previously reported data on this compound.[7]

Acetyl methylguaiacol

Acetylation of methylguaiacol (0.50 g, 3.6 mmol) afforded the title compound as a pale yellow oil (0.47 g, 71% yield). ^{1}H NMR (300 MHz, $CDCl_3$) δ 6.92 (d, *J* = 8.0 Hz, 1H), 6.79 (d, *J* = 1.9 Hz, 1H), 6.75 (ddd, *J* = 8.0, 1.9, 0.8 Hz, 1H), 3.82 (s, 3H), 2.35 (s, 3H), 2.31 (s, 3H).^{13}C NMR (75 MHz, $CDCl_3$) δ 169.38, 150.74, 137.55, 136.95, 122.44, 121.23, 113.36, 55.85, 21.51, 20.75. GC-EIMS (m/z): Calcd. 180.1 ($[M]^+$); Found. 180.1.

Acetyl ethylguaiacol

Acetylation of ethylguaiacol (0.50 g, 3.3 mmol) afforded the title compound as a pale yellow oil (0.49 g, 77% yield). ^{1}H NMR (300 MHz, $CDCl_3$) δ 6.94 (d, *J* = 8.0 Hz, 1H), 6.81 (d, *J* = 1.9 Hz, 1H), 6.78 (dd, *J* = 8.0, 1.9 Hz, 1H), 3.83 (s, 3H), 2.65 (q, *J* = 7.6 Hz, 2H), 2.31 (s, 3H), 1.25 (t, *J* = 7.6 Hz, 3H). ^{13}C NMR (75 MHz, $CDCl_3$) δ 169.38, 150.83, 143.27, 137.69, 122.47, 119.95, 112.20, 55.86, 28.90, 20.76, 15.59. GC-EIMS (m/z): Calcd. 194.1 ($[M]^+$); Found. 194.1.

Acetyl propylguaiacol

Acetylation of propylguaiacol (0.50 g, 3.0 mmol) afforded the title compound as a colorless oil (0.45 g, 71% yield). ^{1}H NMR (300 MHz, $CDCl_3$) δ 6.93 (d, *J* = 8.0 Hz, 1H), 6.79 (d, *J* = 1.9 Hz, 1H), 6.75 (dd, *J* = 7.9, 1.9 Hz, 1H), 3.82 (s, 3H), 2.58 (t, *J* = 8.7, 7.5 Hz, 2H), 2.31 (s, 3H), 1.65 (h, *J* = 7.4 Hz, 2H), 0.96 (t, *J* = 7.3 Hz, 3H). ^{13}C NMR (75 MHz, $CDCl_3$) δ 169.36, 150.77, 141.77, 137.73, 122.38, 120.60, 112.72, 55.87, 38.12, 24.62, 20.78, 13.96. GC-EIMS (m/z): Calcd. 208.1 ($[M]^+$); Found. 208.1.

Acetyl propylsyringol

Acetylation of propylsyringol (0.40 g, 2.0 mmol) afforded the title compound as an off-white solid (0.43 g, 88% yield). ^{1}H NMR data matches previously reported data on this compound.[8] ^{13}C NMR (75 MHz, $CDCl_3$) δ 169.05, 151.85, 141.31, 126.67, 105.06, 56.12, 38.76, 24.61, 20.56, 13.99. GC-EIMS (m/z): Calcd. 238.1 ($[M]^+$); Found. 238.1.

Diacetyl propanolguaiacol

Acetylation of 4-propanolguaiacol (0.40 g, 2.2 mmol) afforded the title compound as a pale yellow oil (0.49 g, 98% yield). ^{1}H NMR (300 MHz, $CDCl_3$) δ 6.93 (d, *J* = 7.9 Hz, 1H), 6.78 (d, *J* = 1.9 Hz, 1H), 6.75 (dd, *J* = 8.0, 2.0 Hz, 1H), 4.10 (t, *J* = 6.5 Hz, 2H), 3.81 (s, 3H), 2.73 – 2.62 (m, 2H), 2.29 (s, 3H), 2.05 (s, 3H), 2.01 – 1.89 (m, 2H). ^{13}C NMR (75 MHz, $CDCl_3$) δ 171.22, 169.30, 150.97, 140.30, 138.03, 122.66, 120.54, 112.65, 63.88, 55.92, 32.27, 30.25, 21.06, 20.77. GC-EIMS (m/z): Calcd. 266.1 ($[M]^+$); Found. 266.1.

Diacetyl propanolsyringol

Acetylation of propanolsyringol (0.25 g, 1.2 mmol) afforded the title compound as a tan solid (0.26 g, 75% yield). ^{1}H NMR (300 MHz, $CDCl_3$) δ 6.42 (s, 2H), 4.11 (t, *J* = 6.5 Hz, 2H), 3.80 (s, 6H), 2.70 – 2.58 (m, 2H), 2.32 (s, 3H), 2.05 (s, 3H), 2.02 – 1.89 (m, 2H). ^{13}C NMR (75 MHz, $CDCl_3$) δ 171.23, 169.01, 152.06, 139.87, 126.96, 105.03, 63.92, 56.19, 32.92, 30.27, 21.07, 20.57. GC-EIMS (m/z): Calcd. 296.1 ($[M]^+$); Found. 296.1.

Diacetyl tetrahydrodieugenol

Acetylation of tetrahydrodieugenol (0.50 g, 1.5 mmol) afforded crude product. The material was subsequently washed with hexanes (5 mL x 2) and dried under vacuum to afford the title compound as a colorless solid (0.41 g, 65% yield). ^{1}H NMR (300 MHz, DMSO-d_6) δ 6.97 (d, J = 1.9 Hz, 2H), 6.55 (d, J = 1.9 Hz, 2H), 3.78 (s, 6H), 2.55 (t, J = 7.6 Hz, 4H), 2.00 (s, 6H), 1.61 (h, J = 7.5 Hz, 4H), 0.91 (t, J = 7.3 Hz, 6H). ^{13}C NMR (75 MHz, DMSO-d_6) δ 168.18, 150.80, 140.19, 134.91, 130.41, 121.41, 112.22, 55.84, 37.09, 24.03, 20.10, 13.63. GC-EIMS (m/z): Calcd. 414.2 ([M]$^+$); Found. 414.2.

Diacetyl diguaiacylethane

Acetylation of 1,2-diguaiacylethane (0.50 g, 1.8 mmol) afforded crude product. The material was subsequently washed with hexanes (20 mL x 2) and dried under vacuum to afford the title compound as a colorless solid (0.58 g, 88% yield). ^{1}H NMR (300 MHz, $CDCl_3$) δ 6.94 (d, J = 8.0 Hz, 2H), 6.76 (dd, J = 8.0, 1.9 Hz, 2H), 6.68 (d, J = 1.9 Hz, 2H), 3.77 (s, 6H), 2.90 (s, 4H), 2.30 (s, 6H). ^{13}C NMR (75 MHz, $CDCl_3$) δ 169.31, 150.83, 140.52, 138.04, 122.61, 120.65, 112.98, 55.93, 37.92, 20.79. GC-EIMS (m/z): Calcd. 358.1 ([M]$^+$); Found. 358.1.

Preparation of RCF Substrates

Production of extractives-free poplar: Poplar wood chips were extracted with a 50:50 vol% mixture of acetone (Fisher Scientific A18) and *n*-hexanes (Thermo-Fisher Scientific 232100010) in a large Soxhlet extractor. The extraction procedure was adapted from a large-scale method from the NREL Laboratory Analytical Procedure "Determination of Extractives in Biomass".[4] A large volume Soxhlet extractor (Custom, Allen Scientific Glassware, Boulder Colorado) with a 5 L round bottom flask (Chemglass CG-1524-22) for solvent was used in place of the 85 mL capacity Soxhlet and 500 mL solvent reception flask described in the procedure. Total sample mass was 352 g with a solvent volume of approximately 3 L. Extractions were performed under reflux conditions at 50 °C overnight, and 335.3 g of extractive-free biomass was isolated. Total extractives content was calculated by drying an aliquot of the refluxed solvent and are an average of two aliquots (0.6 wt% extractives removed from the poplar chips).

Production of poplar RCF oil: A 7.6 L Parr reactor was loaded with 5 wt% Ru/C (15 g), nanopure DI water (15 g, to wet the catalyst), extractives-free poplar (300 g), and methanol (3 L), and the reactor was subsequently sealed. A leak test of the reactor was conducted at 1700 psig with N_2, followed by 2 purge cycles with N_2 at 500 psig. The system was then pressurized with H_2 to 435 psig at room temperature. The reactor was heated at 225 °C for 3 h with mechanical stirring. Afterwards, the reactor was cooled to room temperature with chilled water through an internal loop. The reaction mixture was passed through a frit to separate the catalyst and remaining biomass, and the filtrate was collected using a peristaltic pump. Methanol was removed from the filtrate by rotary evaporation at 80 mbar with heating to 35 °C. The resulting oil was washed with H_2O (*ca.* 400 mL) and extracted with EtOAc (*ca.* 400 mL, then *ca*. 120 mL x 3). The combined organic fraction was dried over Na_2SO_4 and concentrated under vacuum to yield the isolated RCF oil as a brown oil (60.99 g).

Acetylation of poplar RCF oil: Pyridine (50 mL) and acetic anhydride (50 mL) were added to a round bottom containing poplar RCF oil (10.0 g), and the reaction mixture was stirred for 24 h at 40 °C under an N_2 atmosphere. The volatiles were subsequently removed *in vacuo* to yield a brown oil, which extracted with EtOAc (100 mL x 2) and sequentially washed with saturated aq. $NaHCO_3$ (100 mL), H_2O (100 mL), and brine (100 mL). The organic fraction was dried over Na_2SO_4 and then filtered. The filtrate was concentrated to dryness to yield the acetylated RCF oil as a brown oil (10.1 g).

Vacuum distillation of acetyl poplar RCF oil: Acetylated RCF oil (9.00 g) was transferred to a round bottom attached to a short-path distillation head, and the starting vessel was heated in a sand bath to *ca*. 250 °C under vacuum (*ca.* 50 mbar) until the volatiles were distilled off. The isolated distillate was a pale yellow oil (acetyl monomer fraction, 4.57 g), and the starting vessel contained the remaining brown solid (acetyl oligomer fraction, 3.56 g).

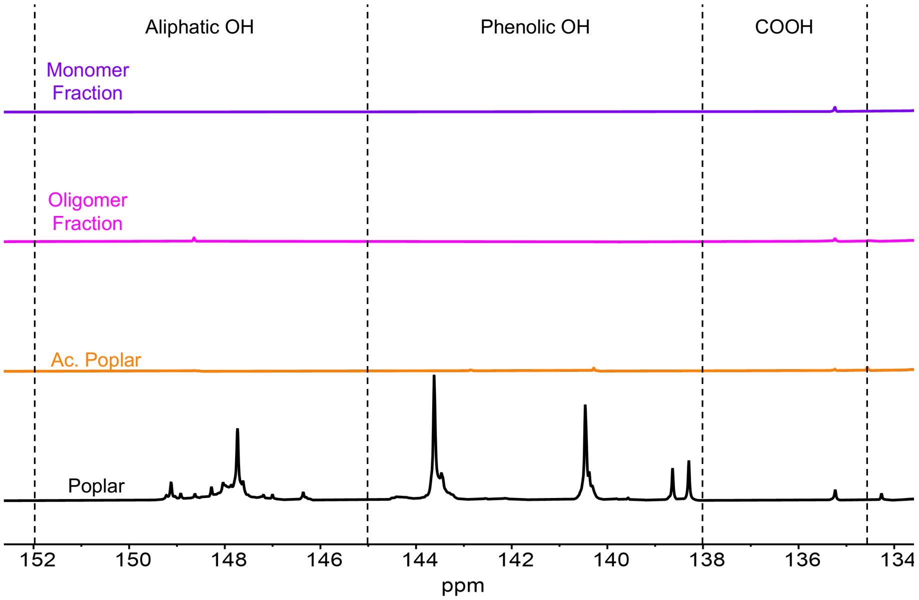

Figure S1. ^{31}P NMR spectra of poplar RCF oil (black), acetylated poplar RCF oil (orange), acetylated oligomer fraction (pink), and the distillate monomer fraction (purple).

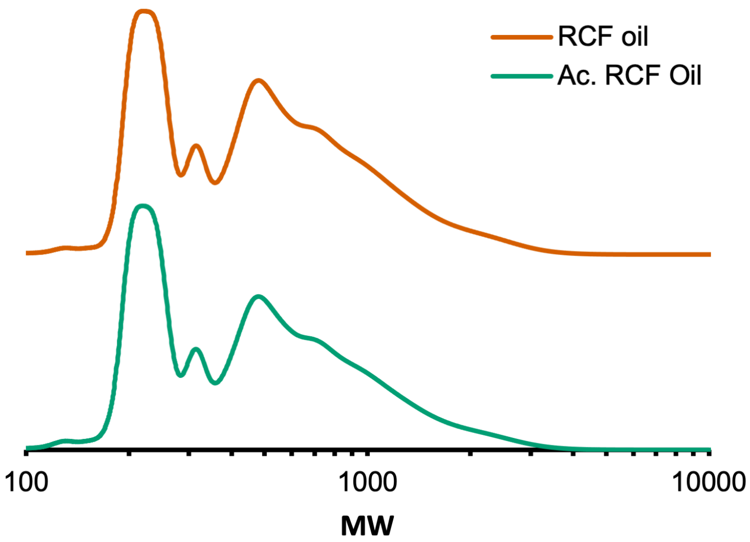

Figure S2. GPC traces of RCF oil and acetyl RCF oil.

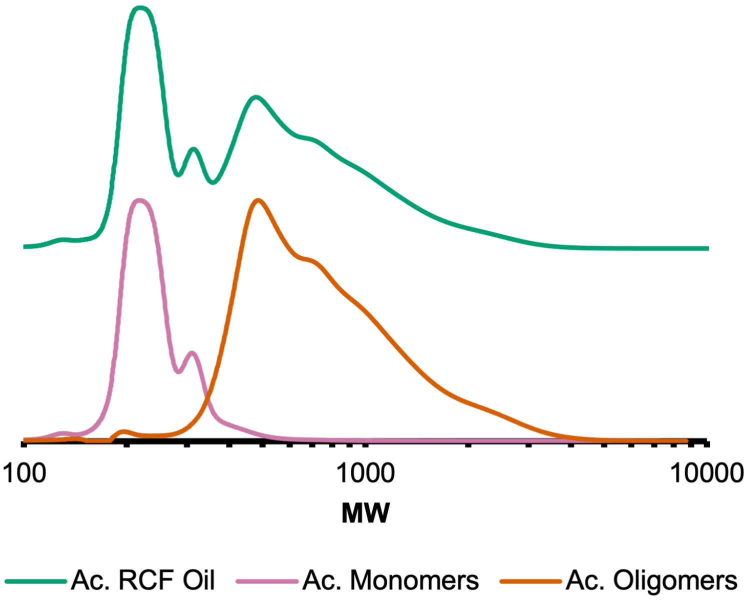

Figure S3. GPC traces of acetyl RCF oil and the acetyl monomer and oligomer fractions.

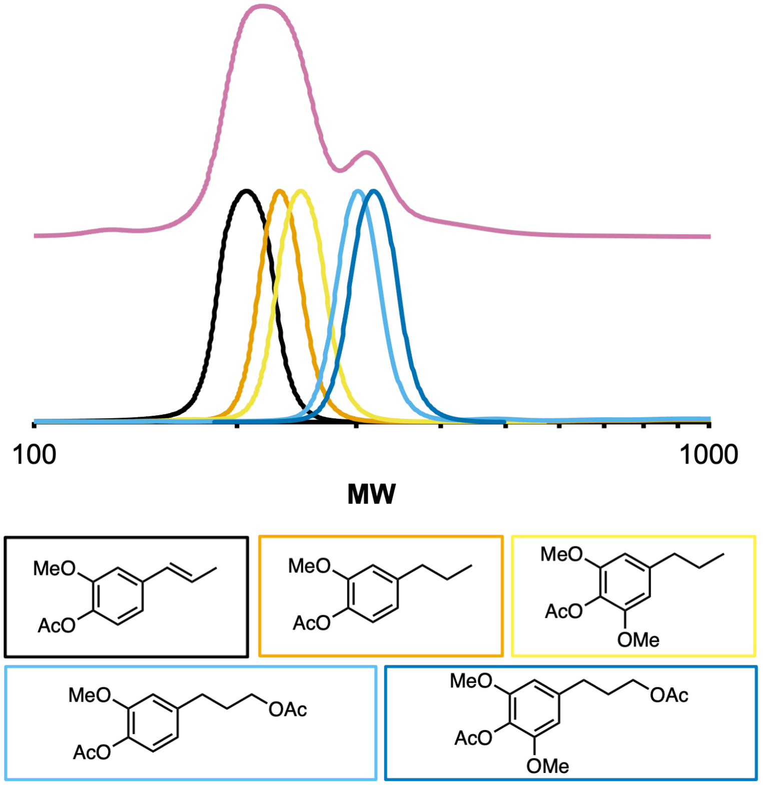

Figure S4. GPC traces of acetyl RCF monomer fraction (top, pink) and acetyl RCF compounds (bottom).

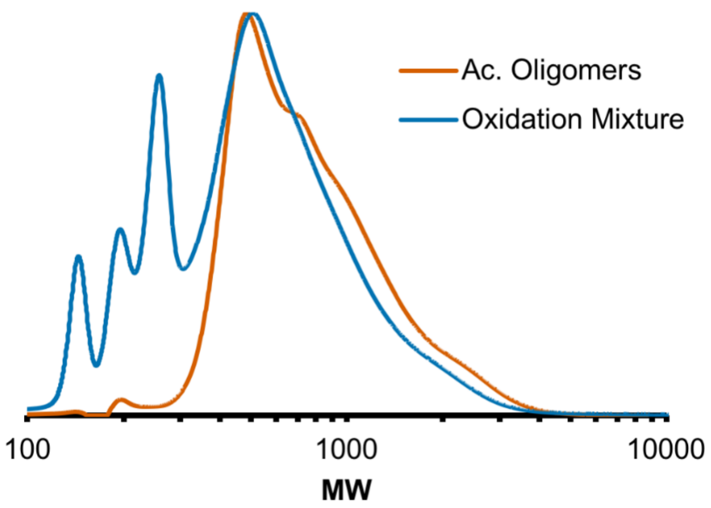

Figure S5. GPC traces of the acetyl oligomer fraction (orange) and oxidation mixture (blue).

Table S1. Determination of EtOAc content in RCF oil substrates.

Sample	Sample Mass (g)	Int Std Mass (g)	mol EtOAc/ mol Int Std	EtOAc mass (g)	wt% EtOAc
RCF Oil	0.0211	0.002	5.94	0.0041	19.7%
Acetyl RCF Oil	0.0206	0.002	2.53	0.0018	8.6%

Wt% calculation for acetyl monomer and acetyl oligomer fractions: The acetyl RCF oil contains 8.6 wt% EtOAc, and ^{1}H NMR analysis of the acetyl monomer and acetyl oligomer fractions following distillation show the EtOAc is removed during vacuum distillation (see Figures S14-15). Thus, the wt% calculations for the acetyl monomer and acetyl oligomer fractions use a EtOAc-corrected mass for the initial acetyl RCF oil as follows:

$$EtOAc\ corrected\ RCF\ oil\ mass = RCF\ oil\ mass * (1 - wt\%\ of\ EtOAc)$$

Table S2. Mass of acetyl RCF oil corrected for EtOAc content.

Acetyl RCF oil mass (g)	8.9977
EtOAc-corrected acetyl RCF oil mass (g)	8.2256

Table S3. GC-FID quantification of poplar RCF oil.

	mmol / g EtOAc-corrected poplar RCF oil						
Substrate	4-Ethyl Guaiacol	4-Propyl Guaiacol	Isoeugenol	4-Propyl Syringol	4-Propanol Guaiacol	Isoelemicin	4-Propanol Syringol
Poplar RCF Oil	0.005	0.484	0.119	0.590	0.244	0.113	0.255

Table S4. Mass fractions of distilled acetylated RCF oil.

Sample	Mass (g)	wt% of EtOAc-corrected RCF oil
Acetyl Oligomers	3.5601	43.3%
Acetyl Monomers	4.5701	55.6%

Table S5. GC-FID quantification acetyl RCF substrates.

	mmol monomer/g EtOAc-corrected acetyl RCF oil					
	Acetyl Methylguaiacol	Acetyl Ethylguaiacol	Acetyl Propylguaiacol	Acetyl Isoeugenol	Acetyl Propylsyringol	Acetyl Propenylsyringol
Acetyl RCF Oil[1]	0.012	0.025	0.390	0.074	0.480	0.064
Acetyl Monomer[1]	0.009	0.018	0.381	0.070	0.465	0.062
Acetyl Oligomer	0.000	0.000	0.000	0.000	0.000	0.000

	Diacetyl Propanolguaiacol	Diacetyl Propanolsyringol	total mmol analyte/ g EtOAc-corrected acetyl RCF oil	monomer recovery (mol%)
Acetyl RCF Oil[1]	0.171	0.185	1.401	-
Acetyl Monomer[1]	0.163	0.168	1.335	95.3%
Acetyl Oligomer	0.002	0.004	0.006	0.4%

[1]monomer quantification is an average of 3 runs

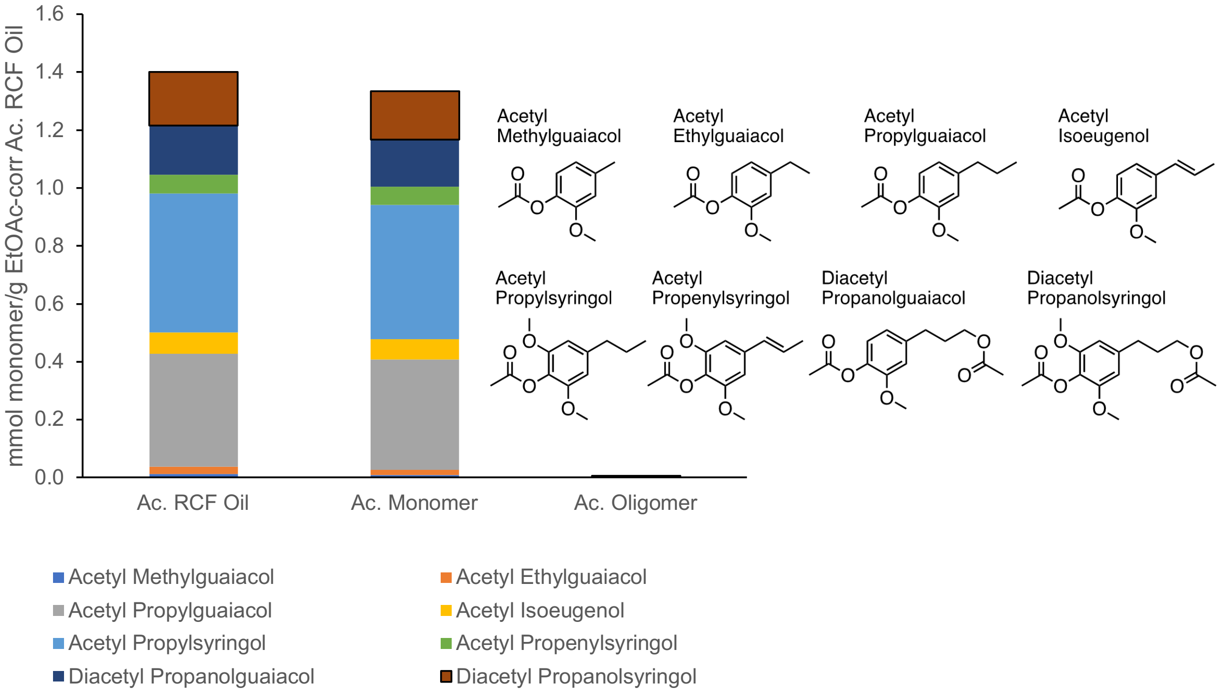

Figure S6. GC-FID quantification acetyl RCF substrates.

Table S6A. Yield from the product recovery experiments.

		mol% yield			
		Acetyl Vanillin	Acetyl Vanillic Acid	Acetyl Syringaldehyde	Acetyl Syringic Acid
Acetyl Vanillin	1	0.4	76	0	0
	2	0.3	71	0	0
	3	0.5	76	0	0
	**Average**	**0.4**	**74**	**0**	**0**
Acetyl Vanillic Acid	1	0	74	0	0
	2	0	87	0	0
	3	0	78	0	0
	**Average**	**0**	**79**	**0**	**0**
Acetyl Syringealdehyde	1	0	0	0.1	56
	2	0	0	0.2	65
	3	0	0	0.3	69
	**Average**	**0**	**0**	**0.2**	**63**
Acetyl Syringic Acid	1	0	0	0	100
	2	0	0	0	89
	3	0	0	0	84
	**Average**	**0**	**0**	**0**	**91**

Table S6B. Yield from single-run product recovery experiments.

	mol% yield								
	Acetyl Vanillin[a]	Acetyl Vanillic Acid[a]	Acetyl Syringaldehyde[a]	Acetyl Syringic Acid[a]	Methoxymaleic Acid[b]	Dimethoxybenzoquinone[b]	Vanillic Acid[b]	Syringic Acid[b]	**Total**
Acetyl Vanillin	0.4	73	0	0	1	0	2		**77**
Acetyl Vanillic Acid	0	71	0	0	0	0	20		**91**
Acetyl Syringealdehyde	0	0	0.3	63	2	9	0	0.2	**74**
Acetyl Syringic Acid	0	0	0	95	0.1	3	0	3	**101**

[a]quantified by LC-MS [b]quantified by UHPLC

RCF Oil Oxidation Quantification Data

Table S7. Numerical data to generate Figure 3A.

**Co mmol**	Acetyl Vanillin	Acetyl Vanillic Acid	Acetyl Syringaldehyde	Acetyl Syringic Acid	Acetyl Carboxyvanillic Acid	Acetyl Acetovanillone	Acetyl Acetosyringone
	**mmol analyte/g Ac. Oligomer**						
0.001	0.0451	0.0248	0.0574	0.0293	0	0.0032	0.0036
0.003[a]	0.0674	0.0949	0.0900	0.1853	0	0.0064	0.0087
0.005	0.0761	0.0911	0.0991	0.1583	0	0.0106	0.0165
0.007	0.0422	0.1249	0.0461	0.2735	0	0.0032	0.0037
0.009	0.0603	0.1354	0.0744	0.2470	0	0.0109	0.0173

Acetyl Propiovanillone	Acetyl Propio-syringone	Diacetyl Dimethoxy-hydroquinone	Vanillin	Vanillic Acid	Syringaldehyde	Syringic Acid	Carboxy Vanillic Acid
0.0022	0.0037	0	0	0	0	0	0
0.0034	0.0058	0.0009	0.0031	0	0.0130	0.0085	0.0025
0.0054	0.0104	0.0031	0	0	0	0	0
0.0029	0.0044	0.0061	0.0022	0.0551	0.0162	0.0730	0.0029
0.0047	0.0090	0.0000	0	0	0	0	0

Dimethoxybenzoquinone	Methoxymaleic acid	Vanillic Acid Dimer	Diacetyl Propanolguaiacol	Diacetyl Propanolsyringol	Propylsyringol	Acetyl Vanillic Acid Dimer
0.0241	0.0022	0	0	0.0106	0	0
0.0800	0.0222	0	0	0.0035	0	0.0013
0.0560	0.0170	0	0	0.0038	0	0.0017
0.1286	0.0374	0	0	0	0	0
0.0755	0.0252	0	0	0	0	0.0009

[a] Values for this entry are an average for four runs, see Table S11 for data on individual runs. All other entries are single runs.

Table S8. Numerical data to generate Figure 3B.

**NaBr mmol**	Acetyl Vanillin	Acetyl Vanillic Acid	Acetyl Syringaldehyde	Acetyl Syringic Acid	Acetyl Carboxyvanillic Acid	Acetyl Acetovanillone	Acetyl Acetosyringone
	**mmol analyte/g Ac. Oligomer**						
0.001	0.0573	0.0566	0.0700	0.1022	0	0.0020	0.0000
0.003[a]	0.0674	0.0949	0.0900	0.1853	0	0.0064	0.0087
0.005	0.0715	0.1375	0.0877	0.2378	0	0.0092	0.0141
0.007	0.0641	0.1170	0.0743	0.2091	0	0.0072	0.0096

Acetyl Propiovanillone	Acetyl Propiosyringone	Diacetyl Dimethoxyhydroquinone	Vanillin	Vanillic Acid	Syringaldehyde	Syringic Acid	Carboxyvanillic Acid
0	0.0021	0	0	0	0.0076	0.0031	0
0.0034	0.0058	0.0009	0.0031	0	0.0130	0.0085	0.0025
0.0040	0.0073	0.0000	0.0029	0	0.0089	0.0086	0.0025
0.0036	0.0067	0.0043	0.0038	0.0296	0.0220	0.0253	0.0037

Dimethoxybenzoquinone	Methoxymaleic acid	Vanillic Acid Dimer	Diacetyl Propanolguaiacol	Diacetyl Propanolsyringol	Propylsyringol	Acetyl Vanillic Acid Dimer
0.0671	0.0123	0	0	0.0085	0	0
0.0800	0.0222	0	0	0.0035	0	0.0013
0.0772	0.0244	0	0	0.0011	0	0.0013
0.0799	0.0215	0	0	0	0	0.0008

[a]Values for this entry are an average for four runs, see Table S11 for data on individual runs. All other entries are single runs.

Table S9. Numerical data to generate Figure 3C.

	**mmol analyte/g Ac. Oligomer**						
**Temperature**	Acetyl Vanillin	Acetyl Vanillic Acid	Acetyl Syringaldehyde	Acetyl Syringic Acid	Acetyl Carboxyvanillic Acid	Acetyl Acetovanillone	Acetyl Acetosyringone
100 °C	0.0000	0.0000	0.0189	0.0000			
120 °C[a]	0.0674	0.0949	0.0900	0.1853		0.0064	0.0087
140 °C	0.0295	0.1507	0.0223	0.2391		0.0023	0.0025
160 °C	0.0659	0.0458	0.0984	0.1101		0.0114	0.0201
180 °C	0.0429	0.0582	0.0634	0.1229	0.0000	0.0064	0.0136

Acetyl Propiovanillone	Acetyl Propiosyringone	Diacetyl Dimethoxyhydroquinone	Vanillin	Vanillic Acid	Syringaldehyde	Syringic Acid	Carboxyvanillic Acid
0	0	0	0	0	0.0080	0	0
0.0034	0.0058	0.0009	0.0031	0	0.0130	0.0085	0.0025
0.0028	0.0035	0.0000	0	0	0	0.0023	0
0.0050	0.0106	0.0000	0.0097	0	0.0313	0.0073	0.0066
0	0.0032	0.0000	0.0187	0.0458	0.0655	0.0201	0.0201

Dimethoxy-benzoquinone	Methoxy maleic acid	Vanillic Acid Dimer	Diacetyl Propanol guaiacol	Diacetyl Propanolsyringol	Propylsyringol	Acetyl Vanillic Acid Dimer
0.0036	0	0	0	0.0114	0	0
0.0800	0.0222	0	0	0.0035	0	0.0013
0.0929	0.0835	0	0	0	0	0
0.1753	0.0307	0	0	0.0035	0	0.0021
0.3836	0.0389	0	0	0.0023	0	0.0010

[a]Values for this entry are an average for four runs, see Table S11 for data on individual runs. All other entries are single runs.

Table S10. Numerical data to generate Figure 3D.

mmol analyte/g Ac. Oligomer

**Reaction Time**	Acetyl Vanillin	Acetyl Vanillic Acid	Acetyl Syringaldehyde	Acetyl Syringic Acid	Acetyl Carboxyvanillic Acid	Acetyl Acetovanillone	Acetyl Acetosyringone
0 h	0.0000	0.0000	0.0189	0.0000	0	0	0
1 h	0.0255	0.0000	0.0239	0.0107	0	0	0.0027
2 h[a]	0.0674	0.0949	0.0900	0.1853	0	0.0064	0.0087
3 h	0.0597	0.0681	0.0904	0.1495	0	0.0072	0.0099
4 h	0.0525	0.0339	0.0731	0.0786	0	0.0046	0.0055

Reaction Time	Acetyl Propiovanillone	Acetyl Propiosyringone	Diacetyl Dimethoxyhydroquinone	Vanillin	Vanillic Acid	Syringaldehyde	Syringic Acid	Carboxyvanillic Acid
0 h	0	0	0	0	0	0.0039	0	0
1 h	0	0	0	0	0	0.0059	0	0
2 h[a]	0.0034	0.0058	0.0009	0.0031	0	0.0130	0.0085	0.0025
3 h	0.0035	0.0063	0.0000	0.0046	0	0.0216	0.0136	0.0018
4 h	0.0030	0.0045	0.0000	0.0055	0.0298	0.0326	0.0331	0.0026

Reaction Time	Dimethoxybenzoquinone	Methoxymaleic acid	Vanillic Acid Dimer	Diacetyl Propanolguaiacol	Diacetyl Propanolsyringol	Propylsyringol	Acetyl Vanillic Acid Dimer
0 h	0.0030	0	0	0	0.0112	0	0
1 h	0.0056	0	0	0	0.0118	0	0
2 h[a]	0.0800	0.0222	0	0	0.0035	0	0.0013
3 h	0.0805	0.0210	0	0	0.0045	0	0.0012
4 h	0.1271	0.0199	0	0	0.0038	0	0

[a] Values for this entry are an average for four runs, see Table S11 for data on individual runs. All other entries are single runs.

Table S11. Individual runs for optimized conditions for Figure 4.

	mmol analyte/g Ac. Oligomer						
**Run**	Acetyl Vanillin	Acetyl Vanillic Acid	Acetyl Syringaldehyde	Acetyl Syringic Acid	Acetyl Carboxyvanillic Acid	Acetyl Acetovanillone	Acetyl Acetosyringone
1	0.0677	0.1026	0.0908	0.1891	0	0.0080	0.0111
2	0.0713	0.0907	0.0918	0.1751	0	0.0042	0.0050
3	0.0727	0.1233	0.0909	0.2208	0	0.0092	0.0139
4	0.0577	0.0630	0.0867	0.1563	0	0.0041	0.0049
Average	0.0545	0.0984	0.0772	0.1900	0	0.0064	0.0087

Acetyl Propiovanillone	Acetyl Propiosyringone	Diacetyl Dimethoxyhydroquinone	Vanillin	Vanillic Acid	Syringaldehyde	Syringic Acid	Carboxyvanillic Acid
0.0037	0.0065	0.0000	0	0	0.0045	0.0033	0
0.0030	0.0050	0.0000	0.0033	0	0.0200	0.0125	0
0.0036	0.0063	0.0035	0.0029	0	0.0128	0.0095	0.0025
0.0032	0.0055	0.0000	0.0031	0	0.0147	0.0087	0
0.0034	0.0058	0.0009	0.0031	0	0.0130	0.0085	0.0025

Dimethoxybenzoquinone	Methoxymaleic acid	Vanillic Acid Dimer	Diacetyl Propanolguaiacol	Diacetyl Propanolsyringol	Propylsyringol	Acetyl Vanillic Acid Dimer
0.0785	0.0188	0	0	0.0036	0	0.0014
0.0746	0.0219	0	0	0.0043	0	0.0010
0.0943	0.0267	0	0	0.0023	0	0.0014
0.0727	0.0215	0	0	0.0037	0	0.0012
0.0800	0.0222	0	0	0.0035	0	0.0013

Biological Conversion

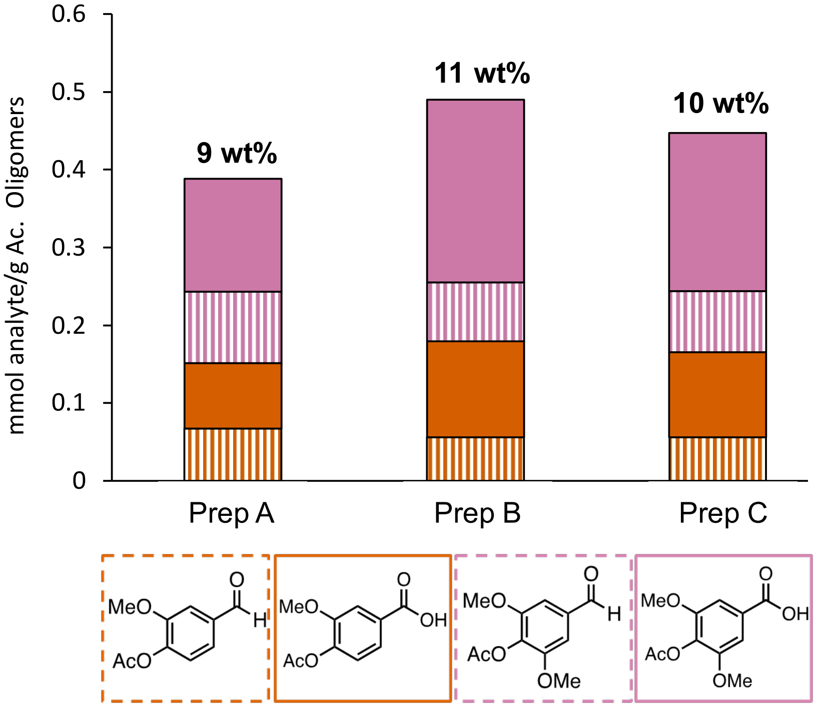

Figure S7. Composition of oxidation products from the oxidation of acetylated poplar RCF oil, prior to base treatment and use in biological conversion experiments. Three replicates (**A-C**) of the autooxidation reactions were performed, each using ~40 mg of starting material. A portion of each reaction was removed for analysis. The final masses used for bioconversion experiments are listed in the table below.

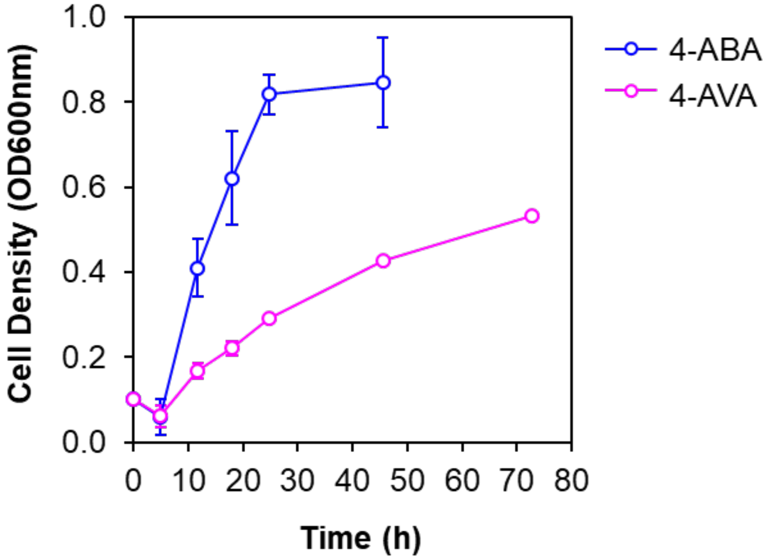

Figure S8. *P. putida* KT2440 wild-type utilizes acetyl benzoate (4-ABA) or acetyl vanillate (4-AVA) as the sole source of carbon and energy. Cultures were grown in shaken flasks with M9 minimal medium + 5 mM of model aromatic compound, and optical density at 600 nm was used as a proxy for bacterial growth. Error bars represent the standard deviation from the mean of three biological replicates.

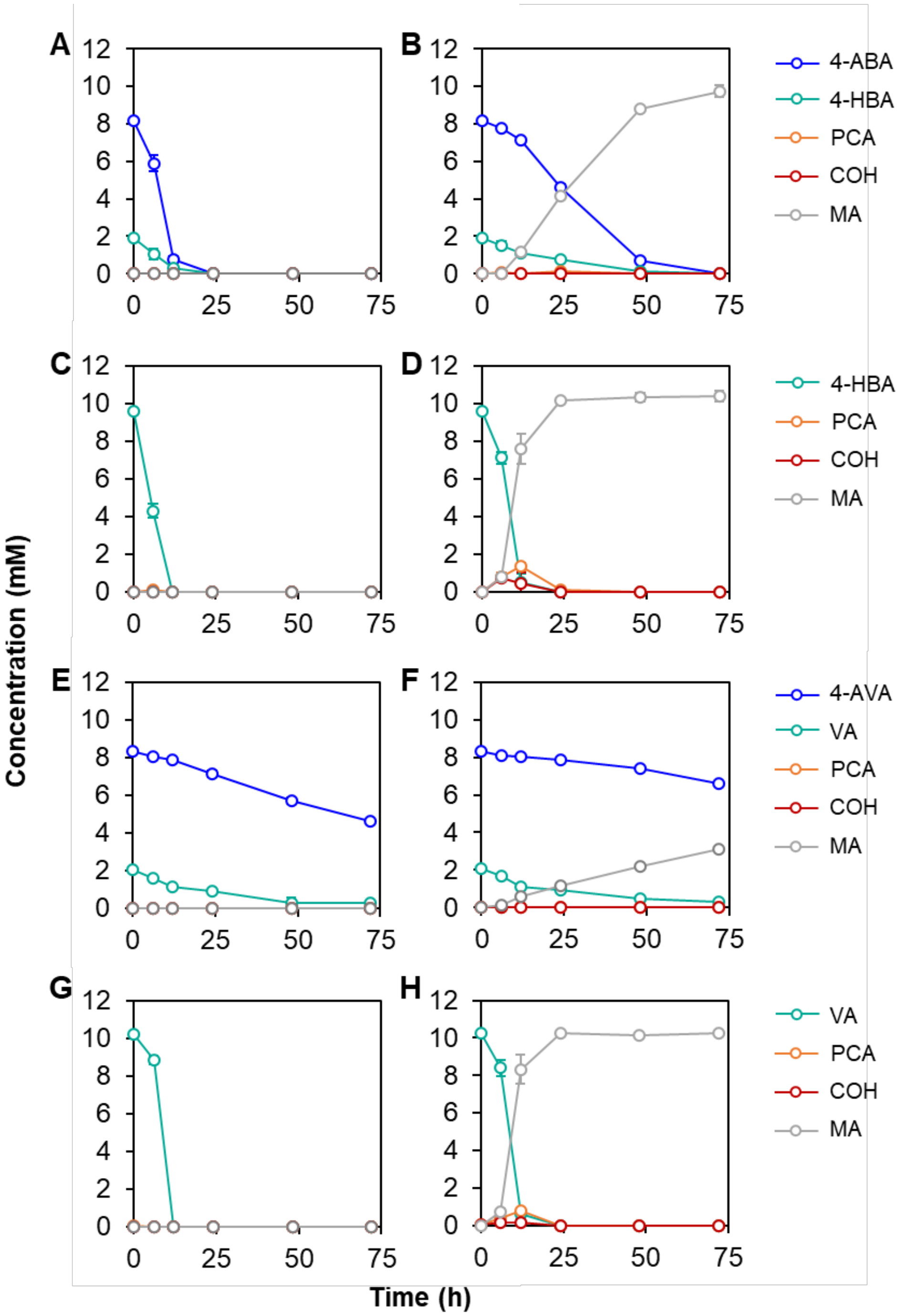

Figure S9. *P. putida* KT2440 wild-type was grown in M9 minimal medium with 5 mM glucose and 10 mM (A) acetyl benzoate (4-ABA), (C) 4-hydroxybenzoate (4-HBA), (E) acetyl vanillate (4-AVA), or (G) vanillate (VA), and it consumed the aromatic compounds in all cases. The engineered muconate-producing strain, CJ781, was grown in M9 minimal medium with 5 mM glucose and 10 mM (B) 4-ABA, (D) 4-HBA, (F) 4-AVA, or (H) VA, and it converted the aromatic compounds to a molar equivalent of muconate (MA) in all cases, with minimal accumulation of protocatechuate (PCA) or catechol (COH) intermediates. The presence of both acetylated and deacetylated compounds at time zero in (A, B, E, F) indicates some abiotic hydrolysis of 4-ABA and 4-AVA. Experiments with deacetylated compounds (4-HBA and VA) contained a molar equivalent (10 mM) of acetate to maintain carbon availability across samples. Error bars represent the standard deviation from the mean of three biological replicates.

Table S12. Mass of oxidation substrate used for biological conversion experiments below.

Autoxidation Replicate	Mass of Substrate for Biological Conversion (g)
Prep A	0.0328
Prep B	0.0322
Prep C	0.0321

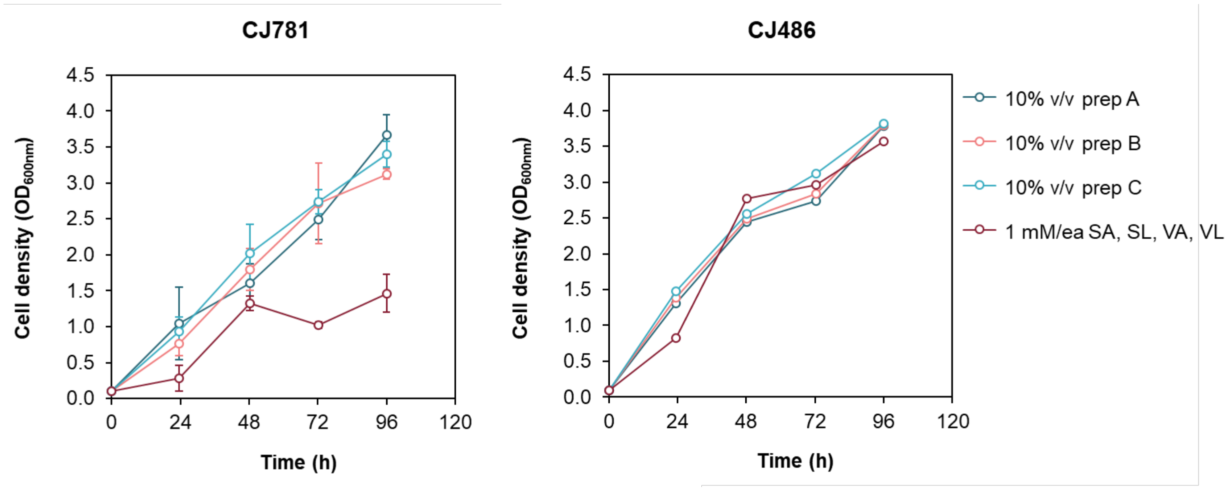

Figure S10. Cell density measurements (taken as the optical density at 600 nm, OD_{600nm}) for biological conversion experiments with *P. putida* strain CJ781(left) and CJ486 (right). Preps A-C refer to replicate autooxidation reactions with acetyl RCF oligomers. Culture media contained the aromatic sources as shown in the legend at right as well as 5 mM glucose fed every 24 h to support growth. Abbreviations: SA = syringate, SL = syringaldehyde, VA = vanillate, VL = vanillin.

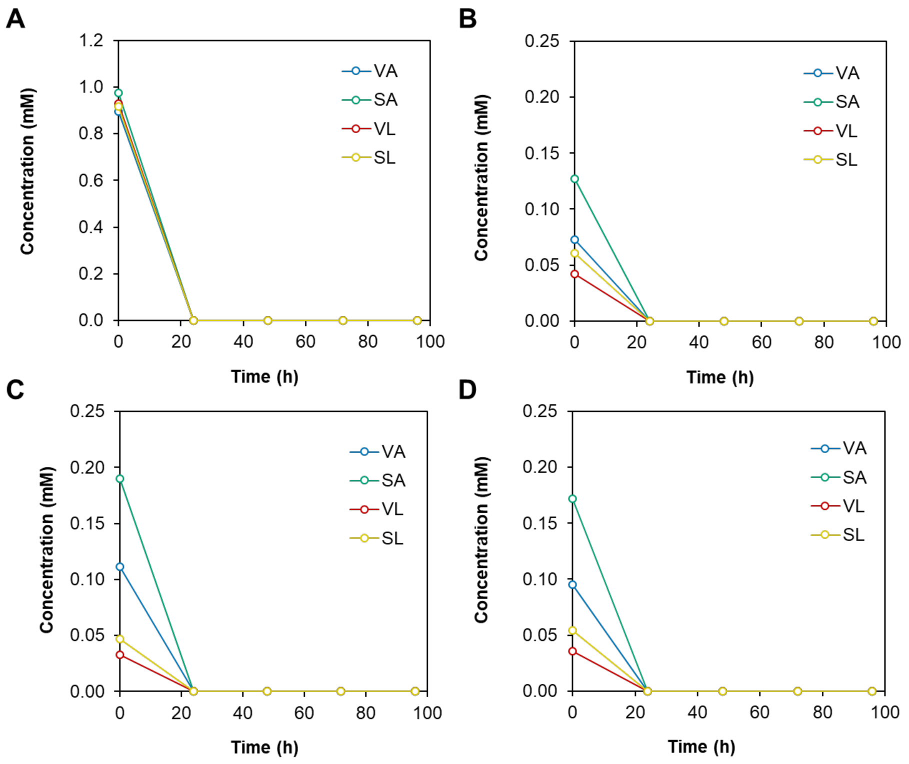

Figure S11. (A) Consumption of model compounds (1 mM/each syringate, syringaldehyde, vanillate, and vanillin) by *P. putida* CJ486. **(B-D)** Consumption of aromatic monomers from oxidized acetyl RCF oligomers by *P. putida* CJ486, where each graph represents a cultivation with a different autooxidation reaction replicate. Abbreviations: SA = syringate, SL = syringaldehyde, VA = vanillate, VL = vanillin.

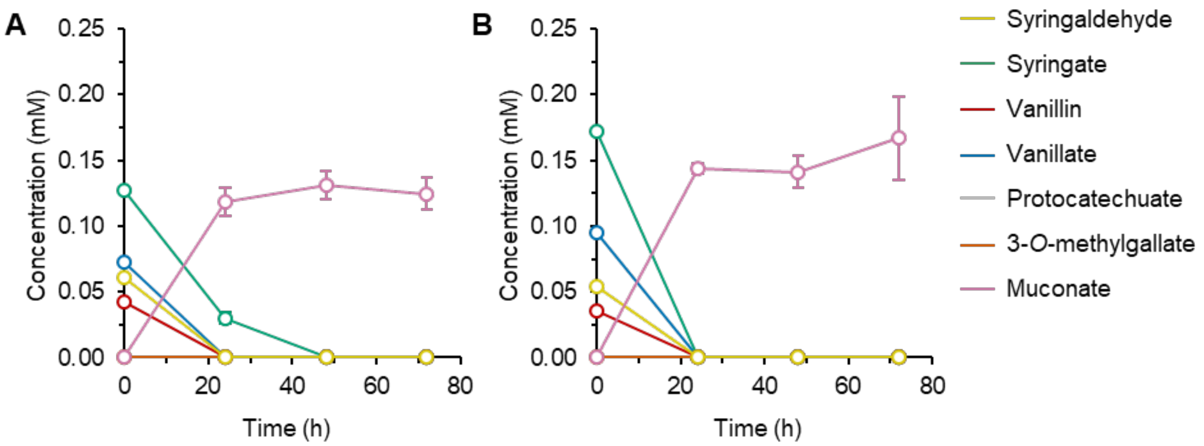

Figure S12. Strain CJ781 produces muconate from vanillate and vanillin, and produces biomass from syringate and syringaldehyde, when grown in M9 + 10% v/v oxidation products from acetylated poplar RCF. Each figure **(A)** and **(B)** represents one poplar oligomers oxidation reaction under the standard conditions. Results from a third reaction are depicted in Figure 5C. Each data point represents an average value and standard deviation from three biological replicates.

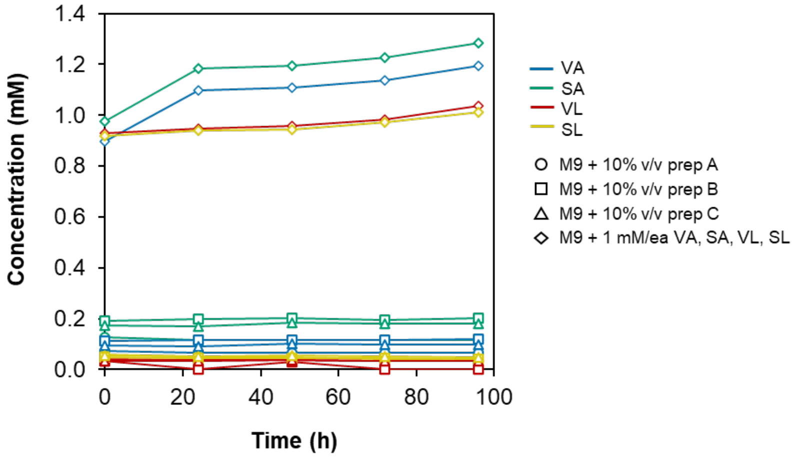

Figure S13. Cell-free media were incubated under the same conditions as the *P. putida* cultures, and they exhibited minimal changes in the concentration of vanillate (VA), syringate (SA), vanillin (VL), and syringaldehyde (SL).

Table S13. Composition of base-treated, oxidized acetyl RCF oil preparations that were used for bioconversion experiments in *P. putida.*

Compound	Concentration in base-treated, oxidized acetyl RCF oil (mM)		
	Prep A	Prep B	Prep C
Acetyl vanillic acid	0.00	0.00	0.00
Acetyl vanillin	0.00	0.00	0.00
Acetyl syringic acid	0.00	0.00	0.00
Acetyl syringic acid	0.00	0.00	0.00
Vanillic acid	0.67	1.15	1.07
Vanillin	0.39	0.32	0.35
Syringic acid	1.00	1.90	1.86
Syringaldehyde	0.52	0.44	0.48
Acetic acid	92.8	52.0	56.2

Table S14. Bacterial strains used in this study.

Strain	Genotype	Construction details
**KT2440**	*P. putida* KT2440	Wild-type strain
CJ486	*P. putida* KT2440 *fpvA*:P_{tac}:*vanAB*	Previously described (Notonier *et al.*, 2021).
CJ781	*P. putida* KT2440 Δ*catRBCA*::P_{tac}:*catA* Δ*pcaHG*::P_{tac}:*aroY*:*ecdBD* Δ*crc* Δ*pobAR* Δ*fpvA*::P_{tac}:*praI*:*vanAB*	Previously described (Kuatsjah *et al.*, 2022).

Table S15. Numerical data used to generate Figure 5 and Figure S9. "av" indicates the mean of three replicates; "sd" indicates the standard deviation from the mean. Abbreviations: GA = gallate, PCA = protocatechuate, COH = catechol, 3MGA = 3-*O*-methylgallate, VA = vanillate, SA = syringate, VL = vanillin, SL = syringaldehyde, MA = total muconate (*cis,cis* plus *cis,trans*).

CJ781 - 10% v/v prep A																		
Time (h)	GA_av	GA_sd	PCA_av	PCA_sd	COH_av	COH_sd	3MGA_av	3MGA_sd	VA_av	VA_sd	SA_av	SA_sd	VL_av	VL_sd	SL_av	SL_sd	MA_av	MA_sd
0	0		0		0		0		0.07278787		0.12702022		0.04236807		0.06062897		0	
24	0	0	0	0	0	0	0	0	0	0	0.03011656	0.0040546	0	0	0	0	0.1184337	0.01106493
48	0	0	0	0	0	0	0	0	0	0	0	0	0	0	0	0	0.13122099	0.01063519
72	0	0	0	0	0	0	0	0	0	0	0	0	0	0	0	0	0.12460048	0.01194857
**CJ781 - 10 % v/v prep B**																		
Time (h)	GA_av	GA_sd	PCA_av	PCA_sd	COH_av	COH_sd	3MGA_av	3MGA_sd	VA_av	VA_sd	SA_av	SA_sd	VL_av	VL_sd	SL_av	SL_sd	MA_av	MA_sd
0	0		0		0		0		0.11168491		0.19031551		0.03284013		0.04682801		0	
24	0	0	0	0	0	0	0.01078863	0.01868646	0	0	0.0115082	0.01993279	0	0	0	0	0.15201704	0.02678557
48	0	0	0	0	0	0	0	0	0	0	0	0	0	0	0	0	0.16393824	0.02516997
72	0	0	0	0	0	0	0	0	0	0	0	0	0	0	0	0	0.1838229	0.03644089
**CJ781 - 10 % v/v prep C**																		
Time (h)	GA_av	GA_sd	PCA_av	PCA_sd	COH_av	COH_sd	3MGA_av	3MGA_sd	VA_av	VA_sd	SA_av	SA_sd	VL_av	VL_sd	SL_av	SL_sd	MA_av	MA_sd
0	0		0		0		0		0.09516636		0.17188646		0.0355423		0.05388121		0	
24	0	0	0	0	0	0	0	0	0	0	0	0	0	0	0	0	0.14381966	0.00422222
48	0	0	0	0	0	0	0	0	0	0	0	0	0	0	0	0	0.1412366	0.01249765
72	0	0	0	0	0	0	0	0	0	0	0	0	0	0	0	0	0.16682915	0.03152962
**CJ781 - 1 mM/ea SA, SL, VA, VL**																		
Time (h)	GA_av	GA_sd	PCA_av	PCA_sd	COH_av	COH_sd	3MGA_av	3MGA_sd	VA_av	VA_sd	SA_av	SA_sd	VL_av	VL_sd	SL_av	SL_sd	MA_av	MA_sd
0	0		0		0		0		0.89619034		0.97570796		0.92787179		0.91737327		0	
24	0	0	0.01048581	0.01816196	0	0	0.3831799	0.04988923	0	0	0.78826061	0.15228459	0	0	0	0	1.91852588	0.07287434
48	0	0	0	0	0	0	0.09484162	0.03230703	0	0	0.25130096	0.09545022	0	0	0	0	2.00944733	0.15692677
72	0	0	0	0	0	0	0	0	0	0	0.02730438	0.02559705	0	0	0	0	2.0684348	0.16377386
96	0	0	0	0	0	0	0	0	0	0	0	0	0	0	0	0	2.05160417	0.09603326

CJ486 - 10% v/v prep A								
Time (h)	GA	PCA	COH	3MGA	VA	SA	VL	SL
0	0	0	0	0	0.07278787	0.12702022	0.04236807	0.06062897
24	0	0	0	0	0	0	0	0
48	0	0	0	0	0	0	0	0
72	0	0	0	0	0	0	0	0
96	0	0	0	0	0	0	0	0
**CJ486 - 10% v/v prep B**								
Time (h)	GA	PCA	COH	3MGA	VA	SA	VL	SL
0	0	0	0	0	0.11168491	0.19031551	0.03284013	0.04682801
24	0	0	0	0	0	0	0	0
48	0	0	0	0	0	0	0	0
72	0	0	0	0	0	0	0	0
96	0	0	0	0	0	0	0	0
**CJ486 - 10% v/v prep C**								
Time (h)	GA	PCA	COH	3MGA	VA	SA	VL	SL
0	0	0	0	0	0.09516636	0.17188646	0.0355423	0.05388121
24	0	0	0	0	0	0	0	0
48	0	0	0	0	0	0	0	0
72	0	0	0	0	0	0	0	0
96	0	0	0	0	0	0	0	0
**CJ486 - 1 mM/ea SA, SL, VA, VL**								
Time (h)	GA	PCA	COH	3MGA	VA	SA	VL	SL
0	0	0	0	0	0.89619034	0.97570796	0.92787179	0.91737327
24	0	0	0	0	0	0	0	0
48	0	0	0	0	0	0	0	0
72	0	0	0	0	0	0	0	0
96	0	0	0	0	0	0	0	0

Table S16. Autoxidation product multiple reaction monitoring (MRM) transitions. Optimized MRM transitions (quantifying and qualifying) and respective fragmentor voltages and collision energies for quantitation of products formed from autoxidation.

**Analyte**	**Ionization Mode**	**Precursor Ion (*m/z*)**	**Fragmentor (V)**	**MRM quantifying transition**	**CE (V)**	**MRM qualifying transition**	**CE (V)**
methoxy maleic acid	negative	145	45	145 → 101	4	145 → 57.1	8
vanillin	negative	151	65	151 → 136	12	151 → 92	20
vanillic acid	negative	167	80	167 → 151.9	12	167 → 123	12
carboxy vanillic acid	negative	211	80	211 → 152	16	211 → 108	24
acetyl carboxyvanillic acid	negative	253	50	253 → 193	8	253 → 211	0
acetyl vanillic acid dimer	negative	417	170	417 → 274	36	417 → 333	40
dimethoxy benzoquinone	positive	169	115	169 → 141	12	169 → 69	50
syringaldehyde	positive	183	55	183.1 → 123	12	183.1 → 77.1	28
4-propyl syringol	positive	197	85	197.1 → 165	8	197.1 → 43.2	16
syringic acid	positive	199	60	199.1 → 140	16	199.1 → 155	8
acetyl acetovanillone	positive	209	65	209.1 → 167	4	209.1 → 43.1	20
acetyl propyl guaiacol	positive	209	85	209.1 → 43.1	24	209.1 → 167	4
acetyl propiovanillone	positive	223	50	223.1 → 181	8	223.1 → 57.1	20
acetyl acetosyringone	positive	239	50	239.1 → 197	4	239.1 → 43.1	24
acetyl propylsyringol	positive	239	85	239.1 → 197.1	8	239.1 → 43.1	28
acetyl propiosyringone	positive	253	50	253.1 → 211	8	253.1 → 57.1	20
diacetyl dimethoxy hydroquinone	positive	255	95	255.1 → 213	8	255.1 → 171	16
diacetyl propanol guaiacol	positive	267	60	267.1 → 165	12	267.1 → 207	8
diacetyl α-hydroxy propiovanillone	positive	281	92	281.1 → 147	28	281.1 → 151	28
diacetyl propanolsyringol	positive	297	75	297.1 → 237.1	8	297.1 → 167	28
vanillic acid dimer	positive	335	150	335.1 → 273	12	335.1 → 215	20
diacetyl bivanillyl	positive	359	114	359.2 → 43.1	44	359.2 → 151	24
acetyl vanillin dimer	positive	387	65	387.1 → 303	12	387.1 → 215	32
acetyl beta 5 model	positive	415	87	415.2 → 165	24	415.2 → 207	20
diacetyl propyl guaiacol dimer	positive	415	70	415.2 → 331.2	16	415.2 → 267.1	28
diacetyl pinoresinol	positive	443	129	443.2 → 137.1	40	443.2 → 323.1	12

Table S17. Acetyl Propylguaiacol (**2**) Oxidation

Run	% yield						
	Acetyl Propylguaiacol[b]	Acetyl Vanillic Acid[a]	Acetyl Vanillin[a]	Acetyl Propiovanillone[b]	Methoxymaleic Acid[b]	Vanillic Acid[b]	Vanillin[b]
a	31.0	14.1	25.1	24.8	0.3	0	0.1
b	12.1	33.6	9.5	9.5	0.7	9	1.4
c	22.2	18.0	26.2	25.1	0.3	0	0.1
d	3.5	49	10.3	18.9	0.8	0	0
average	**17**	**29**	**18**	**20**	**0.5**	**2**	**0.4**

[a]quantified by LC-MS [b]quantified by UHPLC

Table S18. Acetyl Propylsyringol (**3**) Oxidation

Run	% yield						
	Acetyl Syringic Acid[a]	Acetyl Syring-aldehyde[a]	Acetyl Propio-syringone[b]	Methoxy-maleic Acid[b]	Dimethoxy benzoquinone[b]	Syringic Acid[b]	Syring-aldehyde[b]
a	8.4	22.5	24.5	0.2	2.6	0	0.2
b	58.7	1.2	7.6	1.4	11.1	0.2	0
c	21.6	21.4	24.5	0.5	3.3	0.1	0.2
d	10.1	19.6	25.2	0.3	2.6	0	0.2
average	**25**	**16**	**21**	**0.6**	**5**	**0.1**	**0.2**

[a]quantified by LC-MS [b]quantified by UHPLC

Table S19. Diacetyl Bivanillyl (**4**, β-1) Oxidation

Run	% yield				
	Acetyl Vanillic Acid[a]	Acetyl Vanillin[a]	Methoxymaleic Acid[b]	Vanillic Acid[b]	Vanillin[b]
a	46.2	18.1	1.2	0.8	0.3
b	58.3	2.8	1.3	3.5	0.1
c	49.4	15.9	1.1	0.9	0.2
d	61.4	1.7	1.4	4	0.1
average	**54**	**10**	**1**	**2**	**0.2**

[a]quantified by LC-MS [b]quantified by UHPLC

Table S20. Acetyl β-5 Model (**5**) Oxidation

Run	% yield					
	Acetyl Vanillic Acid[a]	Acetyl Vanillin[a]	Acetyl Carboxyvanillic Acid[b]	Methoxymaleic Acid[b]	Vanillic Acid[b]	Vanillin[b]
a	1.0	7.0	0	0.4	0	0.1
b	5.0	8.2	0	1.0	0	0.1
c	0.7	6.4	0.1	0	0	0
d	0.4	4.5	0.1	0	0	0
average	**2**	**7**	**0**	**0.4**	**0**	**0**

[a]quantified by LC-MS [b]quantified by UHPLC

Table S21. Diacetyl propylguaiacol dimer (**6**, 5-5) oxidation

Run	% yield				
	Acetyl Vanillic Acid[a]	Acetyl Vanillin[a]	Methoxymaleic Acid[b]	Acetyl Vanillic Acid Dimer[b]	Vanillic Acid Dimer[b]
a	0	0.1	0.6	0.6	0
b	0.1	0	0.5	2.7	0
c	0	0	0	0	0
d	0	0	0	0	0
average	**0**	**0**	**0.3**	**0.8**	**0**

[a]quantified by LC-MS [b]quantified by UHPLC

NMR Spectra.

^{1}H NMR quantification of EtOAc in RCF oils: A $CDCl_3$ solution of trimethyl 1,3,5-benzenetricarboxylate (0.2 mL of 10000 ppm stock) was added to the RCF oil sample (*ca*. 20 mg). An additional 0.3 mL of $CDCl_3$ was added to each sample, which was subsequently transferred into an NMR tube. ^{1}H NMR spectra were collected for each sample with 1 scan (Figs. S14-S15).

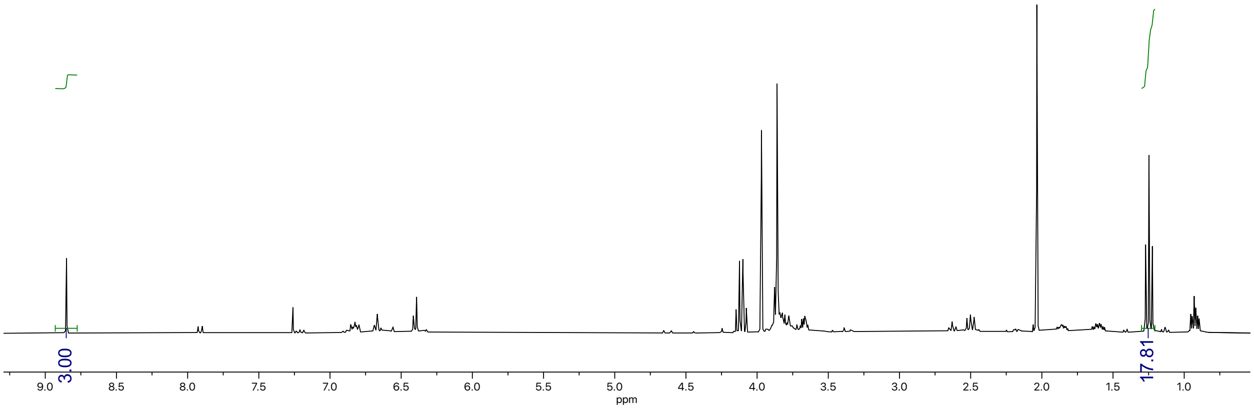

Figure S14. ^{1}H NMR spectrum of poplar RCF oil and trimethyl 1,3,5-benzenetricarboxylate (internal standard) in $CDCl_3$. Integrations of the Ar-*H* (internal standard) and CH_2CH_3 (EtOAc) protons are shown.

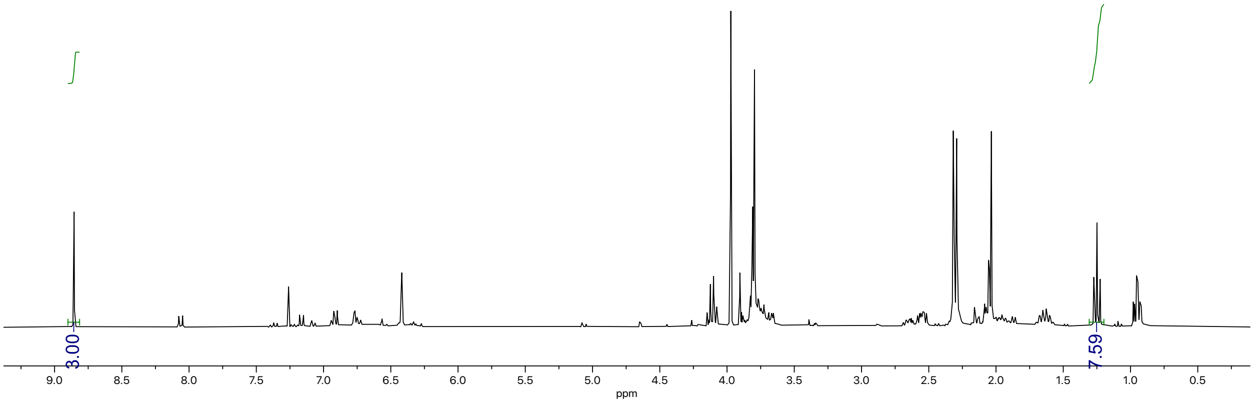

Figure S15. ^{1}H NMR spectrum of acetyl poplar RCF oil and trimethyl 1,3,5-benzenetricarboxylate (internal standard) in $CDCl_3$. Integrations of the Ar-*H* (internal standard) and CH_2CH_3 (EtOAc) protons are shown.

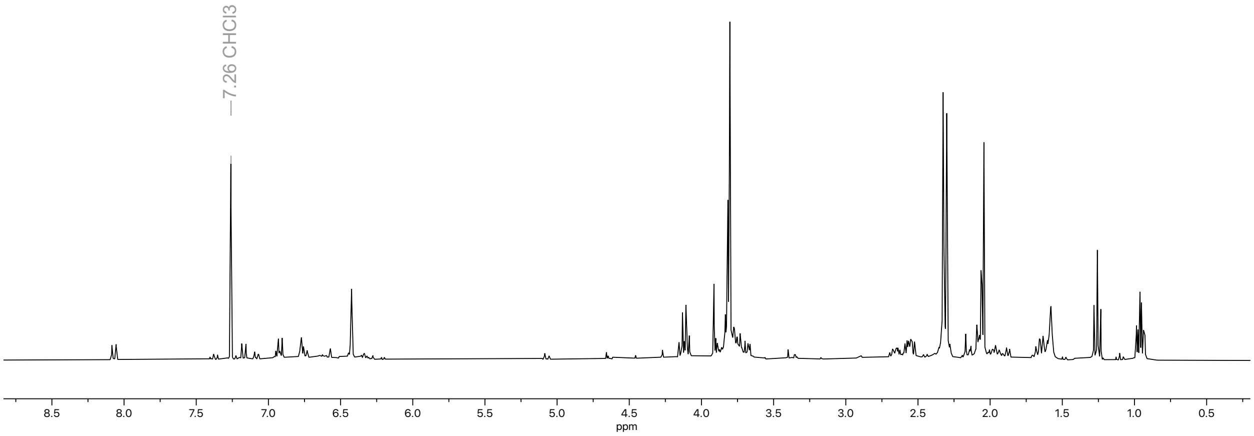

Figure S16. ^{1}H NMR of acetyl RCF oil in $CDCl_3$

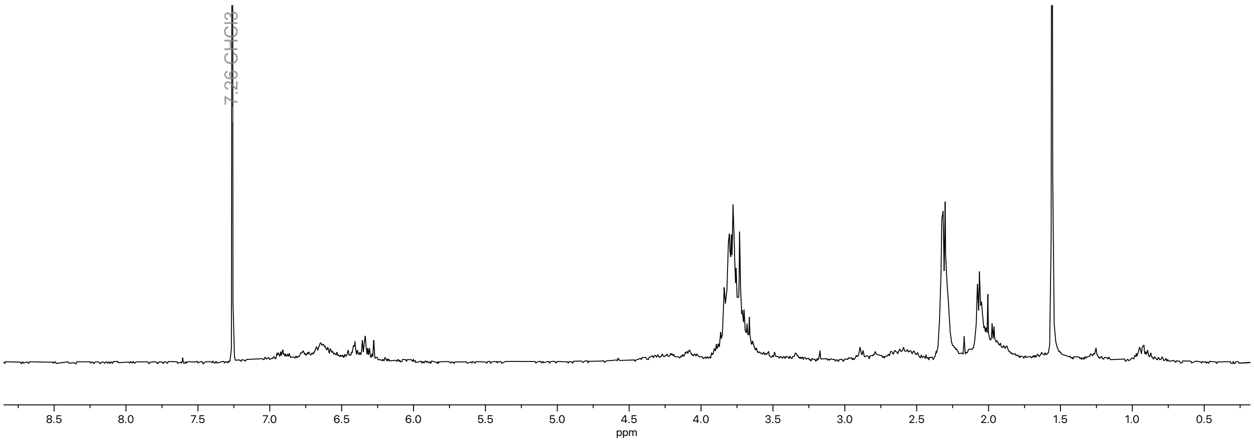

Figure S17. ^{1}H NMR of acetyl oligomer fraction in $CDCl_3$

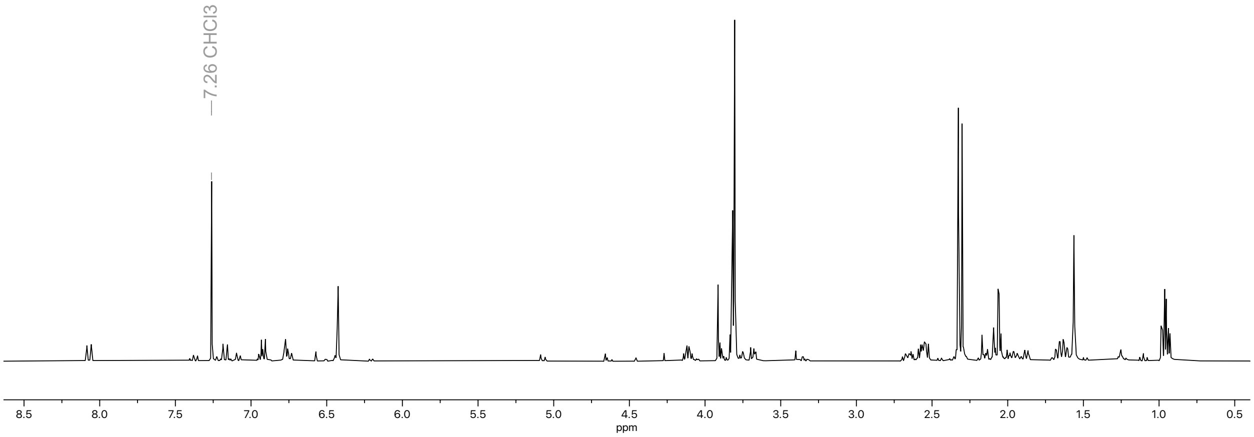

Figure S18. ^{1}H NMR of acetyl monomer fraction in $CDCl_3$

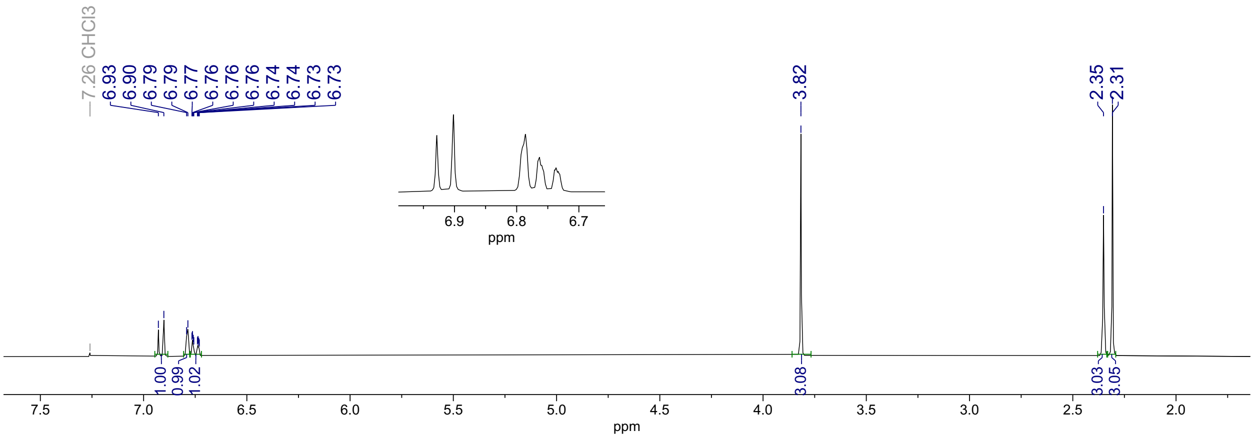

Figure S19. ^{1}H NMR spectrum of acetyl methylguaiacol in $CDCl_3$

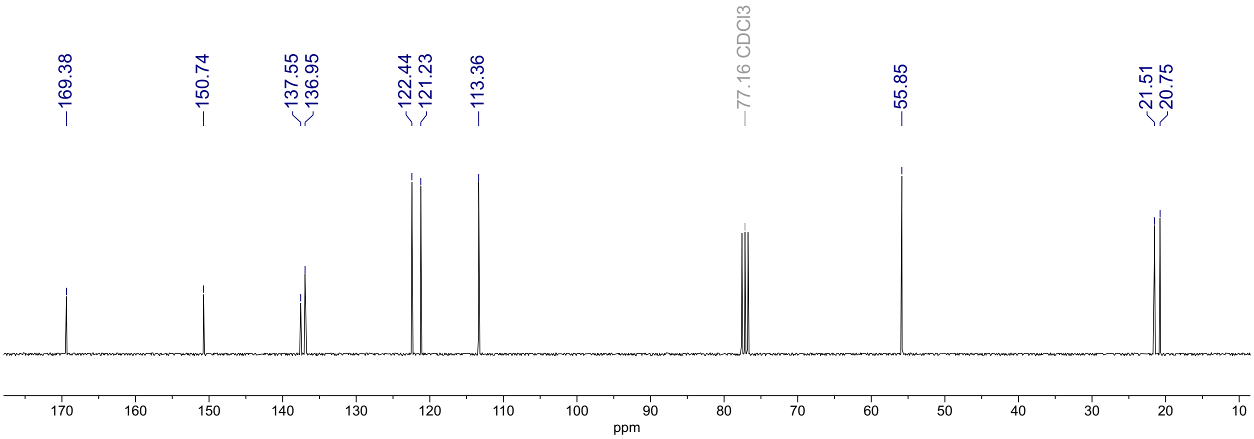

Figure S20. ^{13}C NMR spectrum of acetyl methylguaiacol in $CDCl_3$

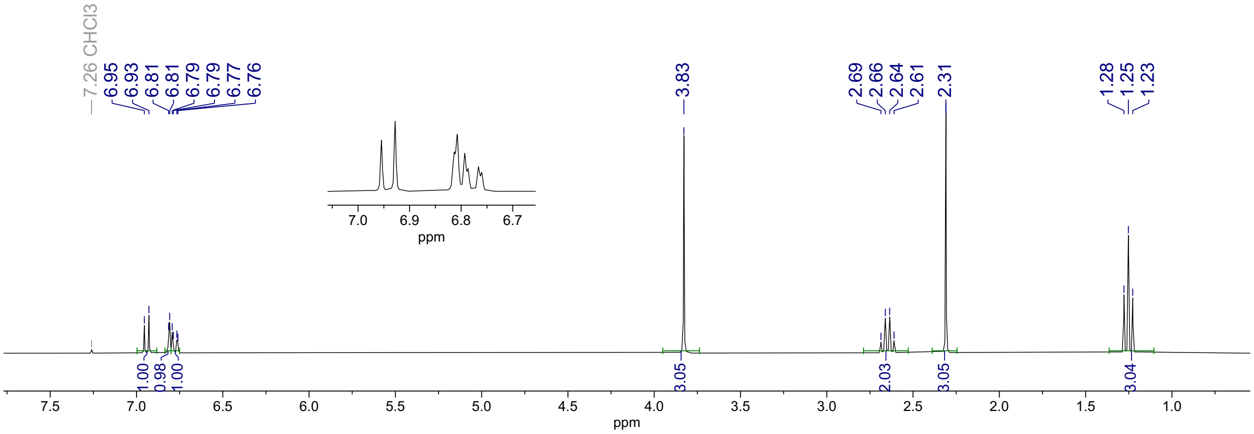

Figure S21. ^{1}H NMR spectrum of acetyl ethylguaiacol in $CDCl_3$

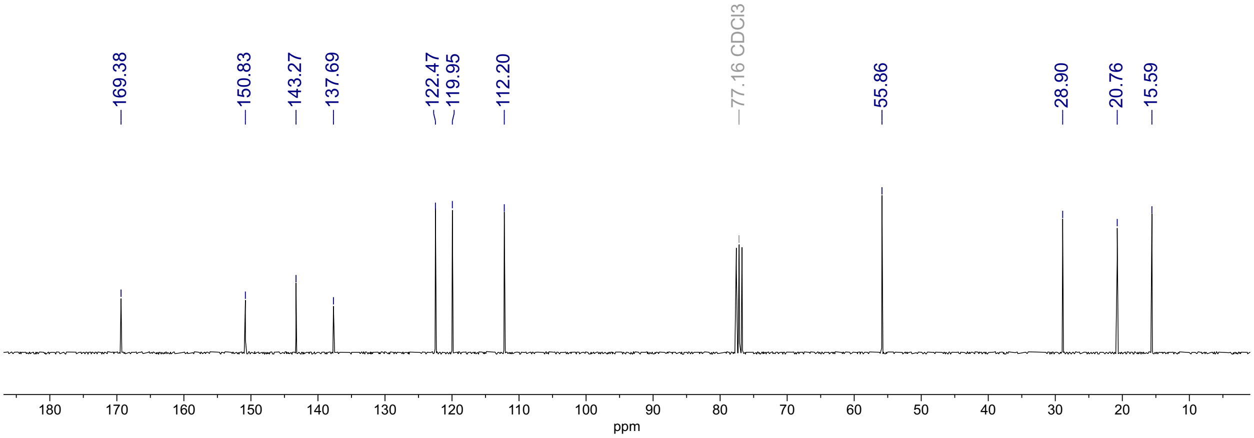

Figure S22. ^{13}C NMR spectrum of acetyl ethylguaiacol in $CDCl_3$

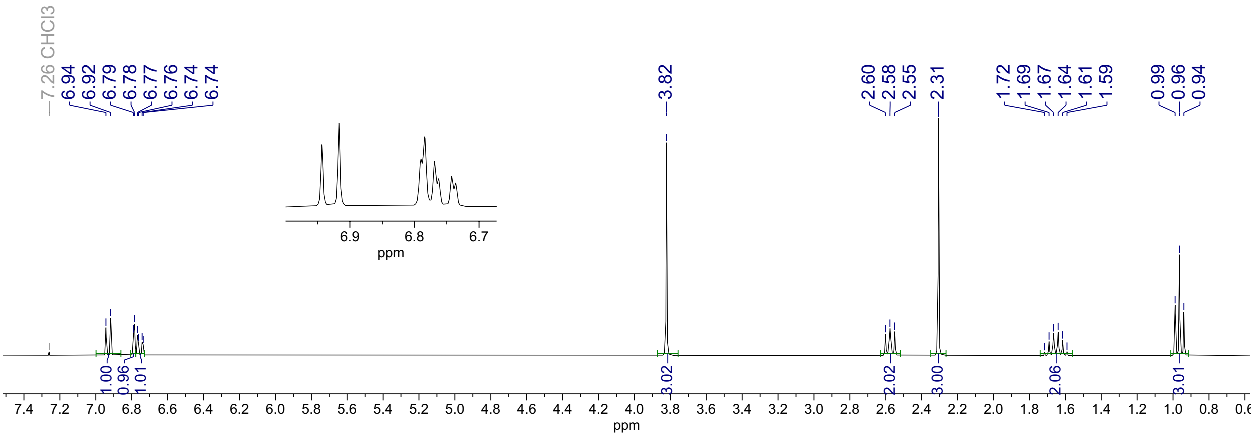

Figure S23. ^{1}H NMR spectrum of acetyl propylguaiacol in $CDCl_3$

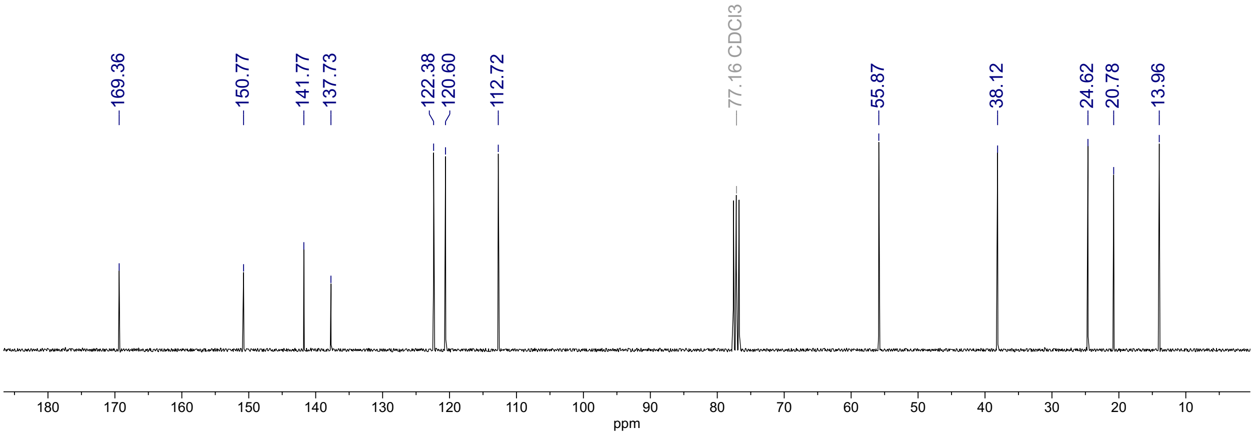

Figure S24. ^{13}C NMR spectrum of acetyl propylguaiacol in $CDCl_3$

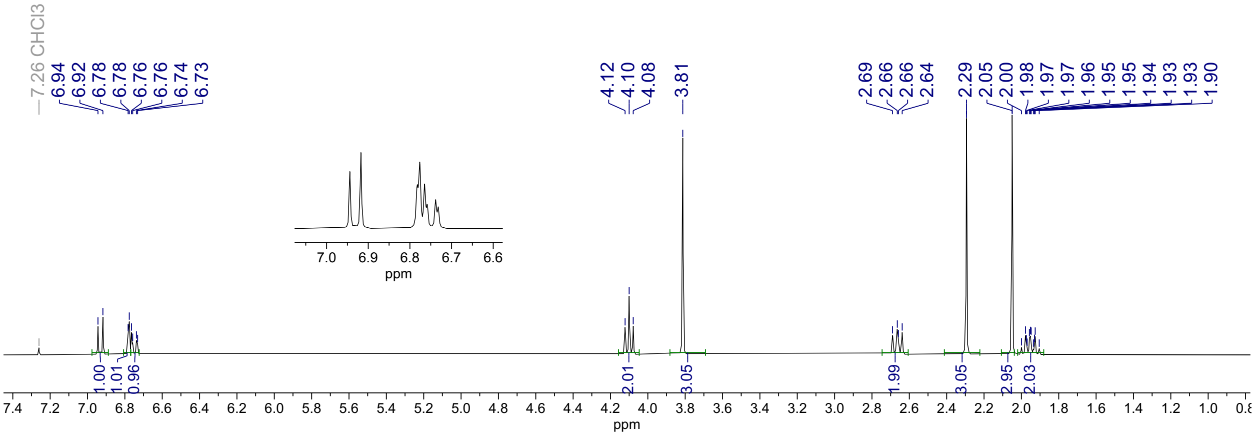

Figure S25. ^{1}H NMR spectrum of diacetyl propanolguaiacol in $CDCl_3$

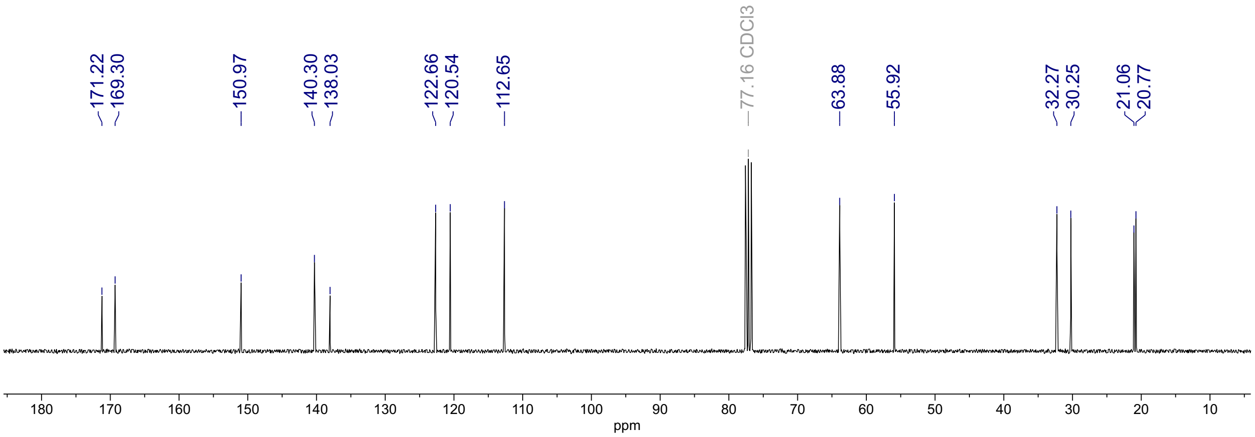

Figure S26. ^{13}C NMR spectrum of diacetyl propanolguaiacol in $CDCl_3$

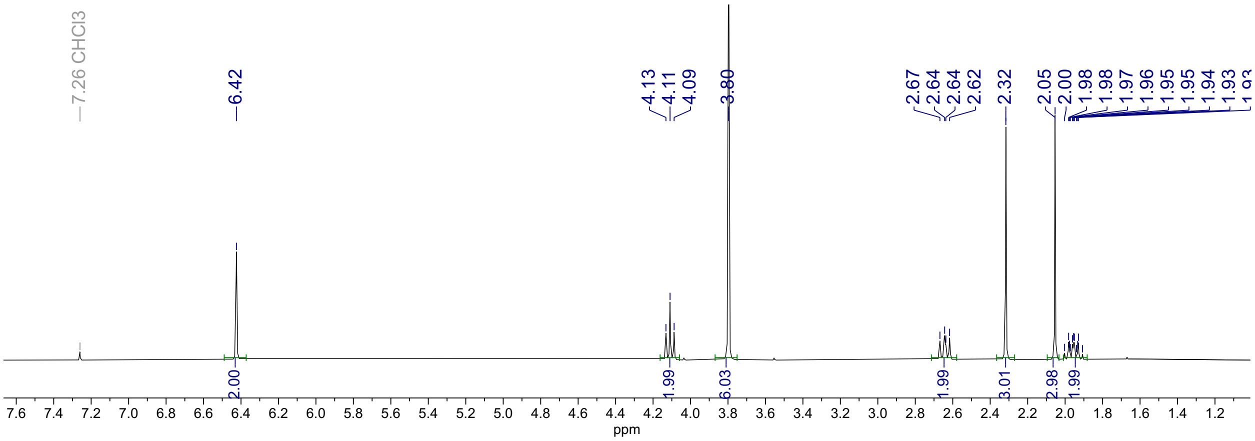

Figure S27. ^{1}H NMR spectrum of diacetyl propanolsyringol in $CDCl_3$

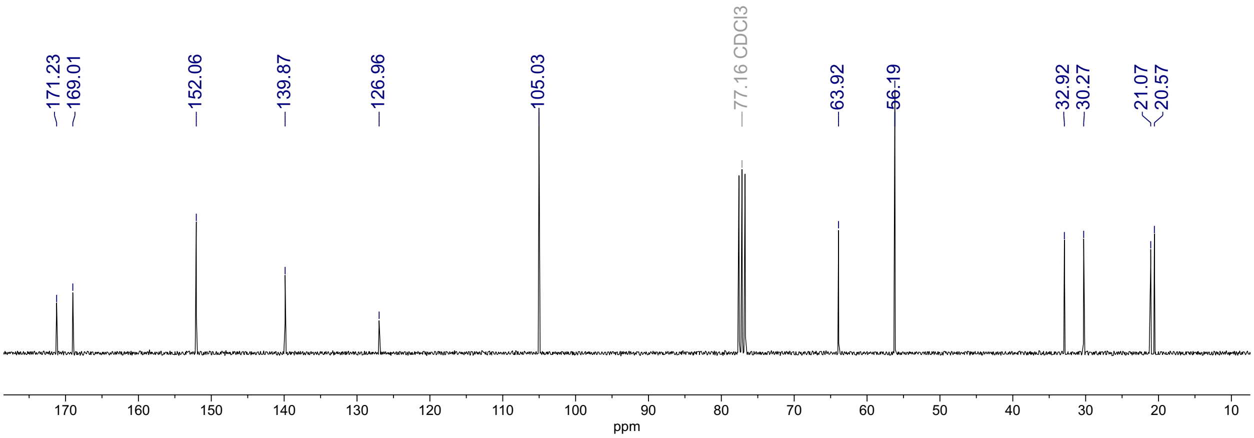

Figure S28. ^{13}C NMR spectrum of diacetyl propanolsyringol in $CDCl_3$

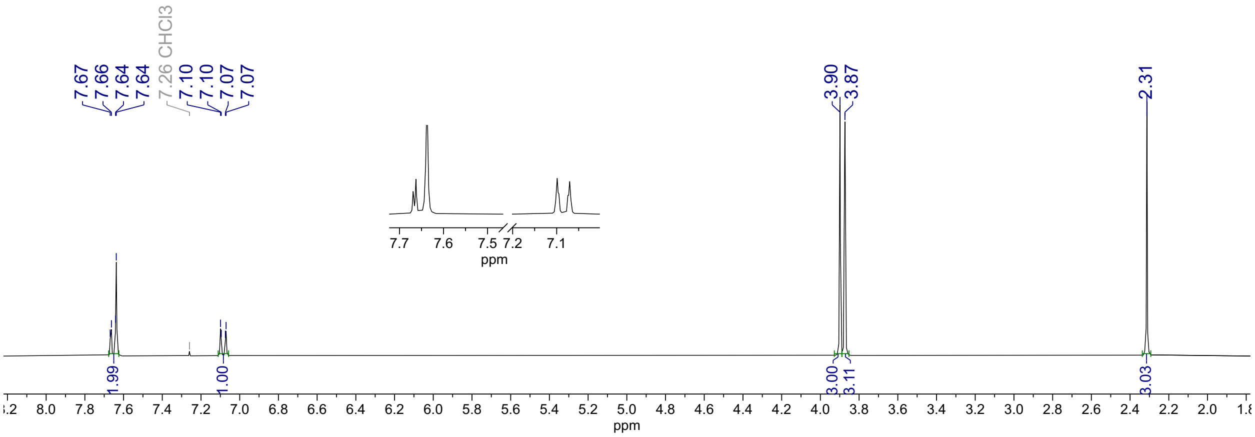

Figure S29. ^{1}H NMR spectrum of acetyl methylvanillate in $CDCl_3$

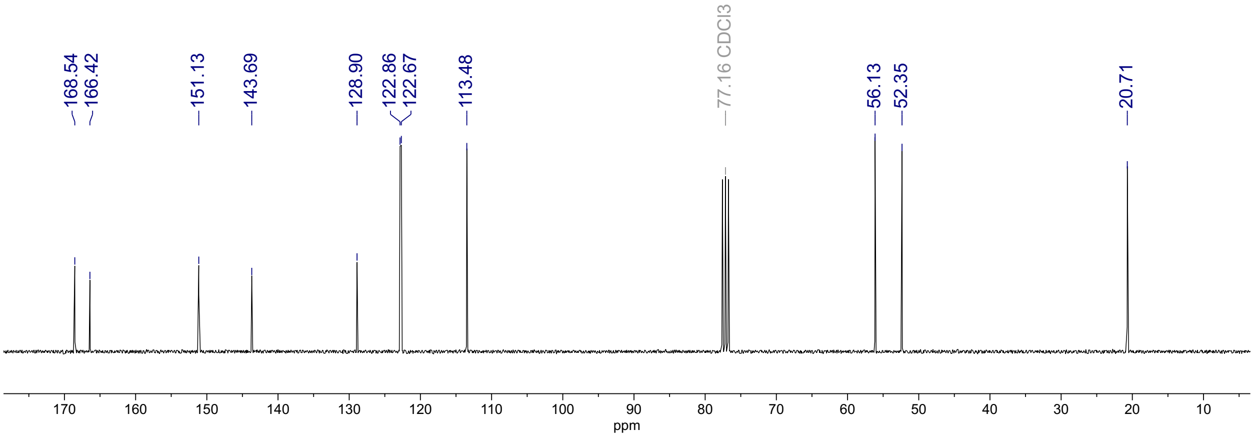

Figure S30. ^{13}C NMR spectrum of acetyl methylvanillate in $CDCl_3$

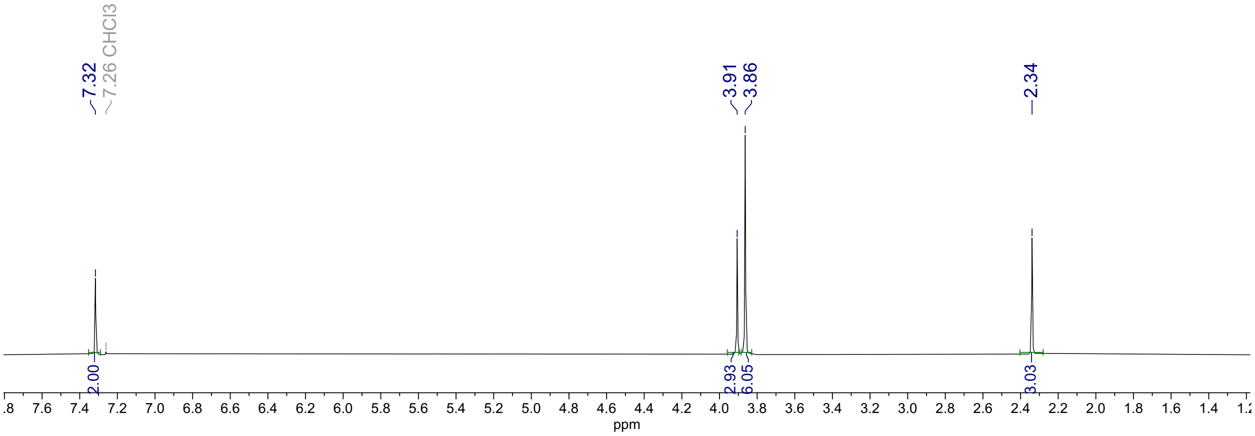

Figure S31. ^{1}H NMR spectrum of acetyl methylsyringate in $CDCl_3$

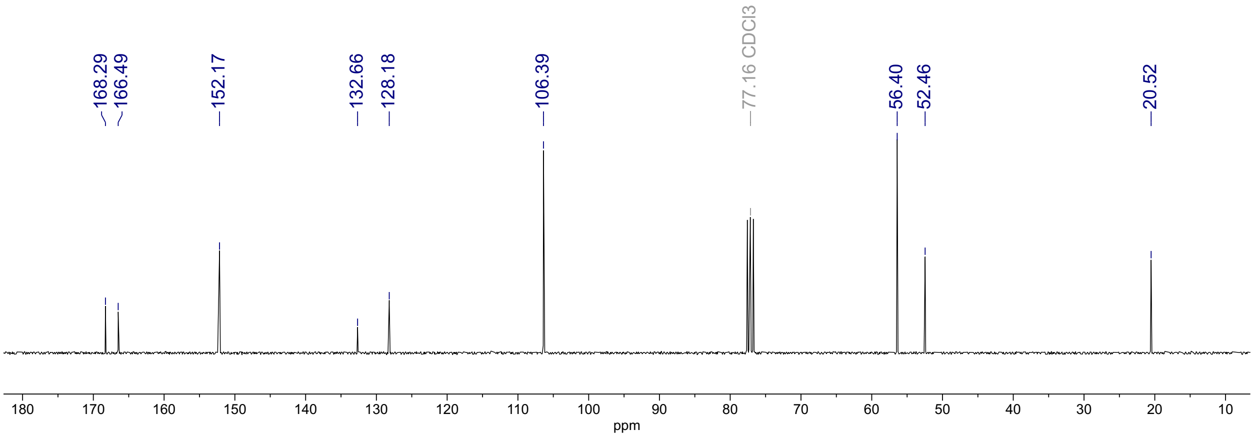

Figure S32. ^{13}C NMR spectrum of acetyl methylsyringate in $CDCl_3$

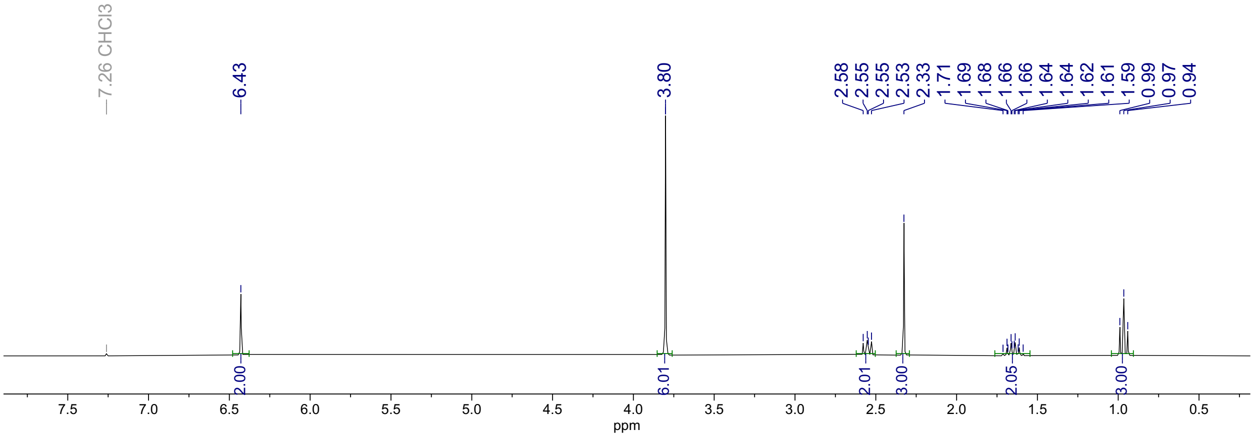

Figure S33. ^{1}H NMR spectrum of acetyl propylsyringol in $CDCl_3$

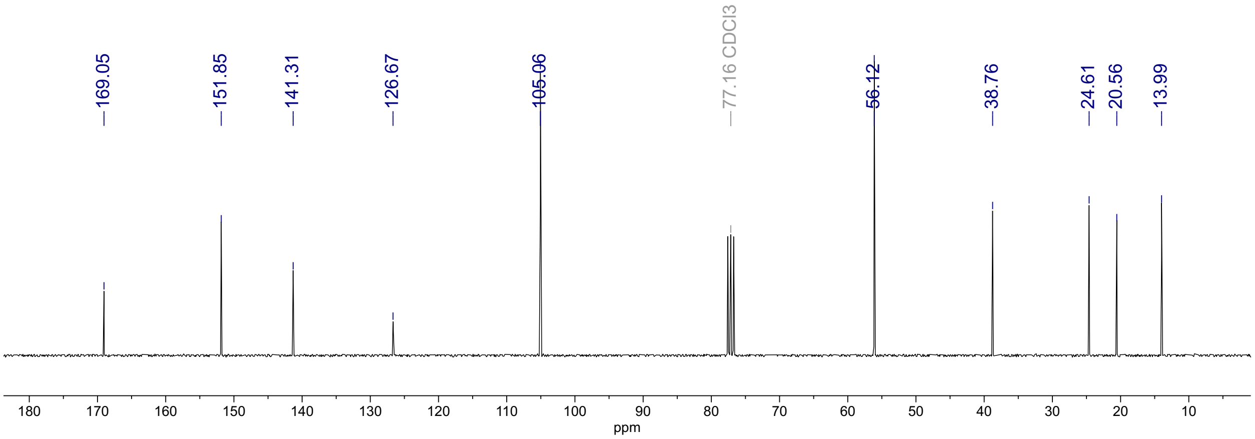

Figure S34. ^{13}C NMR spectrum of acetyl propylsyringol in $CDCl_3$

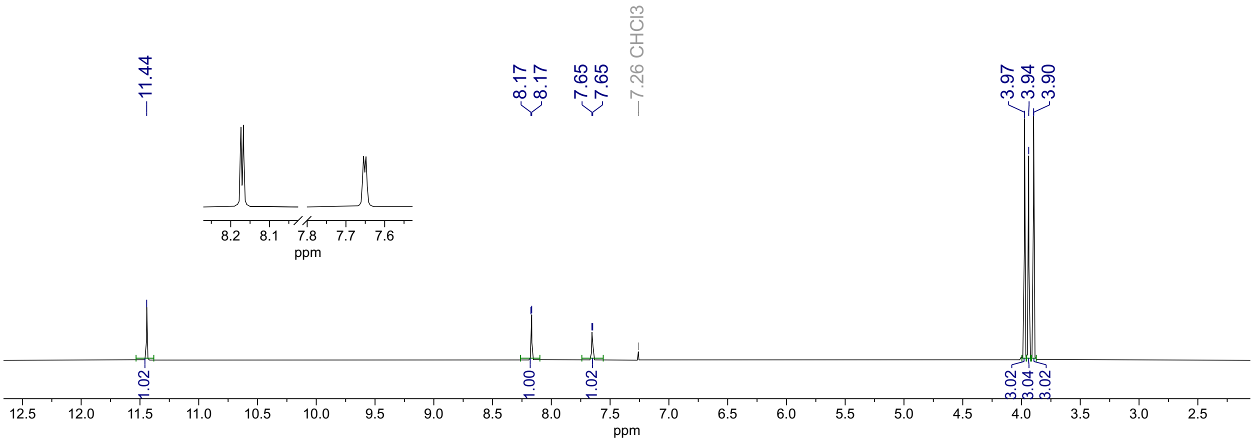

Figure S35. ^{1}H NMR spectrum of dimethyl carboxyvanillate in $CDCl_3$

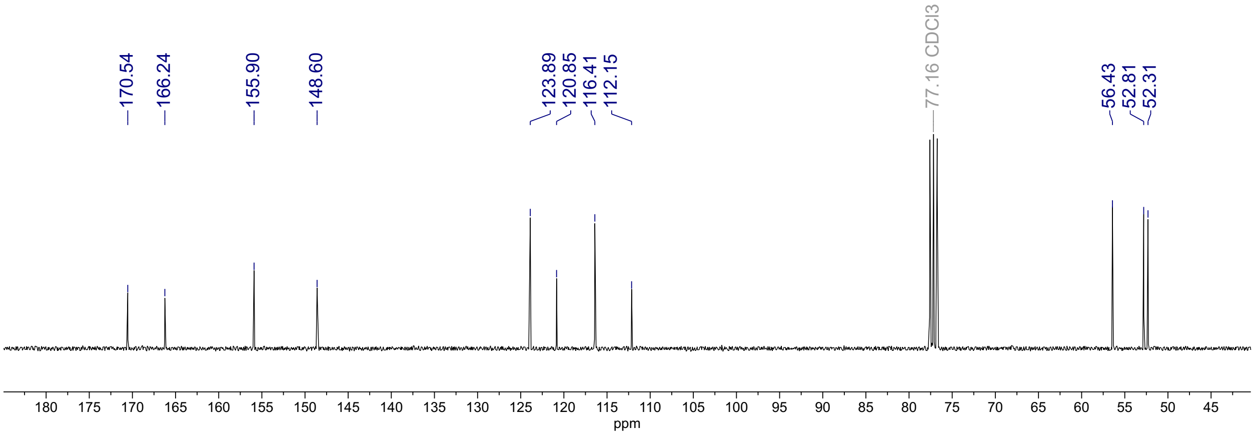

Figure S36. ^{13}C NMR spectrum of dimethyl carboxyvanillate in $CDCl_3$

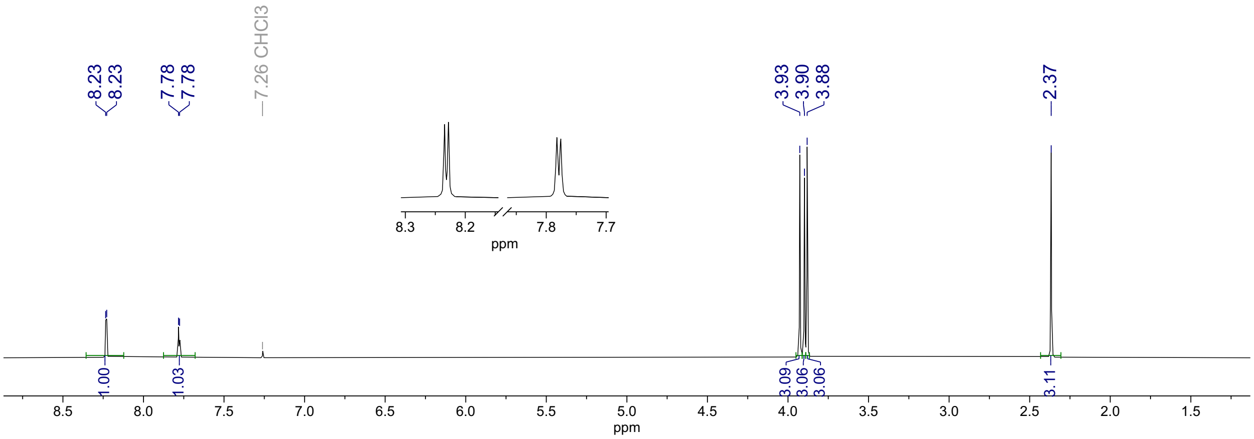

Figure S37. ^{1}H NMR spectrum of acetyl dimethyl carboxyvanillate in $CDCl_3$

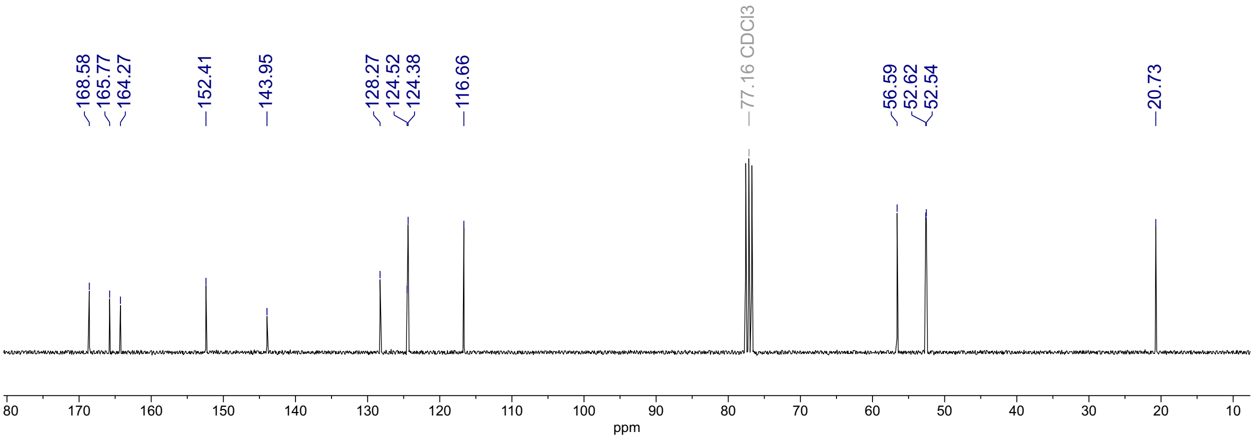

Figure S38. ^{13}C NMR spectrum of acetyl dimethyl carboxyvanillate in $CDCl_3$

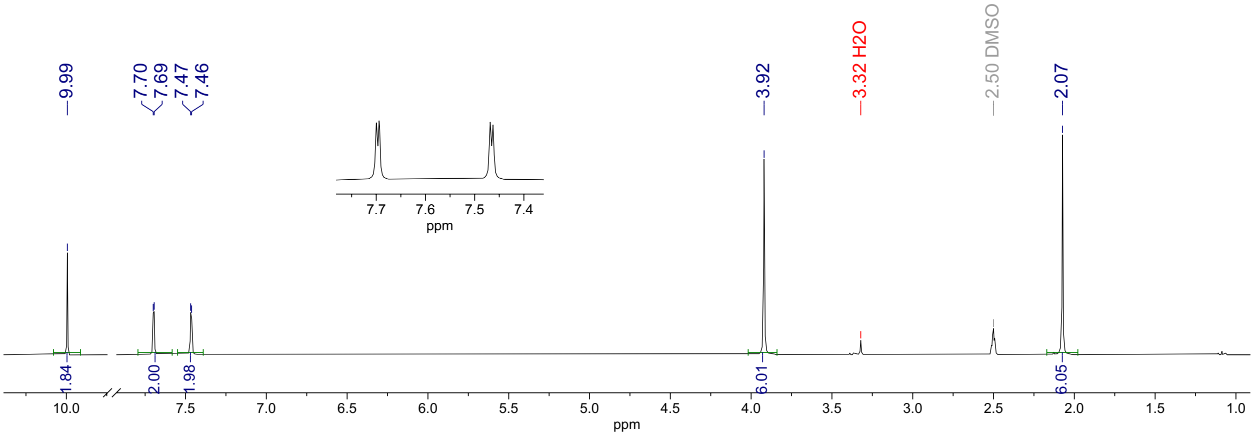

Figure S39. ^{1}H NMR spectrum of diacetyldehydrovanillin in DMSO-d_6

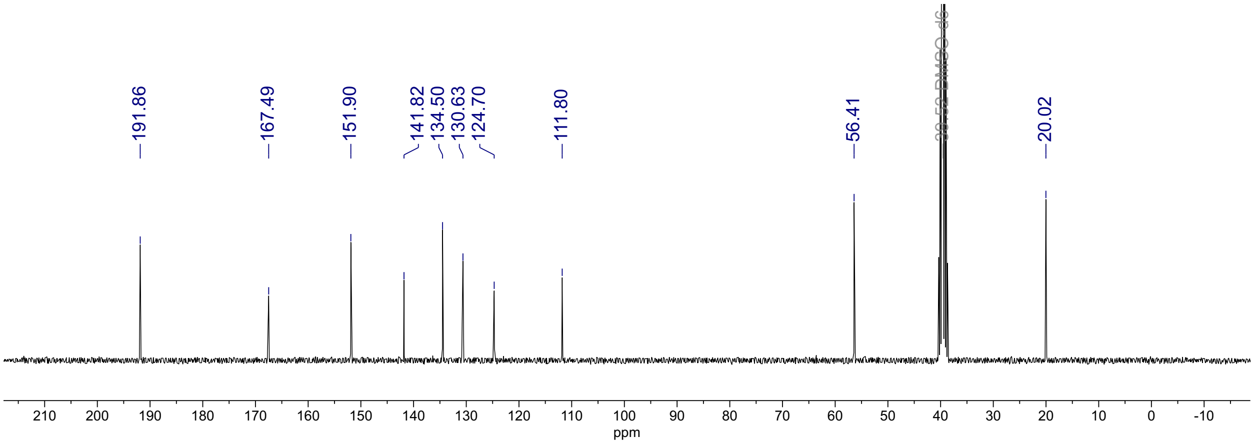

Figure S40. ^{13}C NMR spectrum of diacetyldehydrovanillin in DMSO-d_6

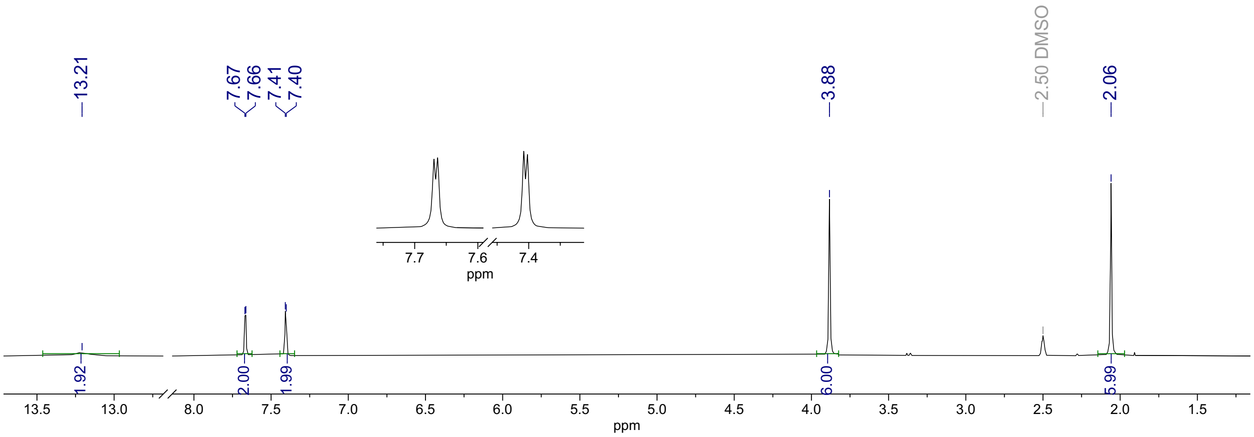

Figure S41. ^{1}H NMR spectrum of diacetyldehydrovanillic acid in DMSO-d_6

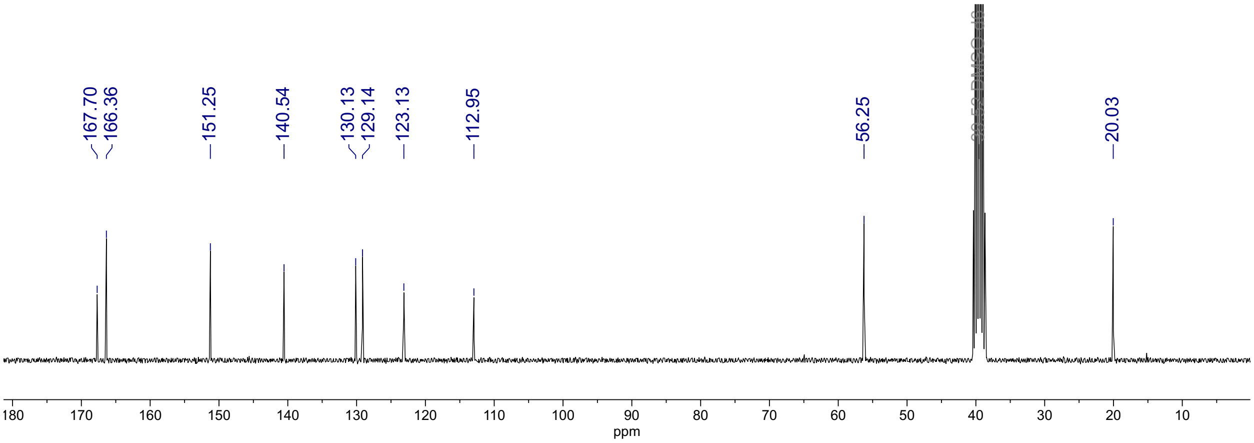

Figure S42. ^{13}C NMR spectrum of diacetyldehydrovanillic acid in DMSO-d_6

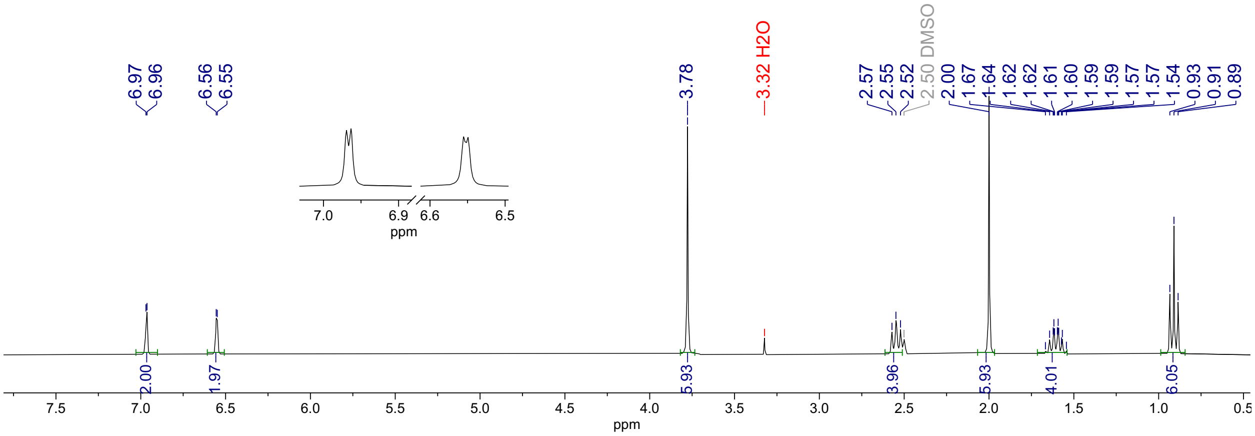

Figure S43. ^{1}H NMR spectrum of diacetyl tetrahydrodieugenol in DMSO-d_6

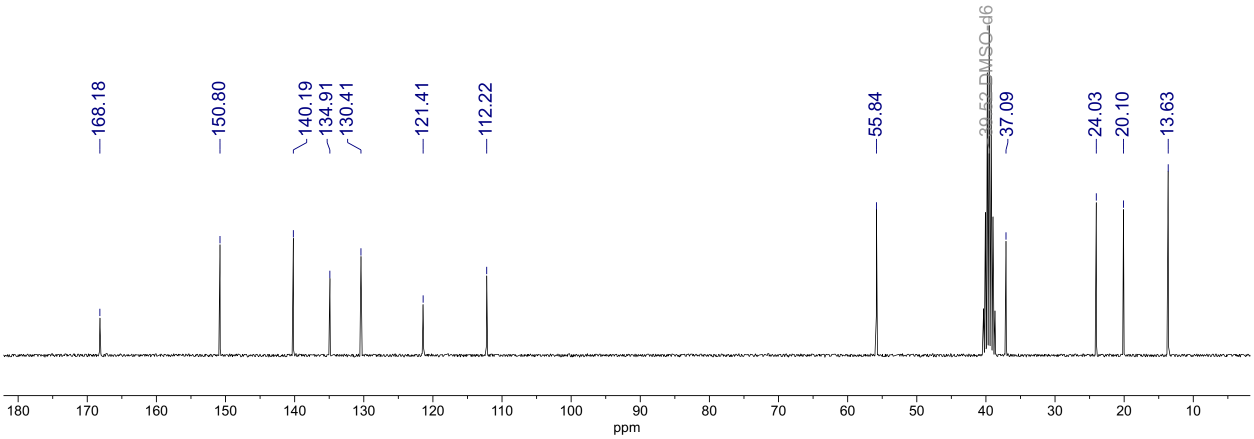

Figure S44. ^{13}C NMR spectrum of diacetyl tetrahydrodieugenol in DMSO-d_6

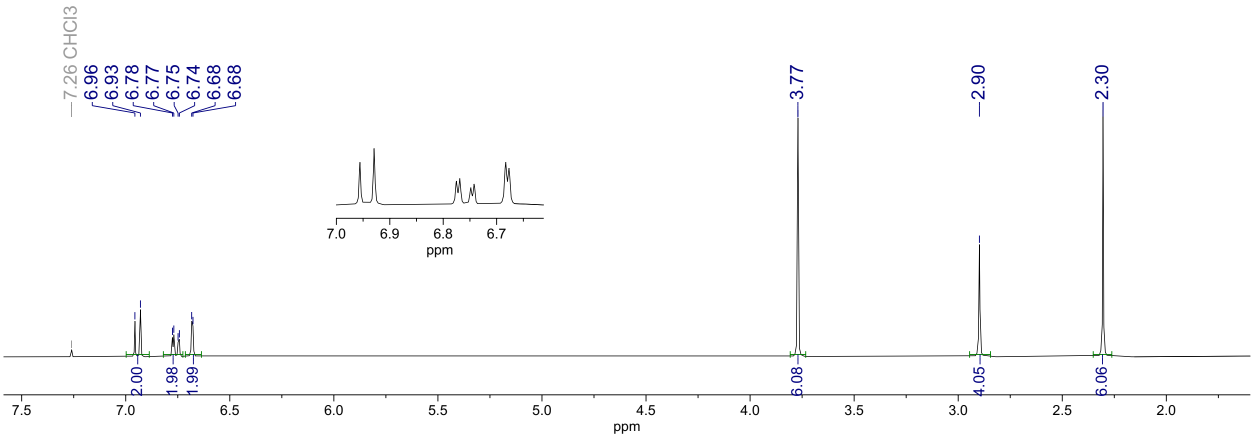

Figure S45. ^{1}H NMR spectrum of diacetyl diguaiacylethane in $CDCl_3$

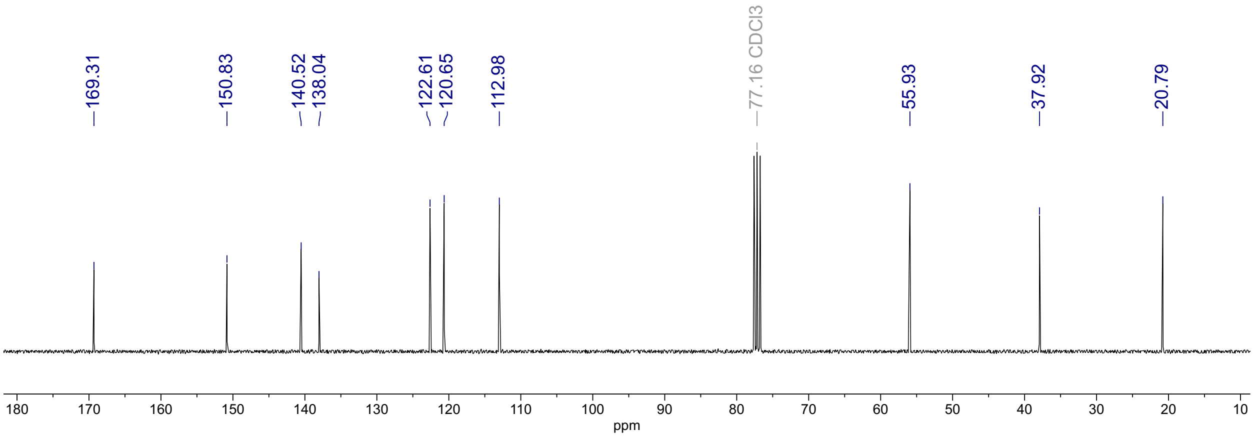

Figure S46. ^{13}C NMR spectrum of diacetyl diguaiacylethane in $CDCl_3$

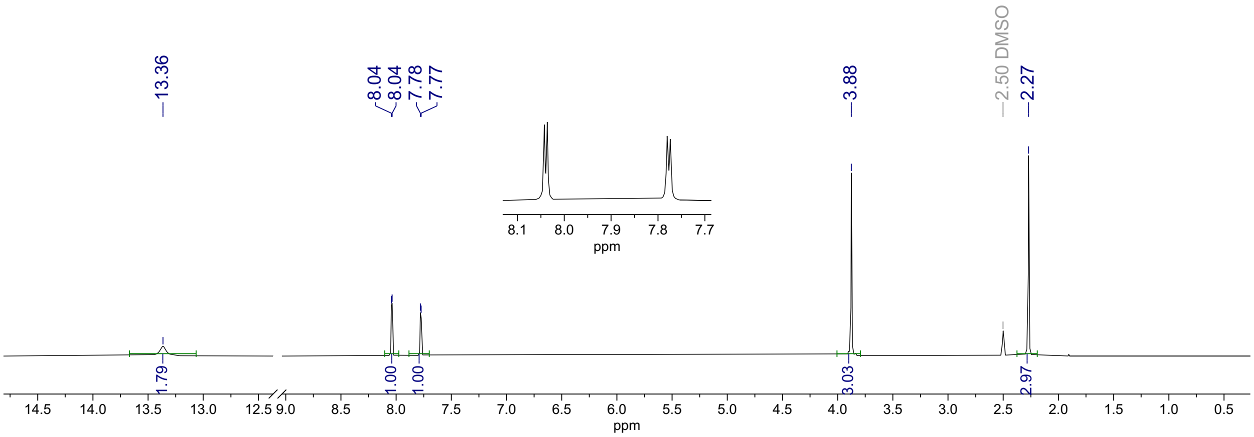

Figure S47. ^{1}H NMR spectrum of acetyl carboxyvanillic acid in DMSO-d_6

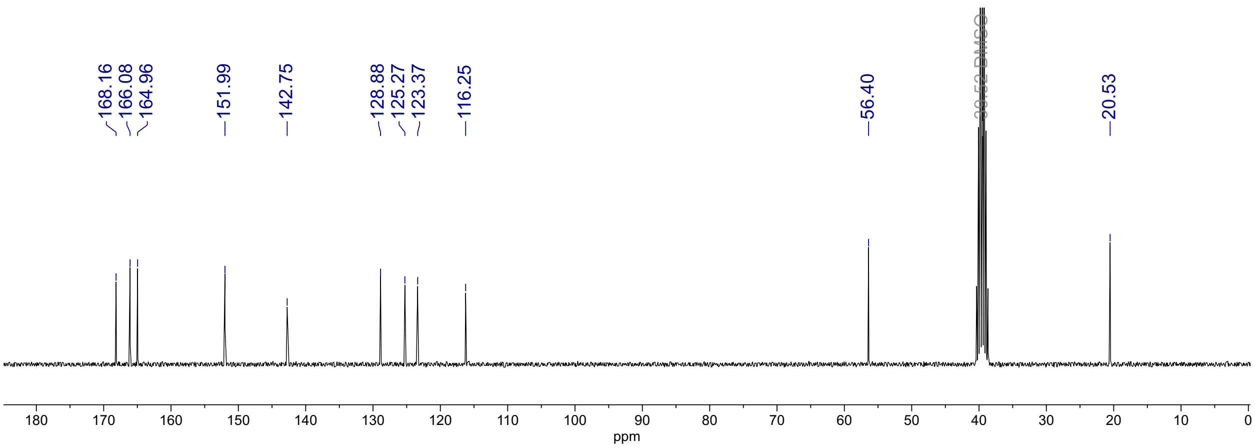

Figure S48. ^{13}C NMR spectrum of acetyl carboxyvanillic acid in DMSO-d_6

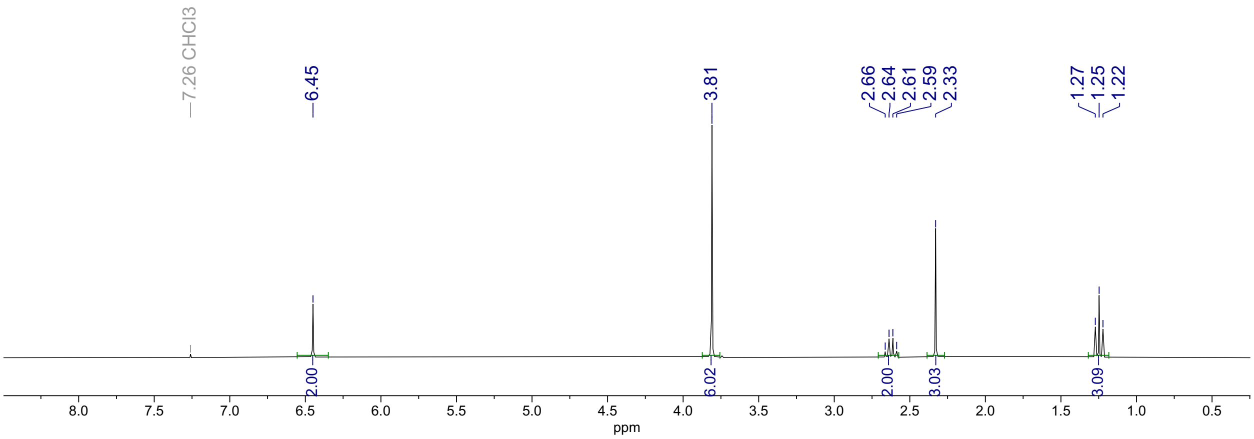

Figure S49. ^{1}H NMR spectrum of acetyl ethylsyringol in $CDCl_3$

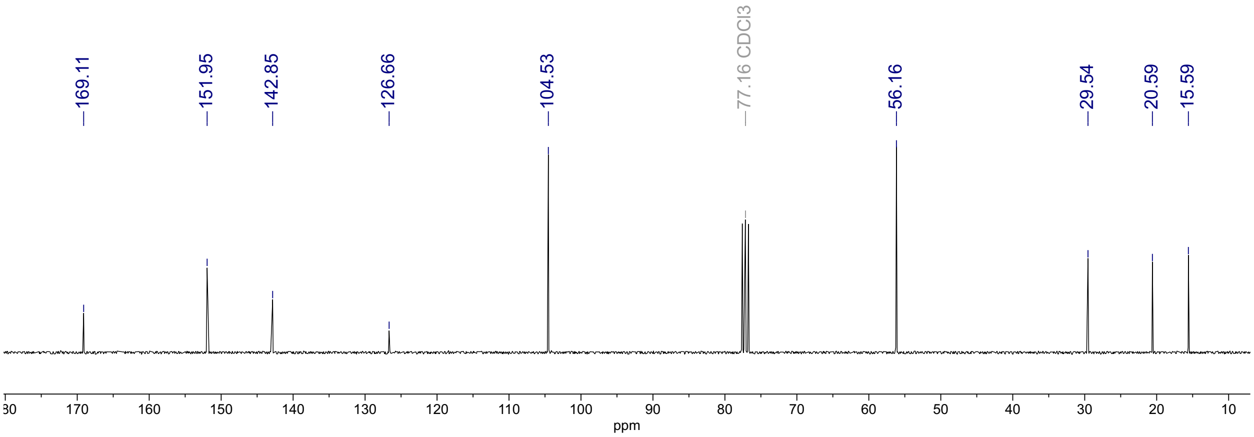

Figure S50. ^{13}C NMR spectrum of acetyl ethylsyringol in $CDCl_3$

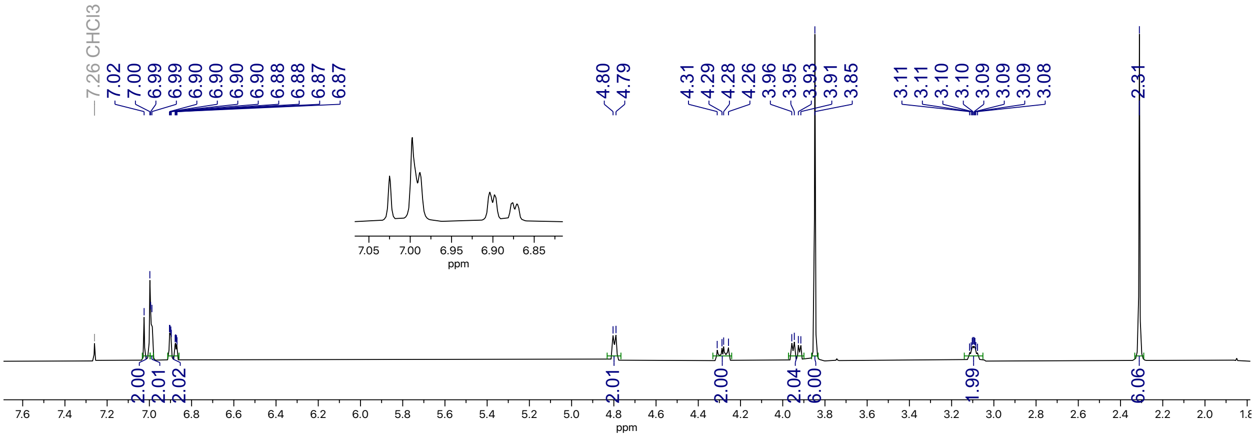

Figure S51. ^{1}H NMR spectrum of diacetyl pinoresinol in $CDCl_3$

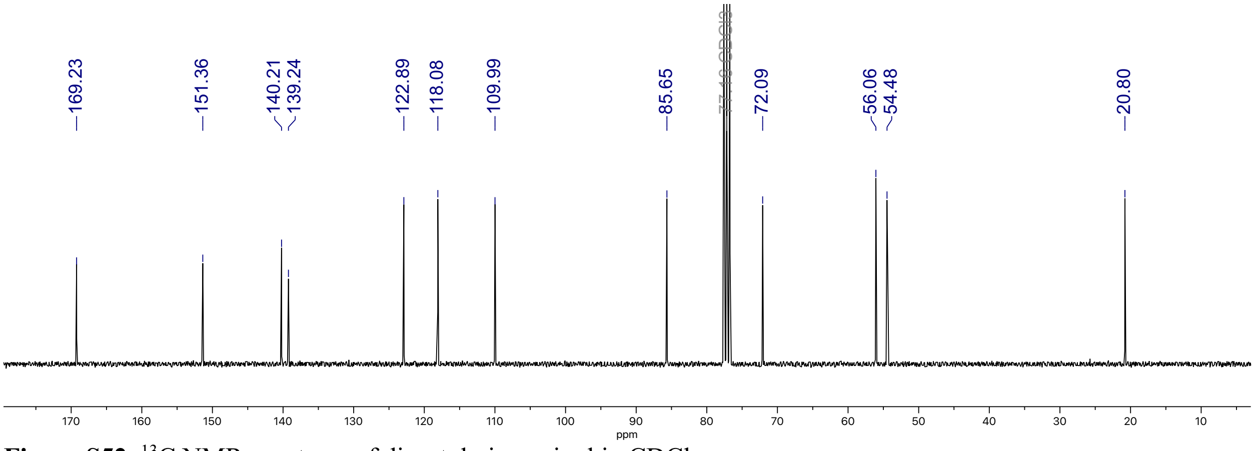

Figure S52. ^{13}C NMR spectrum of diacetyl pinoresinol in $CDCl_3$

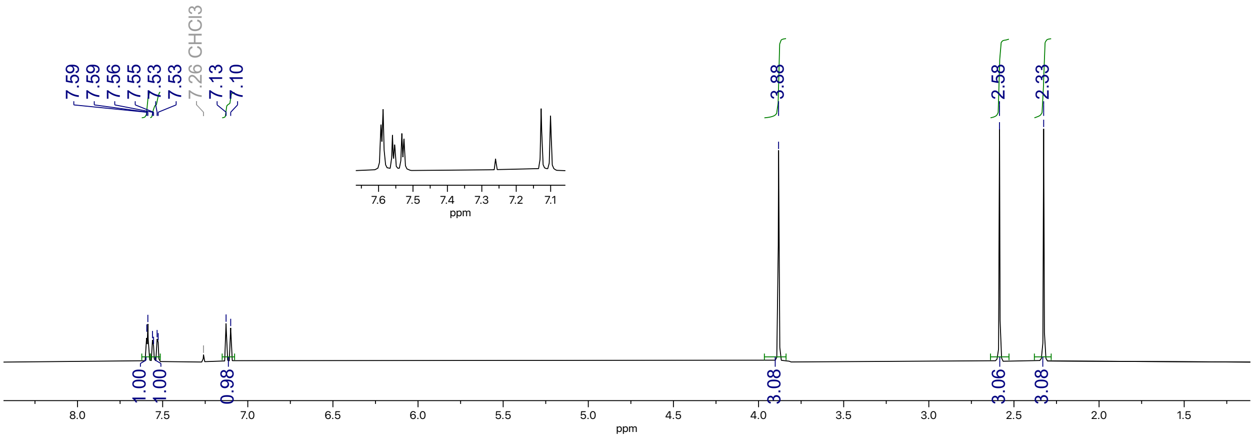

Figure S53. ^{1}H NMR spectrum of acetyl acetovanillone in $CDCl_3$

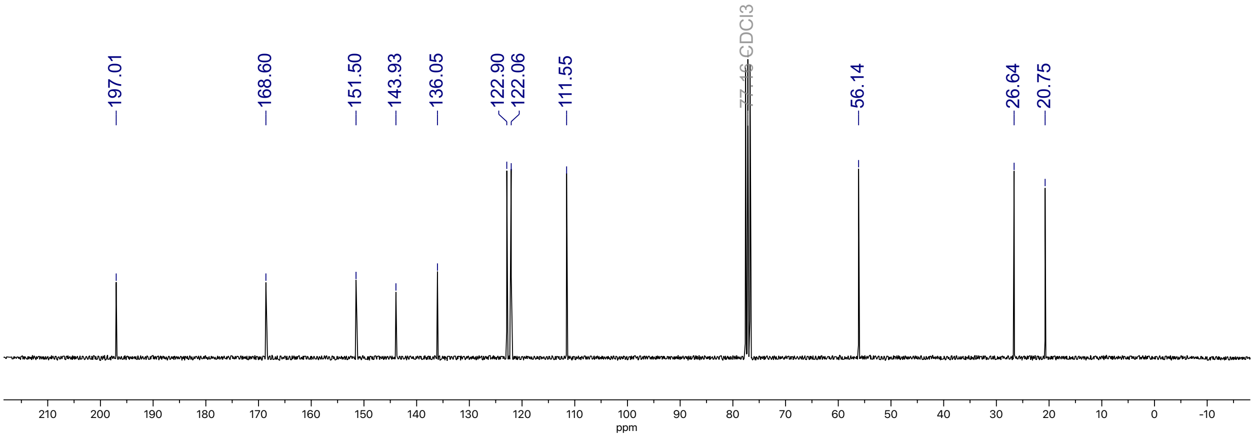

Figure S54. ^{13}C NMR spectrum of acetyl acetovanillone in $CDCl_3$

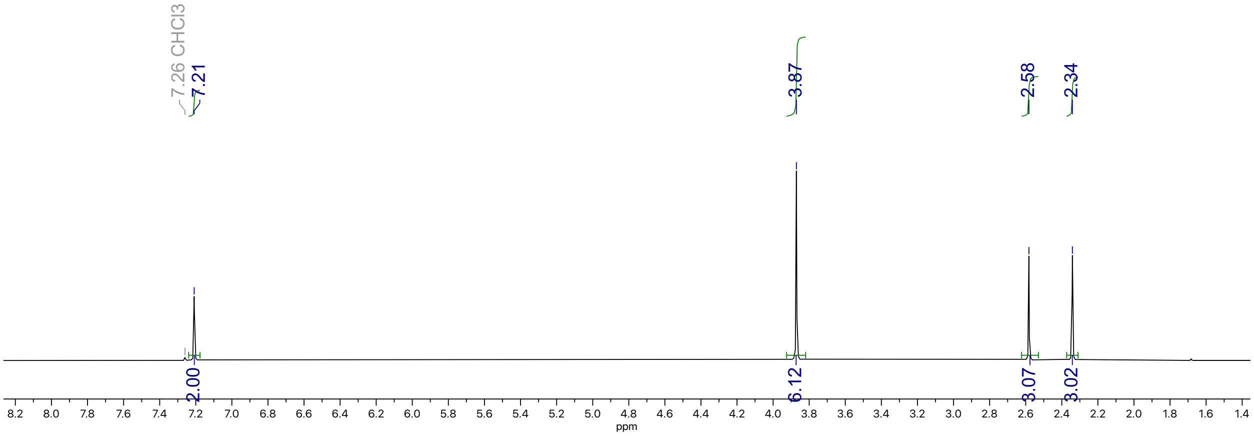

Figure S55. ^{1}H NMR spectrum of acetyl acetosyringone in $CDCl_3$

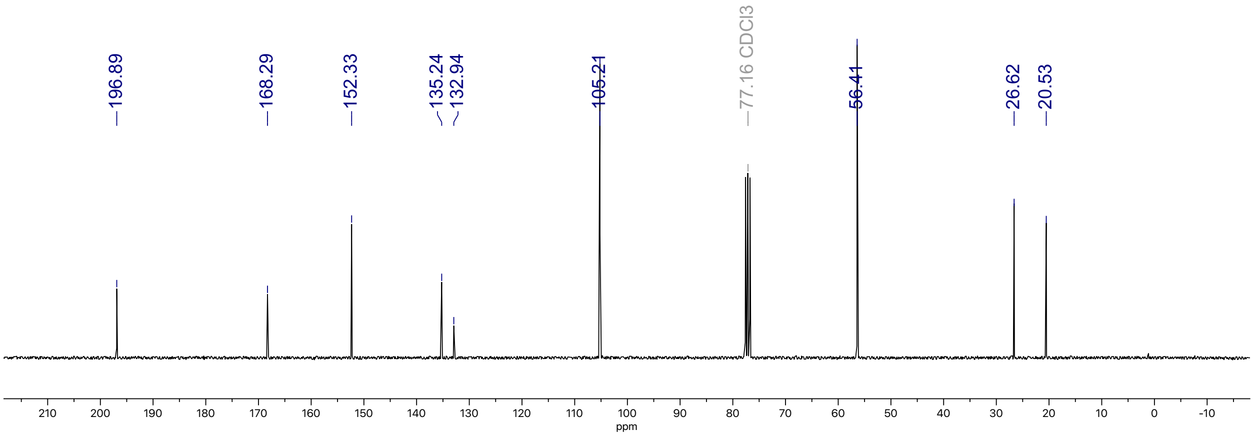

Figure S56. ^{13}C NMR spectrum of acetyl acetosyringone in $CDCl_3$

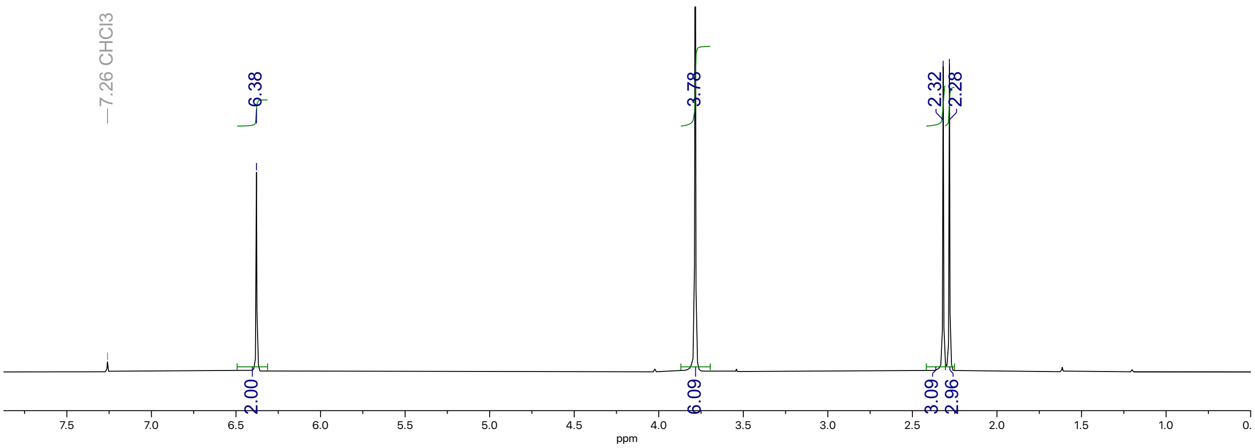

Figure S57. ^{1}H NMR spectrum of diacetyl dimethoxyhydroquinone in $CDCl_3$

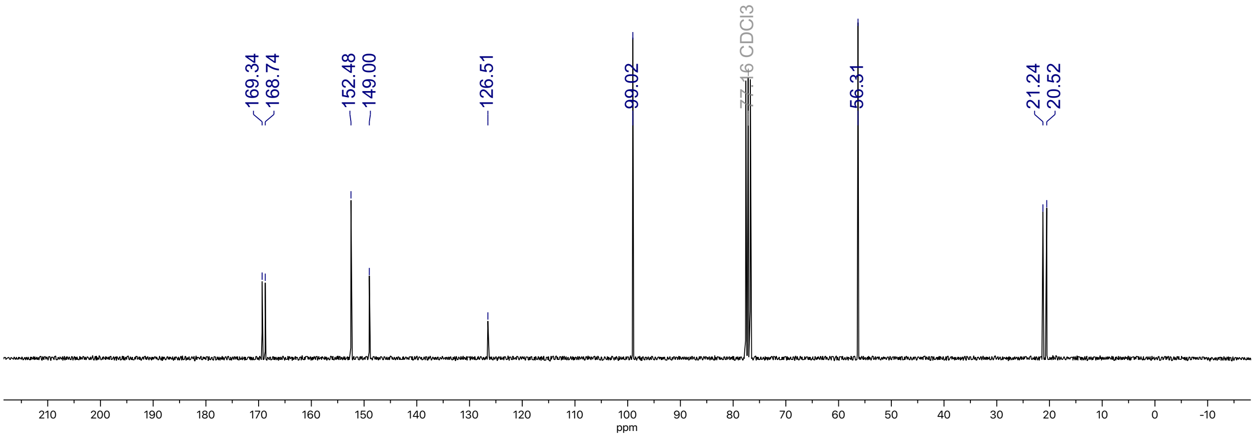

Figure S58. ^{13}C NMR spectrum of diacetyl dimethoxyhydroquinone in $CDCl_3$

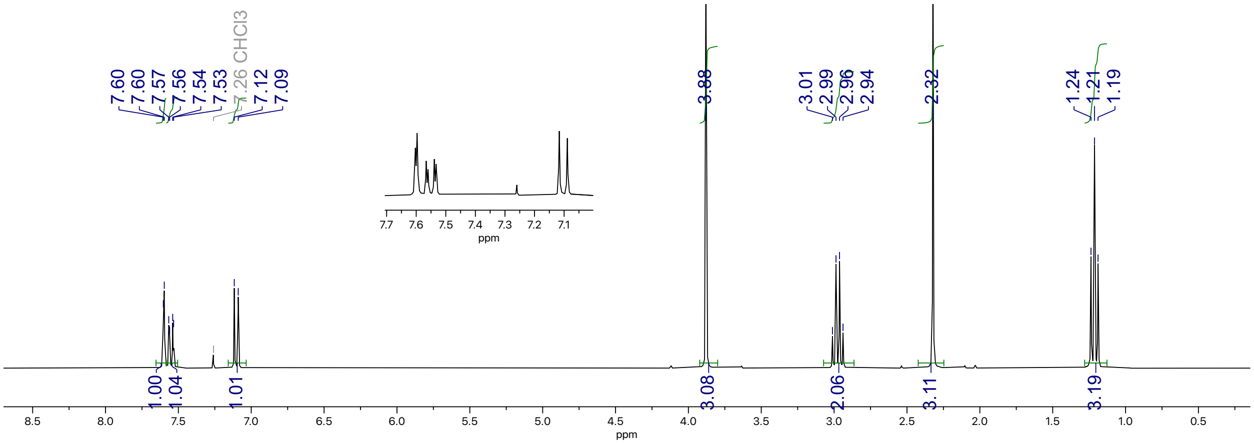

Figure S59. ^{1}H NMR spectrum of acetyl propiovanillone in $CDCl_3$

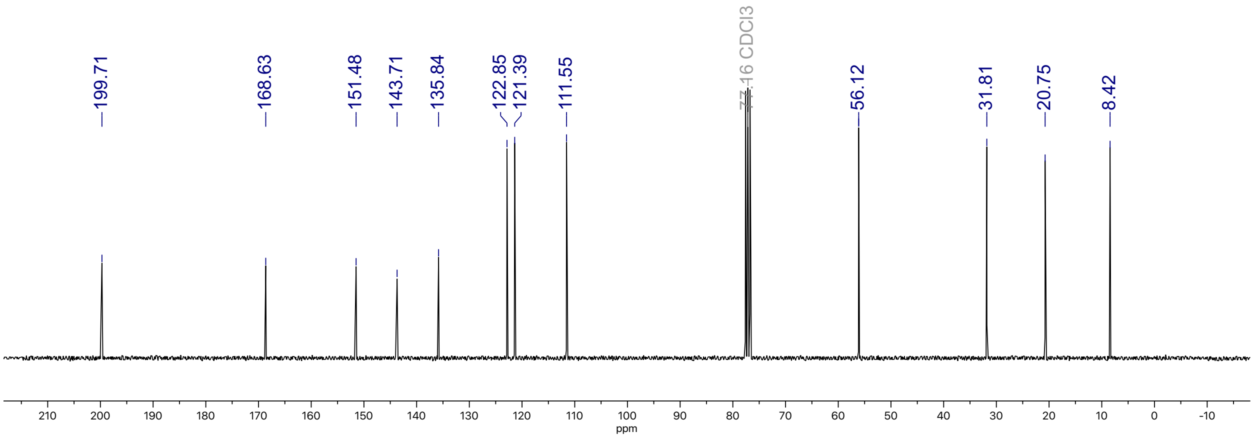

Figure S60. ^{13}C NMR spectrum of acetyl propiovanillone in $CDCl_3$

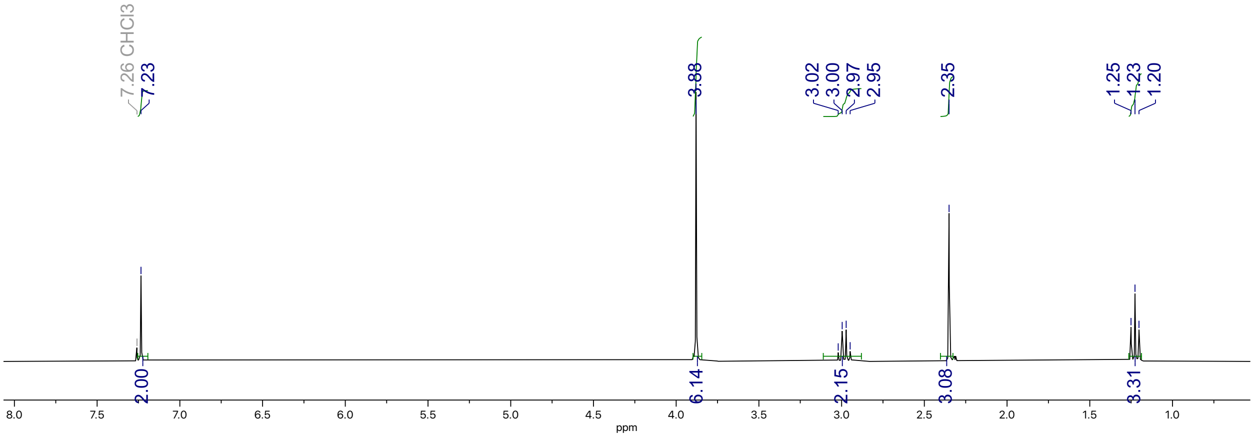

Figure S61. ^{1}H NMR spectrum of acetyl propiosyringone in $CDCl_3$

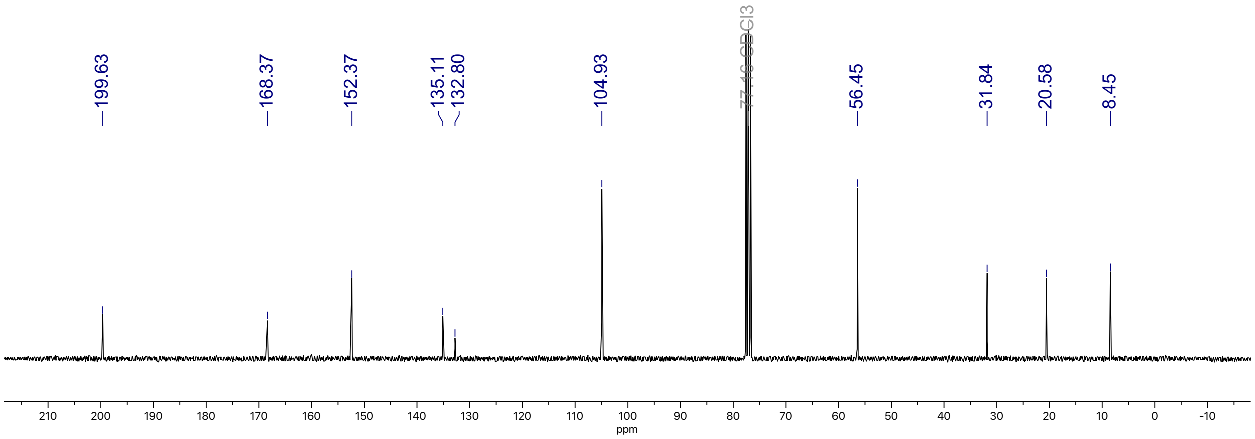

Figure S62. ^{13}C NMR spectrum of acetyl propiosyringone in $CDCl_3$

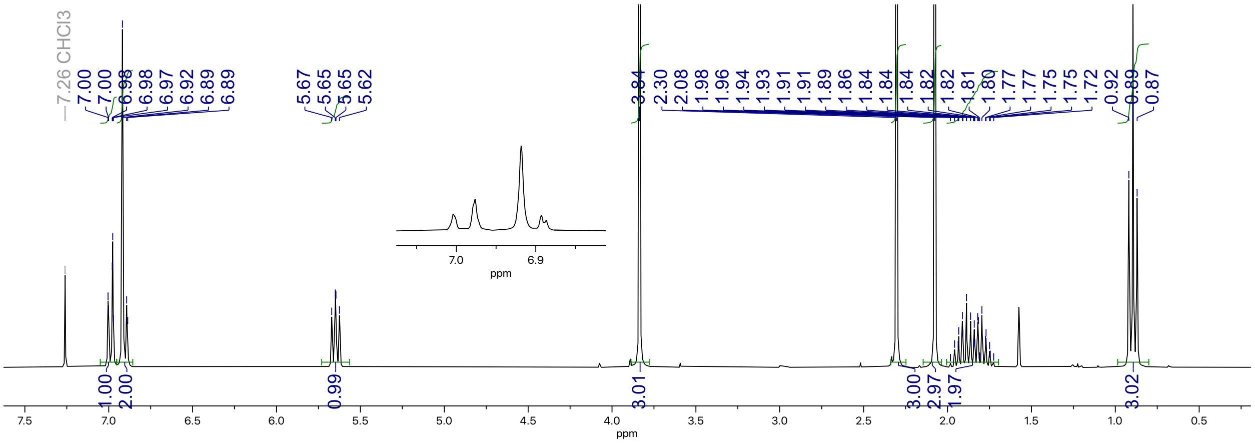

Figure S63. ^{1}H NMR spectrum of diacetyl a-guaiacylpropanol in $CDCl_3$

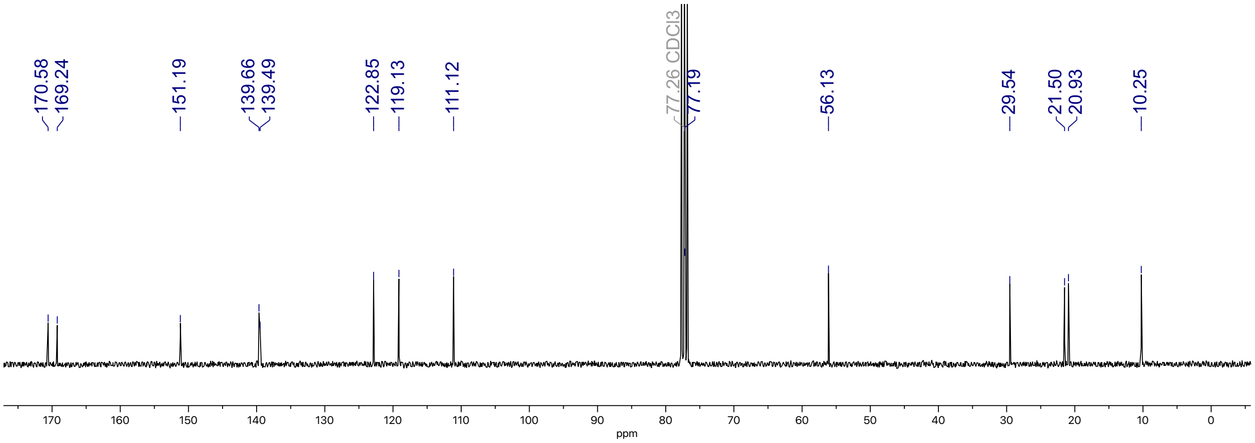

Figure S64. ^{13}C NMR spectrum of diacetyl a-guaiacylpropanol in $CDCl_3$

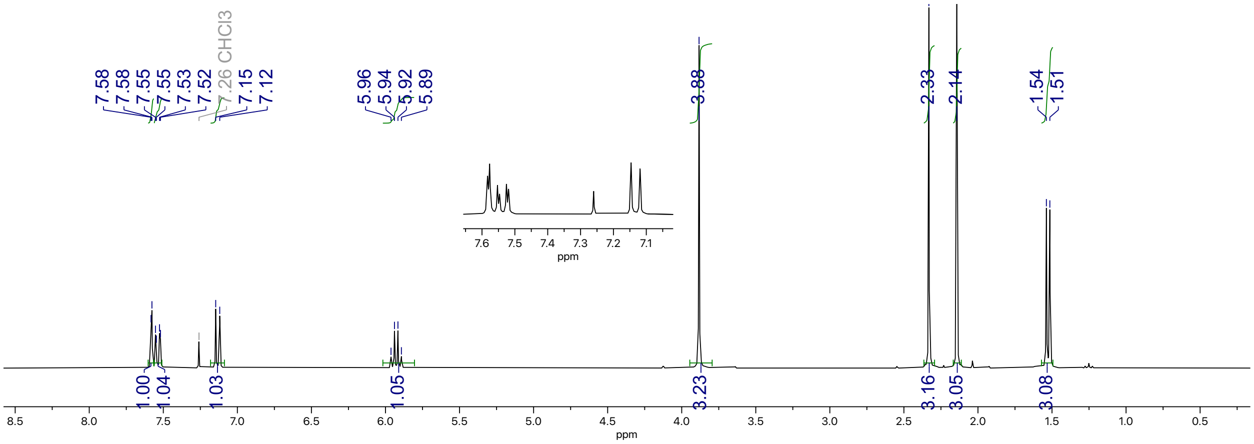

Figure S65. ^{1}H NMR spectrum of diacetyl a-hydroxypropiovanillone in $CDCl_3$

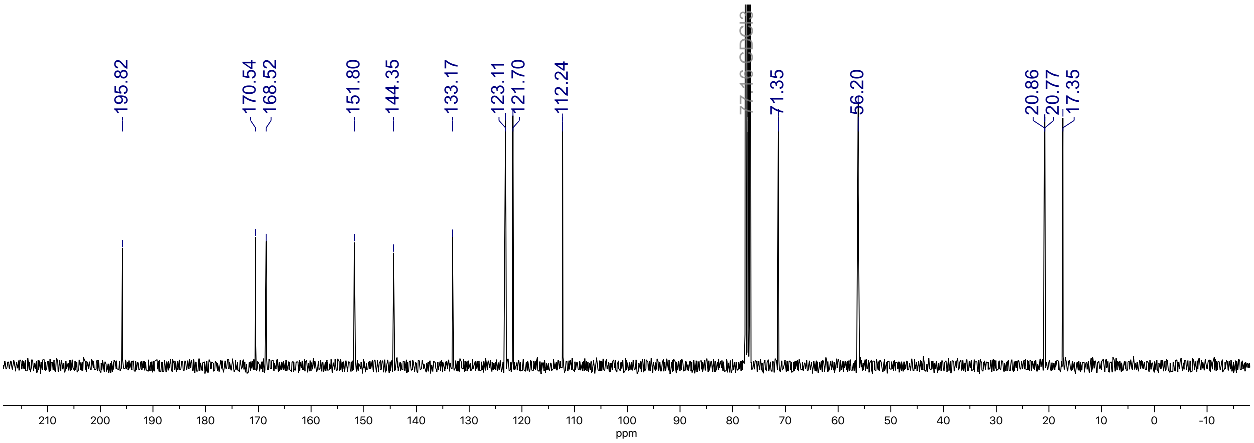

Figure S66. ^{13}C NMR spectrum of diacetyl a-hydroxypropiovanillone in $CDCl_3$

References

1. D. G. Brandner, J. S. Kruger, N. E. Thornburg, G. G. Facas, J. K. Kenny, R. J. Dreiling, A. R. C. Morais, T. Renders, N. S. Cleveland, R. M. Happs, R. Katahira, T. B. Vinzant, D. G. Wilcox, Y. Román-Leshkov and G. T. Beckham (2021). Flow-through solvolysis enables production of native-like lignin from biomass. *Green Chemistry 23*, 5437-5441.
2. M. K. Sahoo, S. B. Mhaske and N. P. Argade (2003). Facile routes to alkoxymaleimides/maleic anhydrides. *Synthesis 2003*, 0346-0349.
3. F. Yue, F. Lu, M. Regner, R. Sun and J. Ralph (2017). Lignin-derived thioacidolysis dimers: reevaluation, new products, authentication, and quantification. *ChemSusChem 10*, 830-835.
4. B. P. Pleitner, W. E. Michener, C. E. Payne and G. T. Beckham, (2019). Determination of cis, cis-and cis, trans-Muconic acid from biological conversion: Laboratory Analytical Procedure (LAP), National Renewable Energy Lab.(NREL), Golden, CO (United States).
5. X. Meng, C. Crestini, H. Ben, N. Hao, Y. Pu, A. J. Ragauskas and D. S. Argyropoulos (2019). Determination of hydroxyl groups in biorefinery resources via quantitative ^{31}P NMR spectroscopy. *Nat. Protoc. 14*, 2627-2647.
6. J. Etxebarria, H. Degenbeck, A.-S. Felten, S. Serres, N. Nieto and A. Vidal-Ferran (2009). Supramolecular-directed chiral induction in biaryl derivatives. *The Journal of Organic Chemistry 74*, 8794-8797.
7. X. Martin-Benlloch, M. Elhabiri, D. A. Lanfranchi and E. Davioud-Charvet (2014). A practical and economical high-yielding, six-step sequence synthesis of a flavone: application to the multigram-scale synthesis of ladanein. *Organic Process Research & Development 18*, 613-617.
8. S. Yamamura, Y. Terada, Y.-P. Chen, M. Hong, H.-Y. Hsu, K. Sasaki and Y. Hirata (1976). The structures of two novel neolignans, asatone and isoasatone. *Bulletin of the Chemical Society of Japan 49*, 1940-1948.
