# Supplementary material for: Autoxidation Catalysis for Carbon–Carbon Bond Cleavage in Lignin
Source: ACS Cent Sci. 2023 Nov 22;9(12):2277–85. doi: 10.1021/acscentsci.3c00813 (PMC10755848; doi:10.1021/acscentsci.3c00813)
Supplement: Supplementary file 2 — oc3c00813_si_002.pdf [file oc3c00813_si_002.pdf]

oc-2023-00813z.R1

Name: Peer Review Information for "Autoxidation catalysis for carbon–carbon bond cleavage in lignin"

First Round of Reviewer Comments

Reviewer: 1

Comments to the Author

Beckham, Stahl and co-workers have prepared a manuscript titled 'Autoxidation catalysis for carbon–carbon bond cleavage in lignin' describing a novel approach of utilizing Co/Mn/Br-based catalyst to promote autoxidation of lignin. The state-of-the-art approach, reductive catalytic fractionation, targets C-O bond cleavage, while the authors showed their methods can facilitate C-C bond cleavage instead, therefore giving higher yields of aromatics. Instead of designing new catalysts, the authors applied an existing oxidation catalytic system in industry (para-xylene to terephthalic acid process) to an unsolved problem, lignin depolymerization, and they showed promising results.

In summary, this manuscript contributes new knowledge to the field of lignin degradation while simultaneously expanding the footprint of the reductive catalytic fractionation process and utilization of raw material lignin. It is a well-investigated project. The manuscript is presented with solid data and communicated clearly. I have some suggestion below and hope they will make the manuscript better.

1) The lignin field has alternative oxidation strategies. For example, Stahl has showed that TEMPO-mediated beta-O-4 oxidation. What is the comparison between these two methods? What are the benefits of the catalytic autoxidation approach?

2) Those reaction schemes have the same conditions, I recommend redraw these figures, and make a tabulated data including several columns: substrate, conditions, products, and results. These will make them much nicer.

Reviewer: 2

Comments to the Author

The manuscript by Gu and coworkers describes the conversion of lignin to muconic acid through a series of reactions including fractionation to produce lignin oligomers (+monomers), acetylation to form acetylated oligomers (+monomers), removal of monomers to avoid monomer degradation, the catalytic autoxidation (central focus) and bioconversion of products to muconic acid (secondary focus). While the conditions used here have been used in previous studies, the demonstration of oxidative degradation on lignin oligomers makes this approach more advantageous and successful. The authors examine some model studies to predict the product outcomes from the RCF oils, some degradation studies to determine the degradation pathways of the products (initially the product yields are lower than the starting content of the RCF oils). Ultimately, this results in the authors separating out the monomers from the RCF oil to study the degradation of the oligomers/dimers. A single catalyst system with some investigation into catalyst loading/temperature provides optimal yield resulting in a 17% increase of monomers. The authors then subject the oxidation products to Pseudomonas putida cultures to produce muconic acid. The work is thorough and rigorous, but more experiments are needed to bring this to the level expected for ACS Central Science; as is, this report would be more suitable for ACS Sustainable Chemistry & Engineering.

-Did the authors subject the RCF oils to Pseudomonas putida cultures in the absence of the acetylation/oxidation protocol? Given the modest yields from oxidation, perhaps direct bioconversion could produce similar or higher yields of muconic acid.

-There is limited mechanistic analysis, rationalization for product outcomes (i.e. 5, what is the mass balance, di-carboxylic acids, no activation at the bridge position?)

-Did the authors try NaCl, rather than NaBr, or something else with a larger BDE? A better C–H abstractor might facilitate conversion of oligomers to products. Unless the authors hypothesize that oxygen-centered radicals are primarily involved in the C–H abstraction step.

-Can the authors hypothesize a mechanism for acetate deprotection? The distillation step to separate out monomers is undesirable and a major limitation, as the authors state in their conclusion; could the authors use a mixture of acetic acid/acetic anhydride as the solvent?

-Schemes 1-3 are redundant with regards to “same conditions” listed above everything but the first arrow. Perhaps there is a cleaner way to present these, with a generic scheme and reactants/products listed underneath.

Reviewer: 3

Comments to the Author

The authors present a great study showing how a a side-stream from lignin depolymerization in the "lignin-first" approach, that is RCF, can be converted to a mixture that can be addressed by biological funneling. Here, this entails an oligomeric fraction obtained after acetylation and removal of valuable monomers by distillation, which is subsequently oxidized using Co/Mn/Br-based catalytic autooxidation. The product mixture from the auto oxidation could be converted to cis,cis-muconic acid using P. putida previously studied by some of the authors involved. Overall this study displays an innovative approach to utilize this otherwise low-value side stream obtained from a catalytic lignin conversion process that has the potential to be scaled in the near future. There can be some concerns raised over the overall efficiency and the "greeness". But as a proof of concept this paper is the first of its kind establishing the possibility to link this bio-based chemical side stream to biological funneling. This can be a great starting point for the development of new innovative approaches for the infidividual steps to reach an overall sustainable and green process for converting lignocellulosic streams in emerging biorefining schemes. These aspects combining different fields of catalytic conversion and biological funneling make this manuscript highly suitable for publication in ACS Central Science.

Author's Response to Peer Review Comments:

Reviewer comments are provided in black font. Our responses are provided in blue font.

**Reviewer 1**

Beckham, Stahl and co-workers have prepared a manuscript titled 'Autoxidation catalysis for carbon–carbon bond cleavage in lignin' describing a novel approach of utilizing Co/Mn/Br-based catalyst to promote autoxidation of lignin. The state-of-the-art approach, reductive catalytic fractionation, targets C-O bond cleavage, while the authors showed their methods can facilitate C-C bond cleavage instead, therefore giving higher yields of aromatics. Instead of designing new catalysts, the authors applied an existing oxidation catalytic system in industry (para-xylene to terephthalic acid process) to an unsolved problem, lignin depolymerization, and they showed promising results.

In summary, this manuscript contributes new knowledge to the field of lignin degradation while simultaneously expanding the footprint of the reductive catalytic fractionation process and utilization of raw material lignin. It is a well-investigated project. The manuscript is presented with solid data and communicated clearly. I have some suggestion below and hope they will make the manuscript better.

We thank the reviewer for the positive response to our work and for the constructive comments, which we have addressed below and through edits to the manuscript.

1) The lignin field has alternative oxidation strategies. For example, Stahl has showed that TEMPO-mediated beta-O-4 oxidation. What is the comparison between these two methods? What are the benefits of the catalytic autoxidation approach?

The referenced study from Stahl *et al.* focused on ether bond cleavage, and we do not anticipate that this chemistry (TEMPO oxidation followed by formic acid-catalyzed ether bond cleavage) would be able to cleave C–C bonds, which was the focus of the current study.

More generally, a comparative study with other lignin oxidation strategies is outside the scope of the present study, but we agree that such studies would be valuable and will be pursued in future work. We chose to study catalytic autoxidation in this work given its industrial relevance. The catalytic conditions used in this study (with acetic acid, 2 h residence time, and 120 °C with Co/Mn/Br) is directly inspired by the Amoco process conditions for the manufacture of terephthalic acid from *p*-xylene at ~80 MMT per year scale, here conducted with a lower temperature. By demonstrating that lignin oxidation can indeed be achieved with similar conditions, we aim to further develop this process toward industrial viability.

Previous research on Amoco oxidation conditions indicate that acetic acid can undergo degradation to CO or $CO_2$, but the losses are minimal even when run up to 175-225 °C, which, as we show in our work, is higher than the temperature needed for C–C bond cleavage in lignin (Sheehan, R.J. *Ullmann's Encyclopedia of Industrial Chemistry*, 2012). It is also known that the Co/Mn/Br oxidation catalyst can be re-used for many years with little makeup (Tomás, R. A. F. *et al. Chem. Rev.* **2013**, *113*, 7421-7469). We are optimistic that the same beneficial features will be true for the present system. Work is ongoing to further optimize this process for lignin now, including transitioning to a flow-based process.

2) Those reaction schemes have the same conditions, I recommend redraw these figures, and make a tabulated data including several columns: substrate, conditions, products, and results. These will make them much nicer.

We thank the reviewer for this suggestion and have updated Schemes 1-3 to a table format, as shown in the uploaded copy with red highlights.

**Reviewer 2**

The manuscript by Gu and coworkers describes the conversion of lignin to muconic acid through a series of reactions including fractionation to produce lignin oligomers (+monomers), acetylation to form acetylated oligomers (+monomers), removal of monomers to avoid monomer degradation, the catalytic autoxidation (central focus) and bioconversion of products to muconic acid (secondary focus). While the conditions used here have been used in previous studies, the demonstration of oxidative degradation on lignin oligomers makes this approach more advantageous and successful. The authors examine some model studies to predict the product outcomes from the RCF oils, some degradation studies to determine the degradation pathways of the products (initially the product yields are lower than the starting content of the RCF oils). Ultimately, this results in the authors separating out the monomers from the RCF oil to study the degradation of the oligomers/dimers. A single catalyst system with some investigation into catalyst loading/temperature provides optimal yield resulting in a 17% increase of monomers. The authors then subject the oxidation products to Pseudomonas putida cultures to produce muconic acid. The work is thorough and rigorous, but more experiments are needed to bring this to the level expected for ACS Central Science; as is, this report would be more suitable for ACS Sustainable Chemistry & Engineering.

We thank the reviewer for the positive response to our work and for the constructive comments, which we have addressed below and through edits to the manuscript.

-Did the authors subject the RCF oils to Pseudomonas putida cultures in the absence of the acetylation/oxidation protocol? Given the modest yields from oxidation, perhaps direct bioconversion could produce similar or higher yields of muconic acid.

We appreciate this idea from the reviewer, given that monomers are indeed present alongside the dimers and oligomers found in RCF oil. As we described in a perspective from 2016, lignin oxidation lends itself to biological funneling much more so than lignin-derived substrates from reductive chemistry (Beckham, G.T. *et al., Current Opin. Biotechnol.* **2016**, 42, 40-53).

For RCF oil specifically, the use of this substrate for *P. putida* presents two major challenges:

First, our group and others have shown that RCF oil is highly toxic to *P. putida* and other bacteria, likely due to the presence of toxic solvents and aromatics derived from reduced lignin (Wu, Y. *et al*., *Chem Eng J*, **2023**, *452,* 139267). In fact, RCF oil has been proposed as an industrial antimicrobial due to its strong bacteriostatic effect against pathogens like *Staphylococcus aureus*, even at concentrations as low as 0.25 wt% (Ebikade, E.O. *et al*., *Green Chem*, **2020,** *22,* 7435-7447).

Additionally, the oil itself is poorly soluble in water, making it difficult to incorporate into the aqueous media used for bacterial cultivation. In studies where RCF oil has been applied as a growth substrate for bacteria, it was solubilized in DMSO (which is also toxic to *P. putida*) and could only be added to aqueous media at a low concentration of 250 ppm (Fetherolf, M.M. *et al*., *Proc Natl Acad Sci USA*, **2020**, *117*, 25771-25778).

-There is limited mechanistic analysis, rationalization for product outcomes (i.e. 5, what is the mass balance, dicarboxylic acids, no activation at the bridge position?)

We agree that mechanistic studies and further investigation into product profiles could play an important role in future optimization of this process and will be pursued in future work. Importantly, given the similarity between the conditions used here and the commercial Amoco process, we expect the central mechanistic features to remain the same between the two systems (see refs. 26 and 27).

-Did the authors try NaCl, rather than NaBr, or something else with a larger BDE? A better C–H abstractor might facilitate conversion of oligomers to products. Unless the authors hypothesize that oxygen-centered radicals are primarily involved in the C–H abstraction step.

We selected the Co/Mn/Br catalyst for this work given its relevance to industrial autoxidation processes, as noted above. The C–H abstraction step under such autoxidation conditions is believed to involve both Br- and oxygencentered radicals (Tomás, R. A. F. *et al. Chem. Rev.* **2013**, *113*, 7421-7469). The assessment of different radical mediators, such as NaCl, is outside the scope of the present study, but we agree with the reviewer that such studies could be valuable and will be pursued in future work.

-Can the authors hypothesize a mechanism for acetate deprotection? The distillation step to separate out monomers is undesirable and a major limitation, as the authors state in their conclusion; could the authors use a mixture of acetic acid/acetic anhydride as the solvent?

Under Amoco autoxidation conditions, $O_2$ is the terminal oxidant and generates water as a byproduct. Hydrolysis of the acetyl groups is a likely pathway for deprotection, and we have updated the discussion on pg. 3 of the main text to note this: "Deprotection of the acetyl groups likely occurs through hydrolysis, as water is a byproduct under AMOCO oxidation conditions.[22]"

We are interested in exploring the use of acetic acid/acetic anhydride solvent for the *in situ* protection of phenol groups, which will be pursued in future work.

-Schemes 1-3 are redundant with regards to “same conditions” listed above everything but the first arrow. Perhaps there is a cleaner way to present these, with a generic scheme and reactants/products listed underneath. We thank the reviewer for this suggestion and have updated Schemes 1-3 to table format.

**Reviewer 3**

The authors present a great study showing how a a side-stream from lignin depolymerization in the "lignin-first" approach, that is RCF, can be converted to a mixture that can be addressed by biological funneling. Here, this entails an oligomeric fraction obtained after acetylation and removal of valuable monomers by distillation, which is subsequently oxidized using Co/Mn/Br-based catalytic autooxidation. The product mixture from the auto oxidation could be converted to cis,cis-muconic acid using P. putida previously studied by some of the authors involved. Overall this study displays an innovative approach to utilize this otherwise low-value side stream obtained from a catalytic lignin conversion process that has the potential to be scaled in the near future. There can be some concerns raised over the overall efficiency and the "greeness". But as a proof of concept this paper is the first of its kind establishing the possibility to link this bio-based chemical side stream to biological funneling. This can be a great starting point for the development of new innovative approaches for the infidividual steps to reach an overall sustainable and green process for converting lignocellulosic streams in emerging biorefining schemes. These aspects combining different fields of catalytic conversion and biological funneling make this manuscript highly suitable for publication in ACS Central Science.

We thank the reviewer for the positive response to our work and for the constructive comments.
